# β,β-Difluoro Peroxides as Fluorinated C2-Building Blocks for the Construction of Functionalized Indolizines

**DOI:** 10.3390/molecules29245927

**Published:** 2024-12-16

**Authors:** Yangyang Ma, Hua Zhang, Xiaoxiao Du, Shurui Fang, Kexin Yin, Gangfeng Du, Zhengshan Tian, Zhonghao Zhou

**Affiliations:** 1 School of Chemical & Environmental Engineering, Pingdingshan University, Pingdingshan 467000, China; 17597989566@163.com (X.D.); f19839393046@163.com (S.F.); 17837521986@163.com (K.Y.); 27138@pdsu.edu.cn (G.D.); tianzhengshan@163.com (Z.T.); 2 College of Medicine, Pingdingshan University, Pingdingshan 467000, China; zhanghua6112@126.com; 3 School of Material Science and Engineering, Dalian Jiaotong University, Dalian 116028, China

**Keywords:** β,β-difluoro peroxides, fluorinated C2-building blocks, functionalized indolizines

## Abstract

An effective method for the construction of functionalized indolizines has been developed in which β,β-difluoro peroxides act as novel C2-building blocks to implement [3+2] annulation with pyridinium ylides under base-mediated conditions. With this protocol, a broad range of multisubstituted indolizines were prepared in moderate to good yields under mild conditions, and many useful functional groups were tolerated.

## 1. Introduction

The incorporation of a fluorine or perfluoroalkyl fragment into organic molecules can dramatically alter their physicochemical and biological properties [1,2,3]. The construction of fluorinated heterocycles has always been of particular interest in drug design due to fluorine’s unique electronic and steric properties which can enhance the lipophilicity, metabolic stability, and bioavailability of drug candidates [4,5,6]. To this end, significant advances have been made in the preparation of fluoroalkyl-substituted organic skeletons with diverse fluoroalkylated reagents [7,8,9,10,11,12]. Nevertheless, the direct fluoroalkylation of N-heterocycles in the desired position is still difficult [13,14]. Therefore, by employing simple and readily accessible fluorine-containing moieties as foundational units, more adaptable synthetic strategies serve as crucial alternatives to the direct fluoroalkylation approach. This highlights the superior utility and significance in these alternative methodologies [15,16].

**Figure 1:**
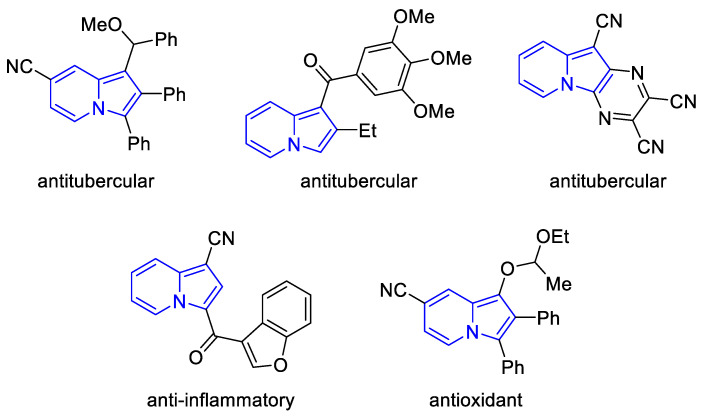
Representative bioactive indolizine derivatives.

**Scheme 1:**
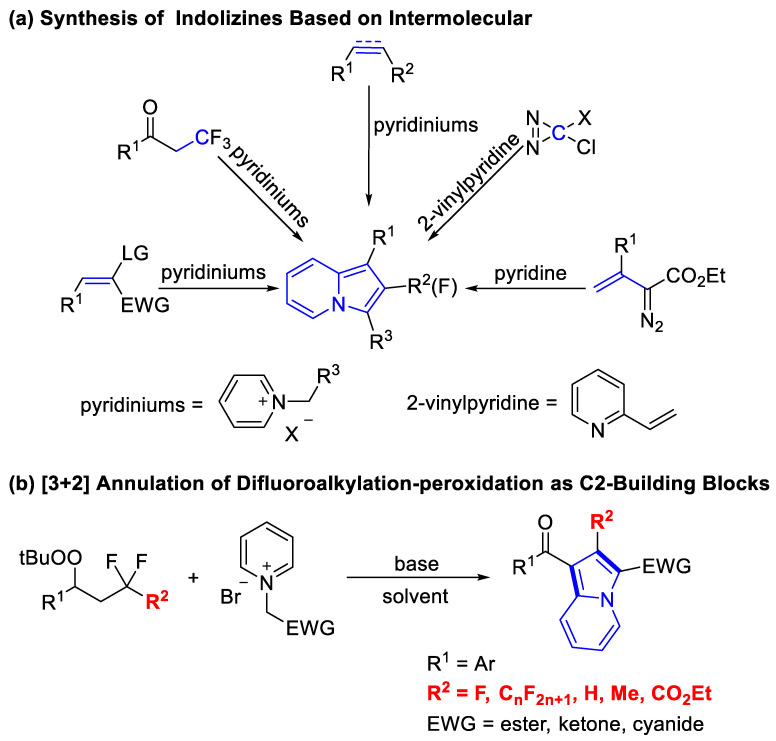
Representative protocols for the intermolecular synthesis of indolizines.

Indolizines, a class of nitrogen-containing heterocycles with 10 π-delocalized electrons [17], have been extensively studied for their molecular structure and applications [18,19,20]. Indolizine derivatives also exhibit a broad spectrum of biological activities, including antitubercular, anticancer, antineoplastic, anti-inflammatory, and antioxidant properties [21,22,23,24,25] (Figure 1). Given the significant value of this structure, chemists have made considerable efforts and have developed synthetic approaches to achieve its synthesis and functionalization [26,27]. One of the most frequently utilized approaches for indolizines was based on the intermolecular [3+2] cycloaddition of 2-pyridylacetates or pyridiniums with activated alkenes, alkynes [28,29,30,31,32,33,34] or α-CF_3_ ketones [35]. Meanwhile, the reaction of 2-vinylpyridines with dichlorocarbene [36] and the cyclization of pyridines with alkenyl diazo acetates etc. [37,38] are also effective methods for the construction of indolizines (Scheme 1a). Even so, there are still limited available methods for the synthesis of perfluoroalkyl-substituted indolizines.

In this context, our work introduces a novel and efficient method for the synthesis of functionalized indolizines through oxidative [3+2] annulation of β,β-difluoro peroxides with pyridinium salts. This approach utilizes β,β-difluoro peroxides as fluorine-containing C2-building blocks, providing a straightforward and regioselective route to a wide range of substituted indolizines (Scheme 1b). The use of β,β-difluoro peroxides as building blocks [39,40,41] offers several advantages, including their easy availability and low cost, and the ability to introduce different functional groups into the indolizine skeleton. This method opens up new possibilities for the rapid and scalable synthesis of fluorinated indolizines, paving the way for the development of novel bioactive compounds and pharmaceuticals.

## 2. Results and Discussion

At the outset of our investigation, we selected β-perfluoroalkyl peroxide **1a** and 1-(2-ethoxy-2-oxoethyl) pyridinium bromide **2a** as the model to optimize the reaction conditions (Table 1). To our delight, the desired product **3a** was obtained in a 54% yield in the presence of five equivalents of 1,4-diazabicyclo [2.2.2] octane (DABCO) as base in MeCN (Entry 1). Further screening of bases, such as *N*,*N*-diisopropylethylamine (DIPEA), triethylamine (NEt_3_), potassium phosphate tribasic (K_3_PO_4_), cesium carbonate (Cs_2_CO_3_), and potassium hydroxide (KOH), led to poor results (entries 2–6, respectively). These outcomes indicated DABCO (*p*Ka = 8.8) [42] was exactly adequate to modulate the rate of Kornblum–DeLaMare rearrangement of *β*,*β*-difluoro peroxide [39,40,41]. The investigation of various solvents, including ethyl acetate (EA), dichloromethane (DCM), methanol, *N*,*N*-dimethylformamide (DMF), and dimethyl sulfoxide (DMSO), was carried out (entries 7–11, respectively), and the results indicated that the case of DMSO afforded **3a** an 82% yield (entry 11). In comparison, the yield of **3a** was almost not affected at 60 °C (entry 12).

**Figure 2:**
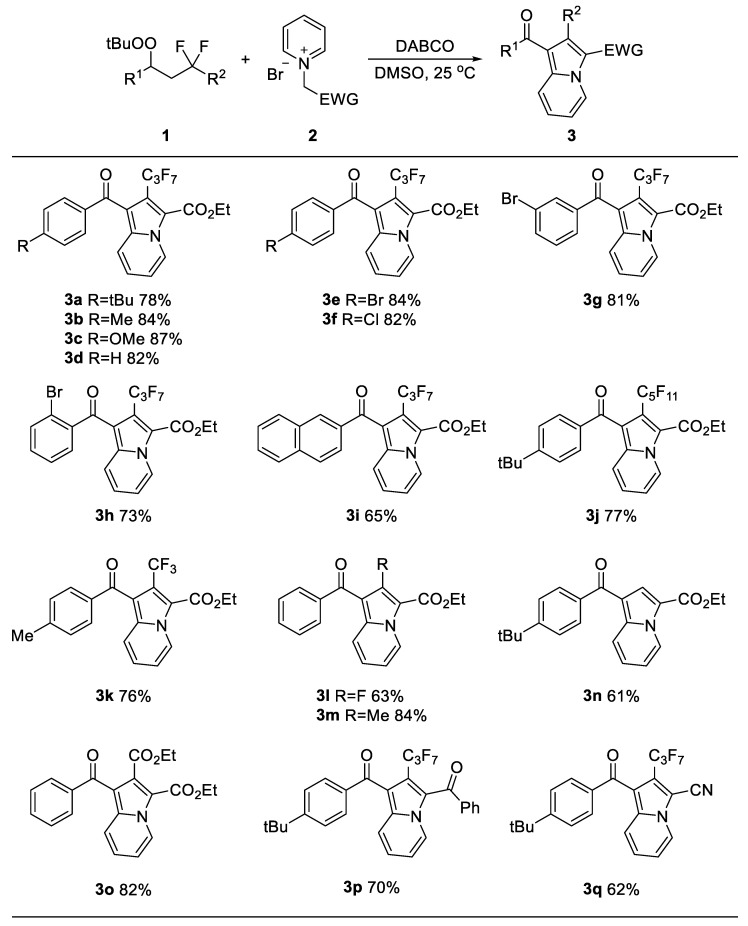
Scope of the substrates [a]. [a] Reaction conditions: **1** (0.2 mmol), **2** (0.4 mmol), DABCO (0.6 mmol), 1,4-dioxane (2.0 mL), 60 °C, 6 h, unless otherwise noted. Reported yields were the isolated yields.

With the optimized conditions established, the scope of the substrates **1** and **2** were investigated (Figure 2). Representative results are summarized in Figure 2; β,β-difluoro peroxides **1**, bearing a variety of functional groups (R = aryl), including both the electron-donating (tBu, Me, OMe) and electron-withdrawing (Cl, Br) groups, reacted smoothly with 1-(2-ethoxy-2-oxoethyl) pyridinium bromide **2**. The substituents, regardless of the *ortho-*, *meta-* or *para-* position, were all tolerated to give the corresponding 2-perfluoro-substituted indolizines **3a**–**3h** in good yields. The substrates with naphthalene could also react and give the desired products **3i** in a 65% yield. Notably, other perfluoroalkyl groups, such as C_5_F_11_ (**3j**) and CF_3_ (**3k**), could also be successfully incorporated into the indolizine skeleton when the corresponding β-perfluoroalkyl peroxides were subjected to the standard conditions. In addition, indolizines could be adorned with other functionalities, including F (**3l**), Me (**3m**), H (**3n**), and ester (**3o**), via this [3 + 2] annulation protocol in regard to the corresponding β,β-difluorinated peroxides that were examined. Moreover, when the ester group of **2a** was replaced with other electron-withdrawing substituents, such as ketone (**2b**) or cyanomethyl (**2c**), the desired products **3p** and **3q** were obtained in 70% and 62% yield respectively.

**Scheme 2:**
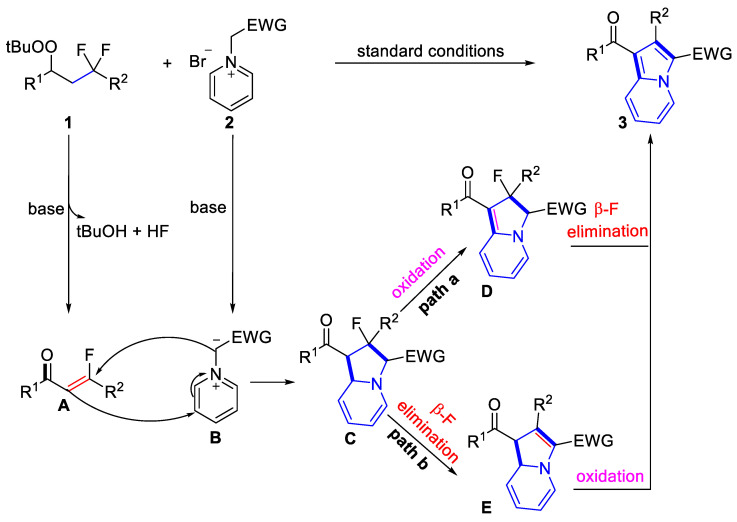
Proposed reaction mechanisms.

Based on the literature reports [35,43,44], a possible reaction mechanism is depicted in Scheme 2. The DABCO initiated Kornblum–DeLaMare rearrangement of peroxide **1** and the further elimination of one molecule of HF affords the key β- fluoro enone **A** [40]. Pyridinium ylide **B**, generated by the deprotonation of 1-(2-ethoxy-2-oxoethyl) pyridinium bromide **2**, attacks the β- fluoro enone **A**, and then the intramolecular ring-closure process occurs to form an intermediate **C**. Intermediate **C** could be oxidized to obtain intermediate **D**, which provides target product **3** via sequential β-F elimination (path a). Alternatively, intermediate **C** would undergo β-F elimination to form intermediate **E**, and finally the target product **3** could be obtained by further oxidation (path b).

## 3. Materials and Methods

### 3.1. General Information

^1^H NMR spectra were recorded on Bruker 400 MHz and 600 MHz spectrometers, and the chemical shifts were reported in parts per million (*δ*) relative to internal standard TMS (0 ppm) for CDCl_3_. The peak patterns are indicated as follows: s, singlet; d, doublet; dd, doublet of doublet; t, triplet; m, multiplet; q, quartet. The coupling constants, *J*, are reported in Hertz (Hz). ^13^C NMR spectra were obtained at Bruker 100 MHz and 150 MHz and referenced to the internal solvent signals (central peak is 77.0 ppm in CDCl_3_), and to the internal solvent signals (central peak is 39.9 ppm in DMSO). CDCl_3_ and DMSO were used as the NMR solvent. APEX II (Bruker Inc., Karlsruhe, Germany) was used for ESI-MS and EI-MS. IR spectra were recorded by a Bruker Tensor 27 infrared spectrometer. Flash column chromatography was performed over silica gel 200–300. All reagents were weighed and handled in air at room temperature. All chemical reagents were purchased from Alfa (Shanghai, China), Acros (Shanghai, China), Aldrich (Shanghai, China), TCI (Shanghai, China), Energy (Shanghai, China), and J&K (Guangzhou, China) and used without further purification.

### 3.2. General Procedures for Synthesis of β,β-Difluoro Peroxides ***1***

To a dry Schlenk tube were added tBuOOH (T-hydro, 70% in water, 6.0 mmol, 6.0 equiv), alkene (1.0 mmol, 1.0 equiv), Co(acac)_2_ (0.1 mmol, 0.1 equiv), BrCX_2_R^1^ or IC_n_F_2n+1_ (2.0 mmol, 2.0 equiv), and anhydrous MeCN (4.0 mL) under N_2_ atmosphere at room temperature. Subsequently, NEt_3_ (5.0 mmol, 5.0 equiv) was added to the mixture, and the resulting solution was stirred at ambient temperature for 5 h. The resulting mixture and the solvent were evaporated under vacuum. The residue was purified by flash column chromatography on silica gel (eluent: ethyl acetate/petroleum ether, 1:200–1:25) to give the peroxides **1a**–**1k** (Figure S1) [45,46].

To a mixture of alkene (1.0 mmol), acid (3.0 mmol), Na_2_CO_3_ (0.1 mmol) and FeCl_2_ (7.5 mg, 0.1 mmol), MeCN (10.0 mL) was added under nitrogen at room temperature. Then tert-butyl hydroperoxide (TBHP, 0.8 mmol, 5–6 M in decane) was added into the mixture under nitrogen at room temperature. The resulting mixture was stirred under rt, 390 nm, 10 W for 24 h. The resulting reaction solution was directly filtered through a pad of silica by chloroform. The solvent was evaporated in vacuo to give the crude products **1l** (Figure S1) and **1m** (Figure S1) [47].

To a dry sealed tube were added alkene (1.0 mmol), CF_2_HSO_2_Na (2.0 mmol), *^t^*BuOOH (5.5 M in decane, 5.0 mmol), CuI (0.15 mmol), and MeCN (10.0 mL), and the resulting solution was stirred at 25 °C under N_2_ atmosphere for 3 h. Then the resulting mixture was evaporated under vacuum. The residue was purified by flash column chromatography on silica gel (eluent: petroleum ether/dichloromethane) to give the pure product **1n** (Figure S1) [48].

### 3.3. General Procedures for Synthesis of 1,2,3-Trifunctionalized Indolizines ***3***

To a dry Schlenk tube were added **1** (0.1 mmol), **2** (0.2 mmol), DABCO (0.5 mmol), and DMSO (1.0 mL) at room temperature and the resulting solution was stirred at 25 °C for 5 h. After the mixture was cooled to room temperature, EtOAc (20.0 mL) was added and the mixture was washed with H_2_O (5.0 mL each) 3 times. The combined organic layer was dried over Na_2_SO_4_, filtrated, and concentrated under reduced pressure. The residue was purified by flash column chromatography on silica gel (petroleum ether/ethyl acetate) to give the indolizines **3**.

*Ethyl 1-(4-(tert-butyl)benzoyl)-2-(3,3,3,3,3,3,3-heptafluoro-3λ^8^-prop-1-yn-1-yl)indolizine-3-carboxylate* **3a**. (40 mg, 78%). Yellow solid, mp 136–138 °C. Isolated by flash column chromatography (petroleum ether: ethyl acetate = 10:1, R*f* = 0.4); ^1^H NMR (600 MHz, CDCl_3_, ppm) *δ* 9.50 (d, *J* = 7.5 Hz, 1H), 7.75 (d, *J* = 8.6 Hz, 2H), 7.22–7.20 (m, 1H), 7.08–7.03 (m, 1H), 6.98–6.94 (m, 1H), 4.44 (q, *J* = 14.4 Hz, 7.0 Hz, 2H), 1.40 (t, *J* = 7.2 Hz, 3H), 1.34 (s, 9H); ^13^C NMR (150 MHz, CDCl_3_) *δ* 191.3, 160.7, 157.6, 135.6, 134.5, 129.8, 127.4, 125.6, 123.8, 119.2 (t, *J* = 27.8), 118.7, 116.2 (t, *J* = 4.1), 115.2, 113.8, 61.3, 35.2, 31.1, 13.8, not all carbons are reported due to extensive ^19^F splitting; ^19^F NMR (564 MHz, CDCl_3_) *δ* −80.2 (t, *J* = 10.2, 3F), −97.7 to −97.8 (m, 2F), −121.9 to −122.0 (m, 2F); HRMS (ESI) calcd for C_25_H_23_F_7_NO_3_ [M + H^+^]: 518.1561; found: 518.1551.

*Ethyl 2-(3,3,3,3,3,3,3-heptafluoro-3λ^8^-prop-1-yn-1-yl)-1-(4-methylbenzoyl)indolizine-3-carboxylate* **3b**. (40 mg, 84%). Yellow solid, mp 125–126 °C. Isolated by flash column chromatography (petroleum ether: ethyl acetate = 10:1, R*f* = 0.4); ^1^H NMR (600 MHz, CDCl_3_, ppm) *δ* 9.50 (d, *J* = 7.3 Hz, 1H), 7.71 (d, *J* = 8.3 Hz, 2H), 7.23 (d, *J* = 8.0 Hz, 2H), 7.19 (d, *J* = 8.9 Hz, 1H), 7.06–7.03 (m, 1H), 6.97–6.94 (m, 1H), 4.44 (q, *J* = 14.4 Hz, 7.1 Hz, 2H), 2.41 (s, 3H), 1.40 (t, *J* = 7.1 Hz, 3H); ^13^C NMR (150 MHz, CDCl_3_) *δ* 191.3, 160.7, 144.7, 135.8, 134.6, 130.0, 129.3, 127.4, 123.7, 119.1 (t, *J* = 28.5), 118.6, 116.1 (t, *J* = 4.0), 115.2, 113.8, 61.3, 21.7, 13.7, not all carbons are reported due to extensive ^19^F splitting; ^19^F NMR (564 MHz, CDCl_3_) *δ* −80.2 (t, *J* = 10.7, 3F), −97.8 to −97.7 (m, 2F), −121.9 to −122.0 (m, 2F); HRMS (ESI) calcd for C_22_H_17_F_7_NO_3_ [M + H^+^]: 476.1091; found: 476.1083.

*Ethyl 2-(3,3,3,3,3,3,3-heptafluoro-3λ^8^-prop-1-yn-1-yl)-1-(4-methoxybenzoyl)indolizine-3-carboxylate* **3c**. (43 mg, 87%). Yellow solid, mp 93–96 °C. Isolated by flash column chromatography (petroleum ether: ethyl acetate = 10:1, R*f* = 0.4); ^1^H NMR (600 MHz, CDCl_3_, ppm) *δ* 9.49 (d, *J* = 7.3 Hz, 1H), 7.79 (d, *J* = 8.7 Hz, 2H), 7.21 (d, *J* = 9.0 Hz, 1H), 7.07–7.03 (m, 1H), 6.98–6.94 (m, 1H), 6.91 (d, *J* = 8.9 Hz, 2H), 4.44 (q, *J* = 14.3 Hz, 7.1 Hz, 2H), 3.87 (s, 3H), 1.40 (t, *J* = 7.1 Hz, 3H); ^13^C NMR (150 MHz, CDCl_3_) *δ* 190.2, 164.1, 160.7, 134.4, 132.2, 131.3, 127.4, 123.7, 119.0 (t, *J* = 28.9), 118.6, 116.3 (t, *J* = 4.2), 115.2, 113.8, 113.7, 61.3, 55.5, 13.8, not all carbons are reported due to extensive ^19^F splitting; ^19^F NMR (564 MHz, CDCl_3_) *δ* −80.2 (t, *J* = 10.3, 3F), −97.9 to −98.0 (m, 2F), −121.9 to −122.0 (m, 2F); HRMS (ESI) calcd for C_22_H_17_F_7_NO_4_ [M + H^+^]: 492.1040; found: 492.1026.

*Ethyl 1-benzoyl-2-(3,3,3,3,3,3,3-heptafluoro-3λ^8^-prop-1-yn-1-yl)indolizine-3-carboxylate* **3d**. (38 mg, 82%). Yellow solid, mp 98–100 °C. Isolated by flash column chromatography (petroleum ether: ethyl acetate = 10:1, R*f* = 0.4); ^1^H NMR (600 MHz, CDCl_3_, ppm) *δ* 9.49 (d, *J* = 7.3 Hz, 1H), 7.81 (d, *J* = 7.8 Hz, 2H), 7.59 (t, *J* = 7.5 Hz, 1H), 7.44 (t, *J* = 7.6 Hz, 2H), 7.19 (d, *J* = 8.8 Hz, 1H), 7.06 (t, *J* = 6.8 Hz, 1H), 6.96 (t, *J* = 6.9 Hz, 1H), 4.44 (q, *J* = 14.2 Hz, 7.1 Hz, 2H), 1.40 (t, *J* = 7.1 Hz, 3H); ^13^C NMR (150 MHz, CDCl_3_) *δ* 191.7, 160.6, 138.3, 134.7, 133.6, 129.8, 128.6, 127.4, 123.9, 119.3 (t, *J* = 28.0), 118.6, 115.8 (t, *J* = 4.1), 115.3, 113.9, 61.4, 13.8, not all carbons are reported due to extensive ^19^F splitting; ^19^F NMR (564 MHz, CDCl_3_) *δ* −80.2 (t, *J* = 10.6, 3F), −97.6 to −97.7 (m, 2F), −121.8 to −121.9 (m, 2F); HRMS (ESI) calcd for C_21_H_15_F_7_NO_3_ [M + H^+^]: 462.0935; found: 462.0921.

*Ethyl 1-(4-bromobenzoyl)-2-(3,3,3,3,3,3,3-heptafluoro-3λ^8^-prop-1-yn-1-yl)indolizine-3-carboxylate* **3e**. (45 mg, 84%). Yellow solid, mp 96–98 °C. Isolated by flash column chromatography (petroleum ether: ethyl acetate = 10:1, R*f* = 0.4); ^1^H NMR (600 MHz, CDCl_3_, ppm) *δ* 9.48 (d, *J* = 7.3 Hz, 1H), 7.68 (d, *J* = 8.5 Hz, 2H), 7.58 (d, *J* = 8.5 Hz, 2H), 7.19 (d, *J* = 8.9 Hz, 1H), 7.09 (t, *J* = 6.8 Hz, 1H), 6.98 (t, *J* = 7.2 Hz, 1H), 4.44 (q, *J* = 14.4 Hz, 7.2 Hz, 2H), 1.40 (t, *J* = 7.2 Hz, 3H); ^13^C NMR (150 MHz, CDCl_3_) *δ* 190.6, 160.6, 137.1, 134.7, 134.7, 132.0, 131.2, 129.0, 127.5, 124.2, 119.2 (t, *J* = 27.8), 118.4, 115.4, 115.1 (t, *J* = 4.0), 114.1, 61.5, 13.8, not all carbons are reported due to extensive ^19^F splitting; ^19^F NMR (564 MHz, CDCl_3_) *δ* −80.2 (t, *J* = 10.3, 3F), −97.7 to −97.7 (m, 2F), −121.9 to −122.0 (m, 2F); HRMS (ESI) calcd for C_21_H_14_F_7_BrNO_3_ [M + H^+^]: 540.0040; found: 540.0028.

*Ethyl 1-(4-chlorobenzoyl)-2-(3,3,3,3,3,3,3-heptafluoro-3λ^8^-prop-1-yn-1-yl)indolizine-3-carboxylate* **3f**. (40 mg, 82%). Yellow solid, mp 110–113 °C. Isolated by flash column chromatography (petroleum ether: ethyl acetate = 10:1, R*f* = 0.4); ^1^H NMR (600 MHz, CDCl_3_, ppm) *δ* 9.40 (d, *J* = 7.3 Hz, 1H), 7.69 (d, *J* = 8.7 Hz, 2H), 7.33 (d, *J* = 8.7 Hz, 2H), 7.12–7.10 (m, 1H), 7.03–6.99 (m, 1H), 6.92–6.88 (m, 1H), 4.36 (q, *J* = 14.4 Hz, 7.1 Hz, 2H), 1.32 (t, *J* = 7.1 Hz, 3H); ^13^C NMR (150 MHz, CDCl_3_) *δ* 190.4, 160.6, 140.2, 136.7, 134.6, 131.1, 129.0, 127.5, 124.2, 119.2 (t, *J* = 27.7), 118.4, 115.4, 115.2 (t, *J* = 4.1), 114.1, 61.5, 13.7, not all carbons are reported due to extensive ^19^F splitting; ^19^F NMR (564 MHz, CDCl_3_) *δ* −80.2 (t, *J* = 10.4, 3F), −97.8 to −97.6 (m, 2F), −121.9 to −122.0 (m, 2F); HRMS (ESI) calcd for C_21_H_14_F_7_ClNO_3_ [M + H^+^]: 496.0545; found: 496.0536.

*Ethyl 1-(3-bromobenzoyl)-2-(3,3,3,3,3,3,3-heptafluoro-3λ^8^-prop-1-yn-1-yl)indolizine-3-carboxylate* **3g**. (44 mg, 81%). Yellow solid, mp 90–91 °C. Isolated by flash column chromatography (petroleum ether: ethyl acetate = 10:1, R*f* = 0.4); ^1^H NMR (600 MHz, CDCl_3_, ppm) *δ* 9.49 (d, *J* = 7.4 Hz, 1H), 7.97 (s, 1H), 7.71 (t, *J* = 6.8 Hz, 2H), 7.32 (t, *J* = 7.8 Hz, 1H), 7.20 (d, *J* = 9.1 Hz, 1H), 7.11 (t, *J* = 6.8 Hz, 1H), 7.00 (t, *J* = 6.8 Hz, 1H), 4.44 (q, *J* = 14.2 Hz, 7.1 Hz, 2H), 1.40 (t, *J* = 7.1 Hz, 3H); ^13^C NMR (150 MHz, CDCl_3_) *δ* 190.3, 160.5, 140.2, 136.4, 134.8, 132.4, 130.2, 128.4, 127.5, 124.4, 123.0, 119.3 (t, *J* = 27.1), 118.4, 115.4, 114.9 (t, *J* = 4.0), 114.2, 61.5, 13.8, not all carbons are reported due to extensive ^19^F splitting; ^19^F NMR (564 MHz, CDCl_3_) *δ* −80.2 (t, *J* = 10.7, 3F), −97.6 to −97.4 (m, 2F), −121.9 to −122.0 (m, 2F); HRMS (ESI) calcd for C_21_H_14_F_7_BrNO_3_ [M + H^+^]: 540.0040; found: 540.0036.

*Ethyl 1-(2-bromobenzoyl)-2-(3,3,3,3,3,3,3-heptafluoro-3λ^8^-prop-1-yn-1-yl)indolizine-3-carboxylate* **3h**. (39 mg, 73%). Yellow oil. Isolated by flash column chromatography (petroleum ether: ethyl acetate = 10:1, R*f* = 0.4); ^1^H NMR (600 MHz, CDCl_3_, ppm) *δ* 9.18–9.16 (m, 1H), 7.58–7.56 (m, 1H), 7.40–7.38 (m, 1H), 7.30–7.24 (m, 2H), 7.07–7.00 (m, 2H), 6.90–6.86 (m, 1H), 4.38 (q, *J* = 14.4 Hz, 7.1 Hz, 2H), 1.32 (t, *J* = 7.1 Hz, 3H); ^13^C NMR (150 MHz, CDCl_3_) *δ* 189.9, 160.8, 141.3, 136.6, 134.1, 132.4, 131.0, 127.4, 127.3, 125.3, 120.7, 119.7 (t, *J* = 28.5), 118.7, 116.1, 115.5, 115.4, 61.8, 13.7, not all carbons are reported due to extensive ^19^F splitting; ^19^F NMR (564 MHz, CDCl_3_) *δ* −80.3 (t, *J* = 10.1, 3F), −96.4 to −96.5 (m, 2F), −120.9 to −121.0 (m, 2F); HRMS (ESI) calcd for C_21_H_14_F_7_BrNO_3_ [M + H^+^]: 540.0040; found: 540.0034.

*Ethyl 1-(2-naphthoyl)-2-(3,3,3,3,3,3,3-heptafluoro-3λ^8^-prop-1-yn-1-yl)indolizine-3-carboxylate* **3i**. (33 mg, 65%). Yellow solid, mp 138–142 °C. Isolated by flash column chromatography (petroleum ether: ethyl acetate = 10:1, R*f* = 0.4); ^1^H NMR (600 MHz, CDCl_3_, ppm) *δ* 9.51 (d, *J* = 7.3 Hz, 1H), 8.20 (s, 2H), 8.02 (d, *J* = 8.7 Hz, 1H), 7.92–7.82 (m, 3H), 7.60 (t, *J* = 7.2 Hz, 1H), 7.51 (t, *J* = 7.6 Hz, 1H), 7.03 (t, *J* = 6.9 Hz, 1H), 6.97 (t, *J* = 7.1 Hz, 1H), 4.46 (q, *J* = 14.3 Hz, 7.1 Hz, 2H), 1.41 (t, *J* = 7.2 Hz, 3H); ^13^C NMR (150 MHz, CDCl_3_) *δ* 191.6, 160.7, 135.9, 135.7, 134.8, 132.6, 132.5, 129.7, 128.9, 128.6, 127.9, 127.5, 126.9, 124.6, 124.0, 119.4 (t, *J* = 28.4 Hz), 118.6, 116.0 (t, *J* = 4.1 Hz), 115.3, 114.0, 61.4, 13.8, not all carbons are reported due to extensive ^19^F splitting; ^19^F NMR (564 MHz, CDCl_3_) *δ* −80.2 (t, *J* = 10.4, 3F), −97.6 to −97.7 (m, 2F), −121.8 to −121.9 (m, 2F); HRMS (ESI) calcd for C_25_H_17_F_7_NO_3_ [M + H^+^]: 512.1091; found: 512.1085.

*Ethyl 1-(4-(tert-butyl)benzoyl)-2-(5,5,5,5,5,5,5,5,5,5,5-undecafluoro-5λ^12^-penta-1,3-diyn-1-yl)indolizine-3-carboxylate* **3j**. (47 mg, 77%). Yellow solid, mp 118–120 °C. Isolated by flash column chromatography (petroleum ether: ethyl acetate = 10:1, R*f* = 0.4); ^1^H NMR (600 MHz, CDCl_3_, ppm) *δ* 9.50 (d, *J* = 7.3 Hz, 1H), 7.74 (d, *J* = 8.5 Hz, 2H), 7.44 (d, *J* = 8.5 Hz, 2H), 7.21 (d, *J* = 8.9 Hz, 1H), 7.07–7.04 (m, 1H), 6.96 (td, *J* = 7.3 Hz, 1.2 Hz, 1H), 4.44 (q, *J* = 14.3 Hz, 7.3 Hz, 2H), 1.40 (t, 3H), 1.34 (s, 9H); ^13^C NMR (150 MHz, CDCl_3_) *δ* 191.3, 160.7, 157.6, 135.6, 134.5, 129.8, 127.4, 125.6, 123.7, 119.3 (t, *J* = 28.0), 118.7, 116.3 (t, *J* = 4.3), 115.2, 113.8, 61.3, 35.2, 31.1, 13.7, not all carbons are reported due to extensive ^19^F splitting; ^19^F NMR (564 MHz, CDCl_3_) *δ* −80.8 (t, *J* = 10.7, 3F), −97.2 to −97.3 (m, 2F), −117.9 (m, 2F), −122.6 to −122.7 (m, 2F), −126.0 to −126.1 (m, 2F); HRMS (ESI) calcd for C_27_H_23_F_11_NO_3_ [M + H^+^]: 618.1497; found: 618.1488.

*Ethyl 1-(4-methylbenzoyl)-2-(trifluoromethyl)indolizine-3-carboxylate* **3k**. (29 mg, 76%). White solid, mp 92–94 °C. Isolated by flash column chromatography (petroleum ether: ethyl acetate = 10:1, R*f* = 0.4); ^1^H NMR (600 MHz, CDCl_3_, ppm) *δ* 9.54–9.51 (m, 1H), 7.71 (d, *J* = 8.2 Hz, 2H), 7.57–7.54 (m, 1H), 7.27–7.24 (m, 2H), 7.18–7.14 (m, 1H), 7.03–6.99 (m, 1H), 4.45 (q, *J* = 14.4 Hz, 7.3 Hz, 2H), 2.42 (s, 3H), 1.43 (t, *J* = 7.3 Hz, 3H); ^13^C NMR (150 MHz, CDCl_3_) *δ* 191.1, 160.4, 144.4, 136.3, 135.3, 129.7, 129.3, 127.3, 124.6, 122.3 (q, *J* = 270.3 Hz), 122.2 (q, *J* = 37.0 Hz), 119.0, 115.7, 114.2 (d, *J* = 2.2Hz), 112.4(d, *J* = 3.1Hz), 61.3, 21.7, 13.9; ^19^F NMR (564 MHz, CDCl_3_) *δ* −52.6 (s, 3F); HRMS (ESI) calcd for C_20_H_17_F_3_NO_3_ [M + H^+^]: 376.1155; found: 376.1145.

*Ethyl 1-benzoyl-2-fluoroindolizine-3-carboxylate* **3l** [6,35]. (20 mg, 63%). Yellow solid, mp 118–120 °C. Isolated by flash column chromatography (petroleum ether: ethyl acetate = 10:1, R*f* = 0.4); ^1^H NMR (600 MHz, CDCl_3_, ppm) *δ* 9.57 (d, *J* = 7.0 Hz, 1H), 8.43 (d, *J* = 8.9 Hz, 1H), 7.83–7.80 (m, 2H), 7.59–7.55 (m, 1H), 7.51–7.44 (m, 3H), 7.13–7.09 (m, 1H), 4.41 (q, *J* = 14.3 Hz, 7.1 Hz, 2H), 1.39 (t, *J* = 7.1 Hz, 3H).

*Ethyl 1-benzoyl-2-methylindolizine-3-carboxylate* **3m**. (26 mg, 84%). Yellow solid, mp 96–98 °C. Isolated by flash column chromatography (petroleum ether: ethyl acetate = 10:1, R*f* = 0.4); ^1^H NMR (600 MHz, CDCl_3_, ppm) *δ* 9.60 (d, *J* = 7.1 Hz, 1H), 7.74–7.72 (m, 2H), 7.65 (d, *J* = 9.1 Hz, 1H), 7.58–7.54 (m, 1H), 7.46 (t, *J* = 7.9 Hz, 2H), 7.18–7.14 (m, 1H), 6.92–6.89 (m, 1H), 4.42 (q, *J* = 14.2 Hz, 7.1 Hz, 2H), 2.47 (s, 3H), 1.43 (t, *J* = 7.1 Hz, 3H); ^13^C NMR (150 MHz, CDCl_3_) *δ* 192.6, 162.4, 141.0, 138.9, 136.5, 132.0, 129.2, 128.5, 128.0, 125.5, 118.6, 114.7, 114.0, 60.2, 14.5, 14.4; HRMS (ESI) calcd for C_19_H_18_NO_3_ [M + H^+^]: 308.1281; found: 308.1273.

*Ethyl 1-benzoylindolizine-3-carboxylate* **3n**. (21 mg, 61%). Yellow solid, mp 89–90 °C. Isolated by flash column chromatography (petroleum ether: ethyl acetate = 10:1, R*f* = 0.4); ^1^H NMR (600 MHz, CDCl_3_, ppm) *δ* 9.58 (d, *J* = 7.0 Hz, 1H), 8.64 (d, *J* = 8.9 Hz, 1H), 7.85 (s, 1H), 7.81 (d, *J* = 8.2 Hz, 2H), 7.53 (d, *J* = 8.2 Hz, 2H), 7.42 (t, *J* = 7.8 Hz, 1H), 7.08 (t, *J* = 6.9 Hz, 1H), 1.42–1.39 (m, 12H); ^13^C NMR (150 MHz, CDCl_3_) *δ* 191.3, 1161.3, 155.0, 140.1, 137.4, 129.0, 127.9, 126.9, 126.1, 125.3, 120.7, 115.5, 114.7, 113.0, 60.4, 35.0, 31.3, 14.6; HRMS (ESI) calcd for C_22_H_24_NO_3_ [M + H^+^]: 350.1751; found: 350.1738.

*Diethyl 1-benzoylindolizine-2,3-dicarboxylate* **3o**. (30 mg, 82%). White solid, mp 89–91 °C. Isolated by flash column chromatography (petroleum ether: ethyl acetate = 10:1, R*f* = 0.4); ^1^H NMR (600 MHz, CDCl_3_, ppm) *δ* 9.57 (d, *J* = 7.1 Hz, 1H), 7.91 (d, *J* = 9.1 Hz, 1H), 7.73–7.71 (m, 2H), 7.57–7.53 (m, 1H), 7.45 (d, *J* = 7.7 Hz, 2H), 7.33–7.29 (m, 1H), 7.07–7.03 (m, 1H), 4.37 (q, *J* = 14.4 Hz, 7.2 Hz, 2H), 3.95 (q, *J* = 14.4 Hz, 7.2 Hz, 2H), 1.35 (t, *J* = 7.2 Hz, 3H), 1.19 (t, *J* = 7.2 Hz, 3H); ^13^C NMR (150 MHz, CDCl_3_) *δ* 190.7, 165.3, 160.4, 140.0, 138.0, 131.9, 130.4, 128.8, 128.2, 127.9, 126.8, 119.9, 115.7, 112.7, 112.5, 61.7, 61.0, 14.1, 13.8; HRMS (ESI) calcd for C_21_H_20_NO_5_ [M + H^+^]: 366.1336; found: 366.1323.

*(3-Benzoyl-2-(3,3,3,3,3,3,3-heptafluoro-3λ^8^-prop-1-yn-1-yl)indolizin-1-yl)(4-(tert-butyl)phenyl)methanone* **3p**. (38 mg, 70%). Yellow solid, mp 69–70 °C. Isolated by flash column chromatography (petroleum ether: ethyl acetate = 10:1, R*f* = 0.4); ^1^H NMR (600 MHz, CDCl_3_, ppm) *δ* 7.92 (d, *J* = 7.2 Hz, 1H), 7.83 (d, *J* = 7.2 Hz, 2H), 7.79 (d, *J* = 8.4 Hz, 2H), 7.65 (t, *J* = 7.4 Hz, 1H), 7.52–7.48 (m, 4H), 7.15 (d, *J* = 9.3 Hz, 1H), 6.95–6.91 (m, 1H), 6.73–6.69 (m, 1H), 1.37 (s, 9H); ^13^C NMR (150 MHz, CDCl_3_) *δ* 190.5, 188.5, 157.2, 137.7, 136.3, 134.6, 134.4, 129.8, 129.7, 128.9, 125.6, 124.9, 123.6, 123.5, 122.9, 119.6, 114.4, 113.8, 35.2, 31.1, not all carbons are reported due to extensive ^19^F splitting; ^19^F NMR (564 MHz, CDCl_3_) *δ* −80.1 (t, *J* = 10.3, 3F), −100.0 to −100.1 (m, 2F), −123.5 to −123.6 (m, 2F); HRMS (ESI) calcd for C_29_H_23_F_7_NO_2_ [M + H^+^]: 550.1612; found: 550.1603.

*1-(4-(tert-butyl)benzoyl)-2-(3,3,3,3,3,3,3-heptafluoro-3λ^8^-prop-1-yn-1-yl)indolizine-3-carbonitrile* **3q**. (29 mg, 62%). Yellow solid, mp 135–138 °C. Isolated by flash column chromatography (petroleum ether: ethyl acetate = 10:1, R*f* = 0.4); ^1^H NMR (600 MHz, CDCl_3_, ppm) *δ* 8.42 (d, *J* = 6.9 Hz, 1H), 7.72 (d, *J* = 8.4 Hz, 2H), 7.47 (d, *J* = 8.4 Hz, 2H), 7.25 (d, *J* = 7.8 Hz, 1H), 7.17 (t, *J* = 6.9 Hz, 1H), 7.10 (t, *J* = 6.8 Hz, 1H), 1.36 (s, 9H); ^13^C NMR (150 MHz, CDCl_3_) *δ* 188.8, 157.9, 135.6, 135.5, 129.8, 125.7, 125.3, 123.1, 122.8, 122.5, 120.0, 116.2, 115.1, 110.7, 35.3, 31.1, not all carbons are reported due to extensive ^19^F splitting; ^19^F NMR (564 MHz, CDCl_3_) *δ* −79.9 (t, *J* = 9.9, 3F), −103.9 to −104.0 (m, 2F), −124.2 to −124.3 (m, 2F); HRMS (ESI) calcd for C_23_H_18_F_7_N_2_O [M + H^+^]: 471.1302; found: 471.1288.

## 4. Conclusions

In conclusion, we have developed a simple and efficient method for the synthesis of indolizines by base-mediated [3 + 2] annulation of β,β-difluoro peroxides as C2-Building Blocks and pyridinium ylides. With this protocol, a series of 1,2,3-trifunctionalized indolizines were afforded in moderate to good yields under ambient conditions. The operational simplicity and good functional group compatibility would render this protocol as a useful tool in organic synthesis.

## Figures and Tables

**Table 1:** Optimization studies ^a^.

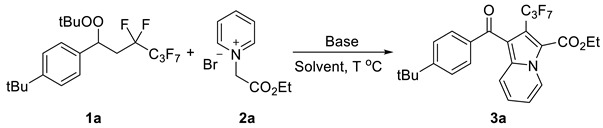				
**Entry**	**Base**	**Solvent**	**T (°C)**	**Yield (%) ^b^**
1	DABCO	MeCN	25	54
2	DIPEA	MeCN	25	N.R.
3	NEt_3_	MeCN	25	trace
4	K_3_PO_4_	MeCN	25	N.R.
5	CsCO_3_	MeCN	25	trace
6	KOH	MeCN	25	N.R.
7	DABCO	EA	25	11
8	DABCO	DCM	25	13
9	DABCO	MeOH	25	trace
10	DABCO	DMF	25	59
11	DABCO	DMSO	25	82
12	DABCO	DMSO	60	83

^a^ Reaction conditions: **1a** (0.1 mmol), **2a** (0.2 mmol), base (0.5 mmol), solvent (1.0 mL), 5 h, under air. ^b^ Reported yields were based on **1a** and determined by ^1^H NMR using CH_2_Br_2_ as an internal standard.

## Data Availability

Data will be made available upon request.

## Supplementary Materials

The following supporting information can be downloaded at: https://www.mdpi.com/article/10.3390/molecules29245927/s1, S1: Figure S1. β,β-difluoro peroxides **1** and 1-(2-ethoxy-2-oxoethyl)pyridinium bromide **2**; S2: Copies of ^1^H and ^13^C NMR spectra for **3**.

## References

[1] Johnson B.M., Shu Y.-Z., Zhuo X., Meanwell N.A. (2020). Metabolic and Pharmaceutical Aspects of Fluorinated Compounds. J. Med. Chem..

[2] Prchalová E., Stepánek O., Smrcek S., Kotora M. (2014). Medicinal Applications of Perfluoroalkylated Chain-containing Compounds. Future Med. Chem..

[3] Han J., Remete A.M., Dobson L.S., Kiss L., Izawa K., Moriwaki H., Soloshonok V.A., O’Hagan D. (2020). Next Generation Organofluorine Containing Blockbuster Drugs. J. Fluor. Chem..

[4] Meanwell N.A. (2018). Fluorine and Fluorinated Motifs in the Design and Application of Bioisosteres for Drug Design. J. Med. Chem..

[5] Kirk K.L. (2008). Fluorination in Medicinal Chemistry: Methods, Strategies, and Recent Developments. Org. Process Res. Dev..

[6] Inoue M., Sumii Y., Shibata N. (2020). Contribution of Organofluorine Compounds to Pharmaceuticals. ACS Omega.

[7] Tu H.-Y., Wang F., Huo L., Li Y., Zhu S., Zhao X., Li H., Qing F.-L., Chu L. (2020). Enantioselective Three-Component Fluoroalkylarylation of Unactivated Olefins through Nickel-Catalyzed Cross-Electrophile Coupling. J. Am. Chem. Soc..

[8] Tong C.-L., Xu X.-H., Qing F.-L. (2019). Oxidative Hydro-, Bromo-, and Chloroheptafluoroisopropylation of Unactivated Alkenes with Heptafluoroisopropyl Silver. Org. Lett..

[9] Ni C., Hu J. (2016). The Unique Fluorine Effects in Organic Reactions: Recent Facts and Insights into Fluoroalkylations. Chem. Soc. Rev..

[10] Zhao X., Tu H.-Y., Guo L., Zhu S., Qing F.-L., Chu L. (2018). Intermolecular Selective Carboacylation of Alkenes via Nickel-catalyzed Reductive Radical Relay. Nat. Commun..

[11] Fu M.-L., Liu J.-B., Xu X.-H., Qing F.-L. (2017). Synthesis of Pentafluoroethyl Ethers by Silver-Mediated Oxidative Pentafluoroethylation of Alcohols and Phenols. J. Org. Chem..

[12] Ni C., Hu M., Hu J. (2015). Good Partnership between Sulfur and Fluorine: Sulfur-Based Fluorination and Fluoroalkylation Reagents for Organic Synthesis. Chem. Rev..

[13] Nagib D.A., MacMillan D.W.C. (2011). Trifluoromethylation of Arenes and Heteroarenes by Means of Photoredox Catalysis. Nature.

[14] Fujiwara Y., Dixon J.A., O’Hara F., Funder E.D., Dixon D.D., Rodriguez R.A., Baxter R.D., Herle B., Sach N., Collins M.R. (2012). Practical and Innate Carbon-Hydrogen Functionalization of Heterocycles. Nature.

[15] Barata-Vallejo S., Cooke M.V., Postigo A. (2018). Radical Fluoroalkylation Reactions. ACS Catal..

[16] Cheng B., Ge D., Wang X., Chu X. (2021). Perfluoroalkyl Halides as Fluorine-Containing Building Blocks for the Synthesis of Fluoroalkylated Heterocycles. Chin. J. Org. Chem..

[17] Meador W.E., Autry S.A., Bessetti R.N., Gayton J.N., Flynt A.S., Hammer N.I., Delcamp J.H. (2020). Water-Soluble NIR Absorbing and Emitting Indolizine Cyanine and Indolizine Squaraine Dyes for Biological Imaging. J. Org. Chem..

[18] Guidotti B.B., da Silva T.S., Correia J.T.M., Coelho F. (2020). Brønsted-acid-catalyzed selective Friedel-Crafts monoalkylation of Isatins with Indolizines in Water. Org. Biomol. Chem..

[19] Correia J.T.M., List B., Coelho F. (2017). Catalytic Asymmetric Conjugate Addition of Indolizines to *α*,*β*-Unsaturated Ketones. Angew. Chem. Int. Ed..

[20] Yang L., Pu X., Niu D., Fu Z., Zhang X. (2019). Copper-Catalyzed Asymmetric Propargylation of Indolizines. Org. Lett..

[21] Gundersen L.-L., Negussie A.H., Rise F., Østby O.B. (2003). Antimycobacterial Activity of 1-Substituted Indolizines. Arch. Pharm..

[22] Hazra A., Mondal S., Maity A., Naskar S., Saha P., Paira R., Sahu K.B., Paira P., Ghosh S., Sinha C. (2011). Amberlite-IRA-402 (OH) ion Exchange Resin Mediated Synthesis of Indolizines, Pyrrolo [1,2-a] Quinolines and Isoquinolines: Antibacterial and Antifungal Evaluation of the Products. Eur. J. Med. Chem..

[23] Bloch W.M., Derwent-Smith S.M., Issa F., Morris J.C., Rendina L.M., Sumby C.J. (2011). Fused Pyrazino[2,3-b]indolizine and Indolizino[2,3-b]quinoxaline Derivatives; Synthesis, Structures, and Properties. Tetrahedron.

[24] Dawood K.M., Abdel-Gawad H., Ellithey M., Mohamed H.A., Hegazi B. (2006). Synthesis, Anticonvulsant, and Anti-inflammatory Activities of Some New Benzofuran-based Heterocycles. Arch. Pharm..

[25] Østby O.B., Dalhus B., Gundersen L.-L., Rise F., Bast A., Haenen G.R.M.M. (2000). Synthesis of 1-Substituted 7-Cyano-2,3-diphenylindolizines and Evaluation of Antioxidant Properties. Eur. J. Org. Chem..

[26] Sadowski B., Klajn J., Gryko D.T. (2016). Recent Advances in the Synthesis of Indolizines and Their π-Expanded Analogues. Org. Biomol. Chem..

[27] Yadagiri D., Rivas M., Gevorgyan V. (2020). Denitrogenative Transformations of Pyridotriazoles and Related Compounds: Synthesis of N-Containing Heterocyclic Compounds and Beyond. J. Org. Chem..

[28] Zhang X., Zhang J., Liu Z., Bi W., Shen J., Li G. (2024). Efficient Solvent-Free Synthesis of Indolizines Using CuBr Catalyst from Pyridine, Acetophenone, and Electron-Deficient Alkenes. Molecules.

[29] He D., Xu Y.-H., Han J., Deng H.-M., Shao M., Chen J., Zhang H., Cao W.-G. (2017). Facile One-pot Tandem Synthesis of Perfluoroalkylated Indolizines under Metal-Free Mild Conditions. Tetrahedron.

[30] Shen B.-X., Li B., Wang B.-Q. (2016). Rh(III)-Catalyzed Oxidative Annulation Leading to Substituted Indolizines by Cleavage of C(sp^2^)-H/C(sp^3^)-H Bonds. Org. Lett..

[31] Brioche J., Meyer C., Cossy J. (2015). Synthesis of 2-Aminoindolizines by 1,3-Dipolar Cycloaddition of Pyridinium Ylides with Electron-Deficient Ynamides. Org. Lett..

[32] Asghari S., Qandalee M., Behboodi V., Gorji A.N., Pasha G.F. (2016). Chemoselective Synthesis of Novel Aminoindolizines Using Aminopyridines, Acetylenic Diesters and *α*-Halo Ketones. Chin. Chem. Lett..

[33] Hu H.-Y., Feng J.-J., Zhu Y.-L., Gu N., Kan Y.-H. (2012). Copper Acetate Monohydrate: A Cheap but Efficient Oxidant for Synthesizing Multi-Substituted Indolizines from Pyridinium Ylides and Electron Deficient Alkenes. RSC Adv..

[34] Bakshi D., Singh A. (2016). Transition-Metal-Free Synthesis of Nitrogen Containing Heterocycles with Fully Substituted N-fused Pyrrole Rings. Asian J. Org. Chem..

[35] Shu S., Wang L., Lv L., Li Z. (2023). Copper-Catalyzed [3+2] Annulation of Pyridinium Ylides with α-CF_3_ Ketones: Synthesis of Functionalized 2-Fluoroindolizines. Asian J. Org. Chem..

[36] Bonneau R., Ramoshin Y.N., Liu M.T.H., MacPherson S.E. (1994). Synthesis of 3-Substituted Indolizines from the Reaction of Chlorocarbenes with 2-Vinylpyridine. J. Chem. Soc. Chem. Commun..

[37] Barluenga J., Lonzi G., Riesgo L., LóPez L.A., TomáS M. (2010). Pyridine Activation via Copper(I)-Catalyzed Annulation toward Indolizines. J. Am. Chem. Soc..

[38] Nugent R.A., Murphy M. (1987). The Synthesis of Indolizines: The Reaction of .alpha.-halo Pyridinium Salts with .beta.-Dicarbonyl Species. J. Org. Chem..

[39] Ma Y., Chen Y., Lou C., Li Z. (2020). DABCO-Mediated [4+1] Cycloaddition of β,β-Dihalo Peroxides with Sodium Azide toward Isoxazoles. Asian J. Org. Chem..

[40] Ma Y., Lv L., Li Z. (2022). *β*-Perfluoroalkyl Peroxides as Fluorinated C3-Building Blocks for the Construction of Benzo[4,5]imidazo[1,2-a]pyridines. J. Org. Chem..

[41] Ma Y., Chen Y., Lv L., Li Z. (2021). Regioselective Synthesis of Emission Color-Tunable Pyrazolo[1,5-a]pyrimidines with *β*,*β*-Difluoro Peroxides as 1,3-Bis-Electrophiles. Adv. Synth. Catal..

[42] The *p*Ka Data Can Be Obtained Free of Charge. https://organicchemistrydata.org/hansreich/resources/pka/#pka_-water_compilation_evans.

[43] Yang L.-M., Zhang Y.-Y., Deng J.-T., Ma A.-J., Zhang X.-Z., Zhang S.-Y., Peng J.-B. (2021). Oxidative [3+2] Annulation of Pyridinium Salts with *gem*-Difluoroalkenes: Synthesis of 2-Fluoroindolizines. Asian J. Org. Chem..

[44] Zhang J.-Q., Hu D., Song J., Ren H. (2021). [3 + 2]-Annulation of *gem*-Difluoroalkenes and Pyridinium Ylides: Access to Functionalized 2-Fluoroindolizines. J. Org. Chem..

[45] Shi E., Liu J., Liu C., Shao Y., Wang H., Lv Y., Ji M., Bao X., Wan X. (2016). Difunctionalization of Styrenes with Perfluoroalkyl and tertButylperoxy Radicals: Room Temperature Synthesis of (1-(tertbutylperoxy)-2-perfluoroalkyl)-ethylbenzene. J. Org. Chem..

[46] Chen Y., Li L., Ma Y., Li Z. (2019). Cobalt-Catalyzed Three-Component Difluoroalkylation-Peroxidation of Alkenes. J. Org. Chem..

[47] Huang Q., Lou C., Lv L., Li Z. (2024). Photoinduced Fluoroalkylation-Peroxidation of Alkenes Enabled by Ligand-to-Iron Charge Transfer Mediated Decarboxylation. Chem. Commun..

[48] Liu F., Lv L., Ma Y., Li Z. (2022). Copper-Catalyzed Radical Difluoromethylation-Peroxidation of Alkenes: Synthesis of *β*-Difluoromethyl Peroxides. Asian J. Org. Chem..

