# Supplementary material for: β,β-Difluoro Peroxides as Fluorinated C2-Building Blocks for the Construction of Functionalized Indolizines
Source: Molecules. 2024 Dec 16;29(24):5927. doi: 10.3390/molecules29245927 (PMC11676711; doi:10.3390/molecules29245927)
Supplement: Supplementary file 1 [file molecules-29-05927-s001.zip › molecules-3354920-supplementary.pdf]

Supplementary Materials

β,β-Difluoro Peroxides as Fluorine-Containing C2-Building Blocks for the Construction of Functionalized Indolizines

Yangyang Ma[a,*], Hua Zhang[b], Gangfeng Du[a], Xiaoxiao Du[a], Shurui Fang[a], Kexin Yin[a], Zhengshan Tian[a], Zhonghao Zhou[c,*]

[a] College of Chemistry and Chemical Engineering, Pingdingshan University, Pingdingshan, Henan 467000, China

[b] College of Medicine, Pingdingshan University, Pingdingshan, Henan 467000, China

[c] School of Material Science and Engineering, Dalian Jiaotong University, Dalian 116028, China.

Correspondence: mayang66789@163.com (Y. M.); zhonghao_zhou@alu.ruc.edu.cn (Z. Z.)

Table of Contents

S1. Starting materials

S2. Copies of ^{1}H, ^{13}C and ^{19}F NMR spectra for **3**

S1. Starting materials

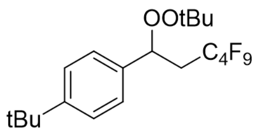

1a

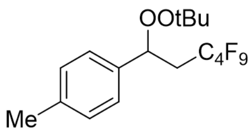

1b

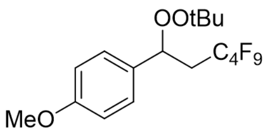

1c

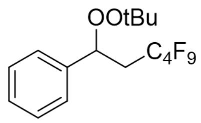

1d

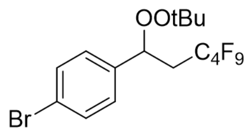

1e

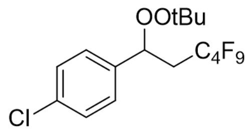

1f

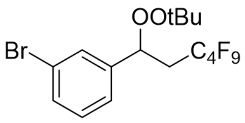

1g

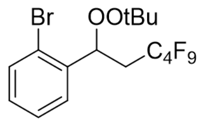

1h

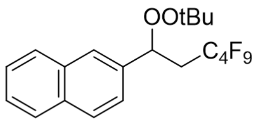

1i

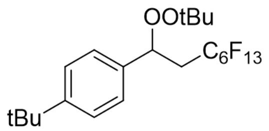

1j

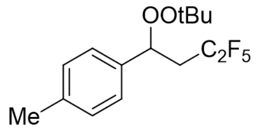

1k

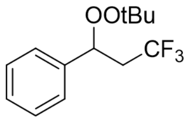

1l

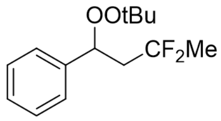

1m

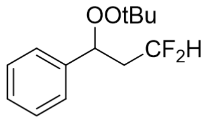

1n

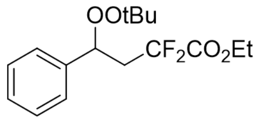

1o

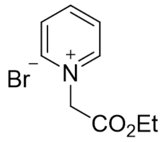

2a

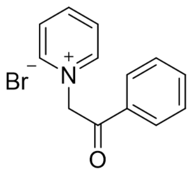

2b

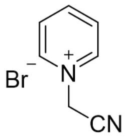

2c

Figure S1. β,β-difluoro peroxides **1** and 1-(2-ethoxy-2-oxoethyl)pyridinium bromide **2**

S2. Copies of ^{1}H and ^{13}C NMR spectra for 3

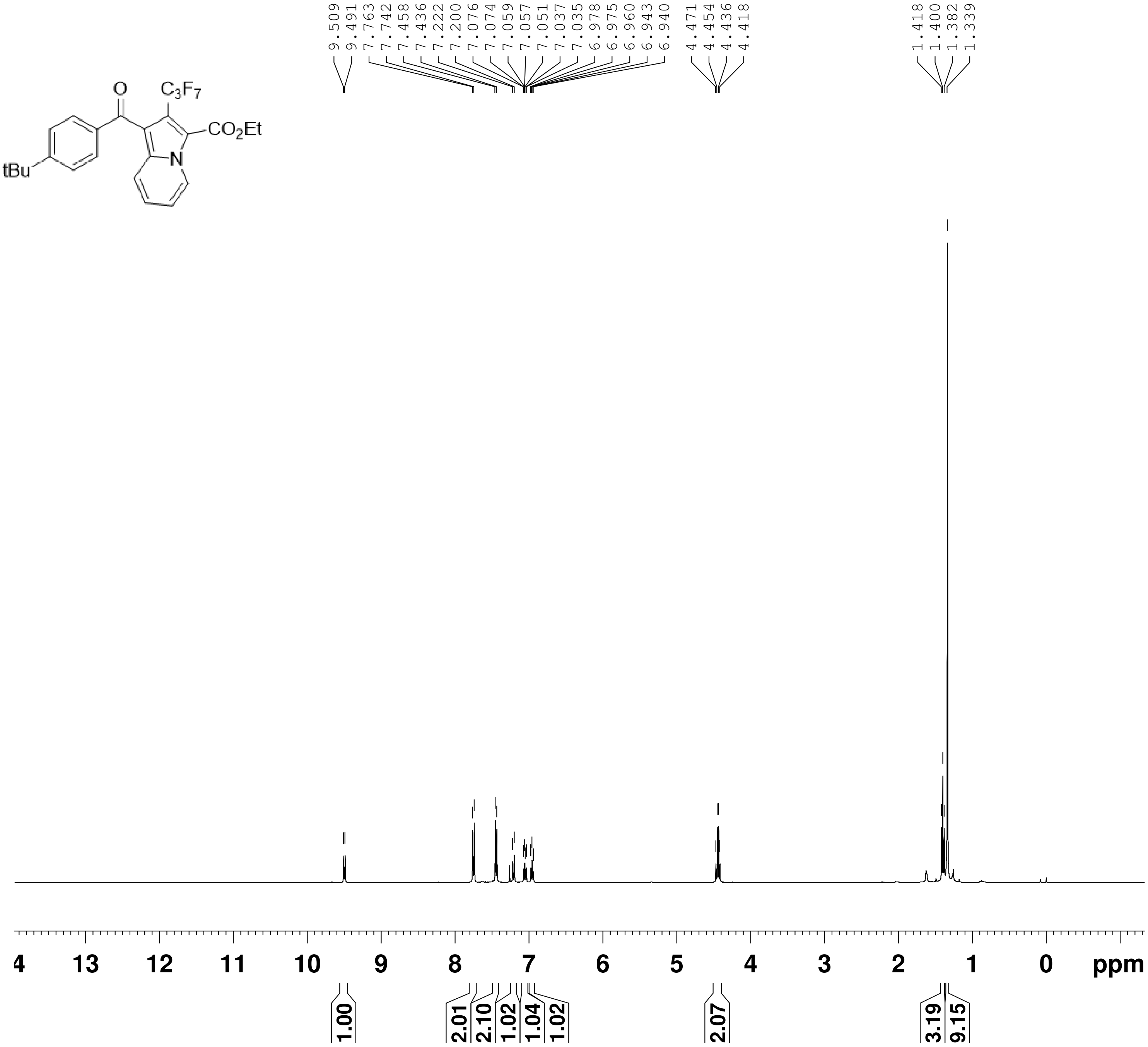

NAME	LV-MM-41-1
EXPNO	33
PROCNO	1
Date_	20240812
Time	1.41 h
INSTRUM	Avance
PROBHD	Z163739_0744 (
PULPROG	zg30
TD	65536
SOLVENT	CDCl3
NS	8
DS	0
SWH	6250.000 Hz
FIDRES	0.190735 Hz
AQ	5.2429299 sec
RG	90.5
DW	80.000 usec
DE	8.64 usec
TE	298.0 K
D1	1.00000000 sec
TD0	1
SFO1	400.1326008 MHz
NUC1	1H
P0	2.67 usec
P1	8.00 usec
SI	65536
SF	400.1300090 MHz
WDW	EM
SSB	0
LB	0.30 Hz
GB	0
PC	1.00

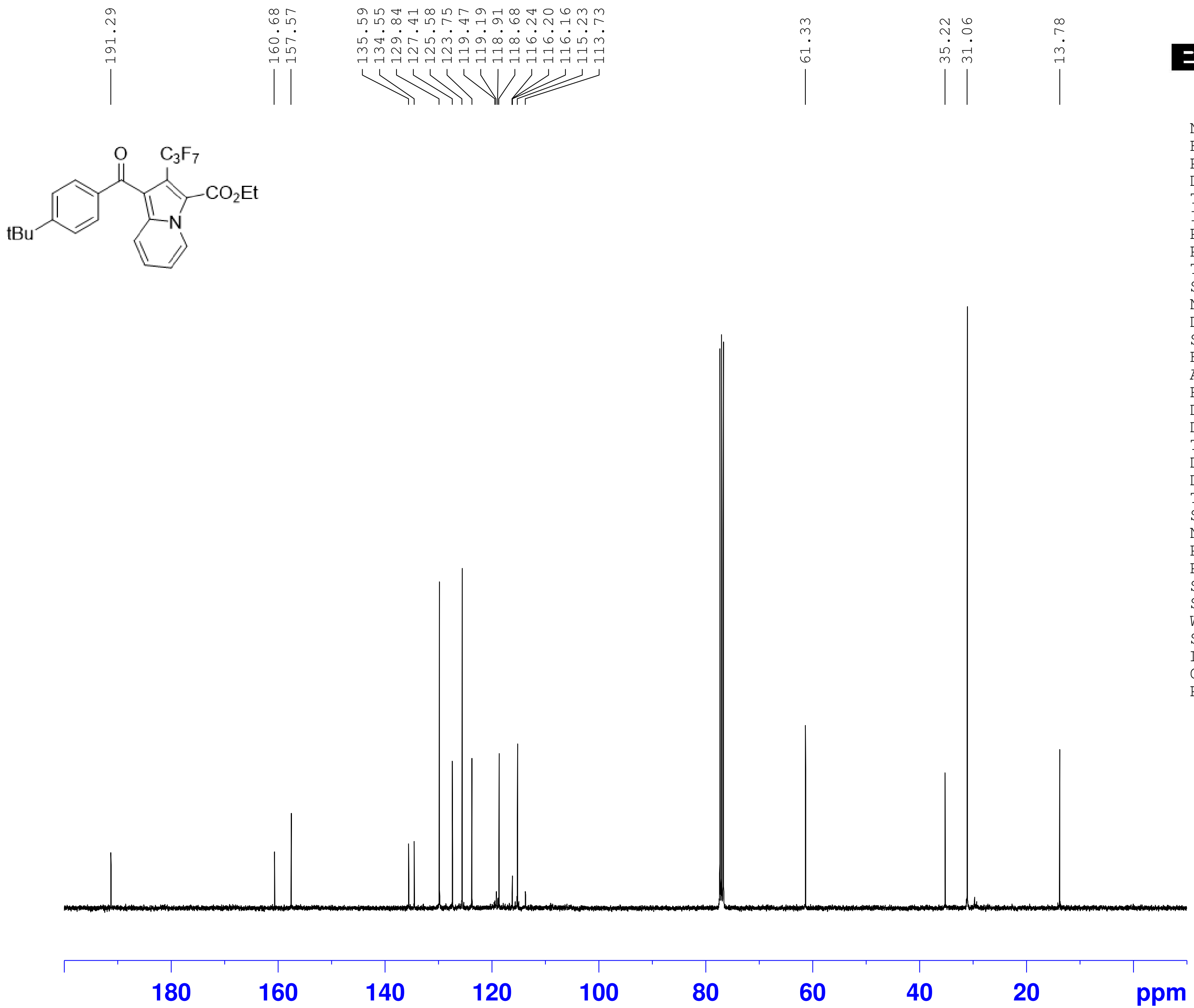

NAME	LV-MM-41-1
EXPNO	34
PROCNO	1
Date_	20240812
Time	2.42 h
INSTRUM	Avance
PROBHD	Z163739_0744 (
PULPROG	zgpg30
TD	65536
SOLVENT	CDCl3
NS	1024
DS	4
SWH	23809.523 Hz
FIDRES	0.726609 Hz
AQ	1.3763061 se
RG	101
DW	21.000 us
DE	6.50 us
TE	298.0 K
D1	2.00000000 se
D11	0.03000000 se
TD0	1
SFO1	100.6228298 MH
NUC1	13C
P0	2.67 us
P1	8.00 us
SI	32768
SF	100.6127685 MH
WDW	EM
SSB	0
LB	1.00 Hz
GB	0
PC	1.40

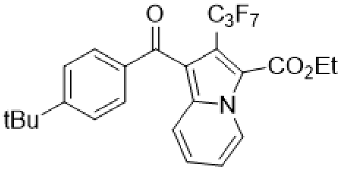

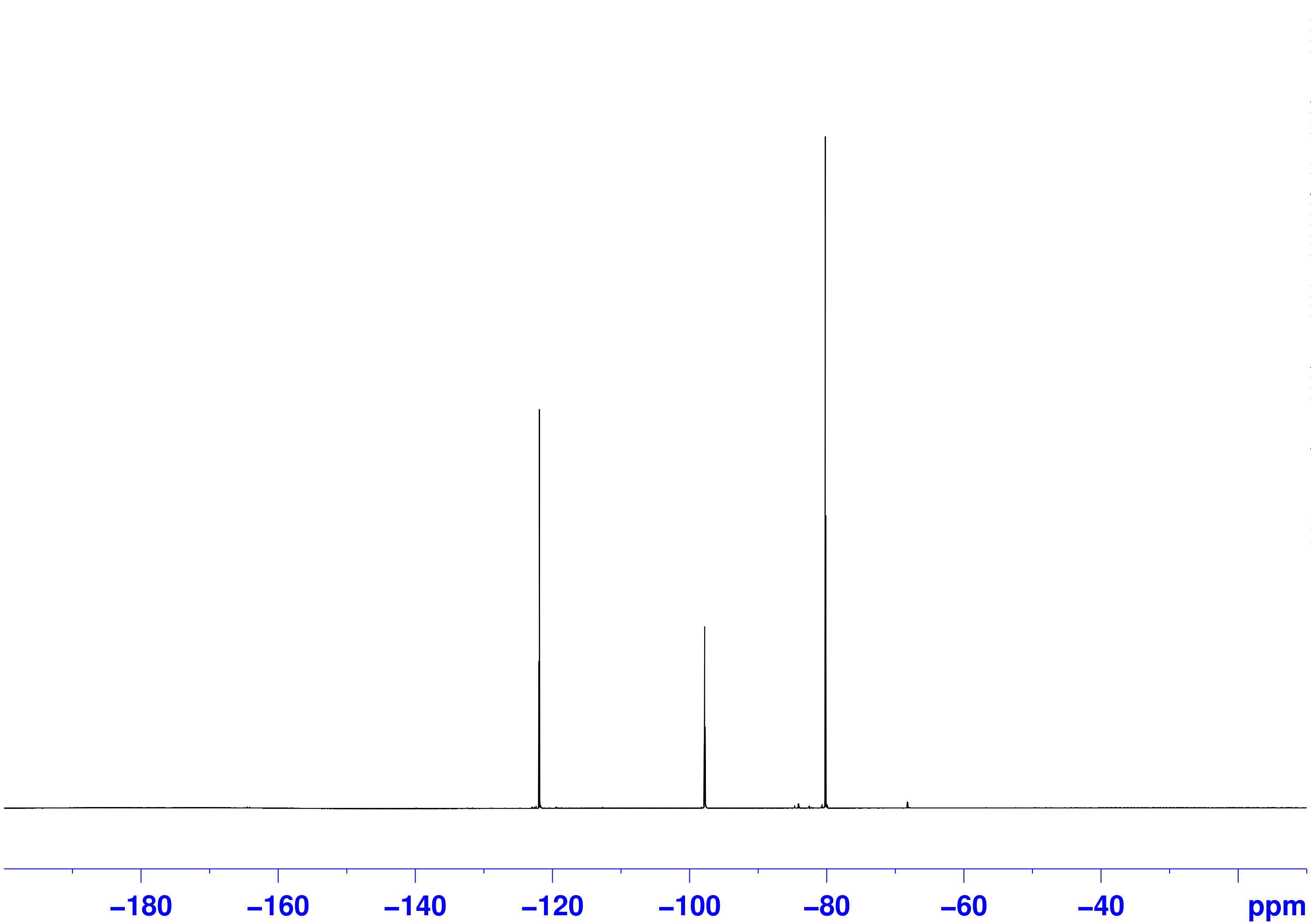

NAME	LV-MM-41-1
EXPNO	35
PROCNO	1
Date_	20240812
Time	2.44 h
INSTRUM	Avance
PROBHD	Z163739_0744 (
PULPROG	zgig
TD	131072
SOLVENT	CDCl3
NS	16
DS	4
SWH	90909.094 Hz
FIDRES	1.387163 Hz
AQ	0.7209460 se
RG	101
DW	5.500 us
DE	6.50 us
TE	298.1 K
D1	1.00000000 se
D11	0.03000000 se
TD0	1
SFO1	376.4607164 MH
NUC1	19F
P1	12.00 us
SI	65536
SF	376.4983662 MH
WDW	EM
SSB	0
LB	0.30 Hz
GB	0
PC	1.00

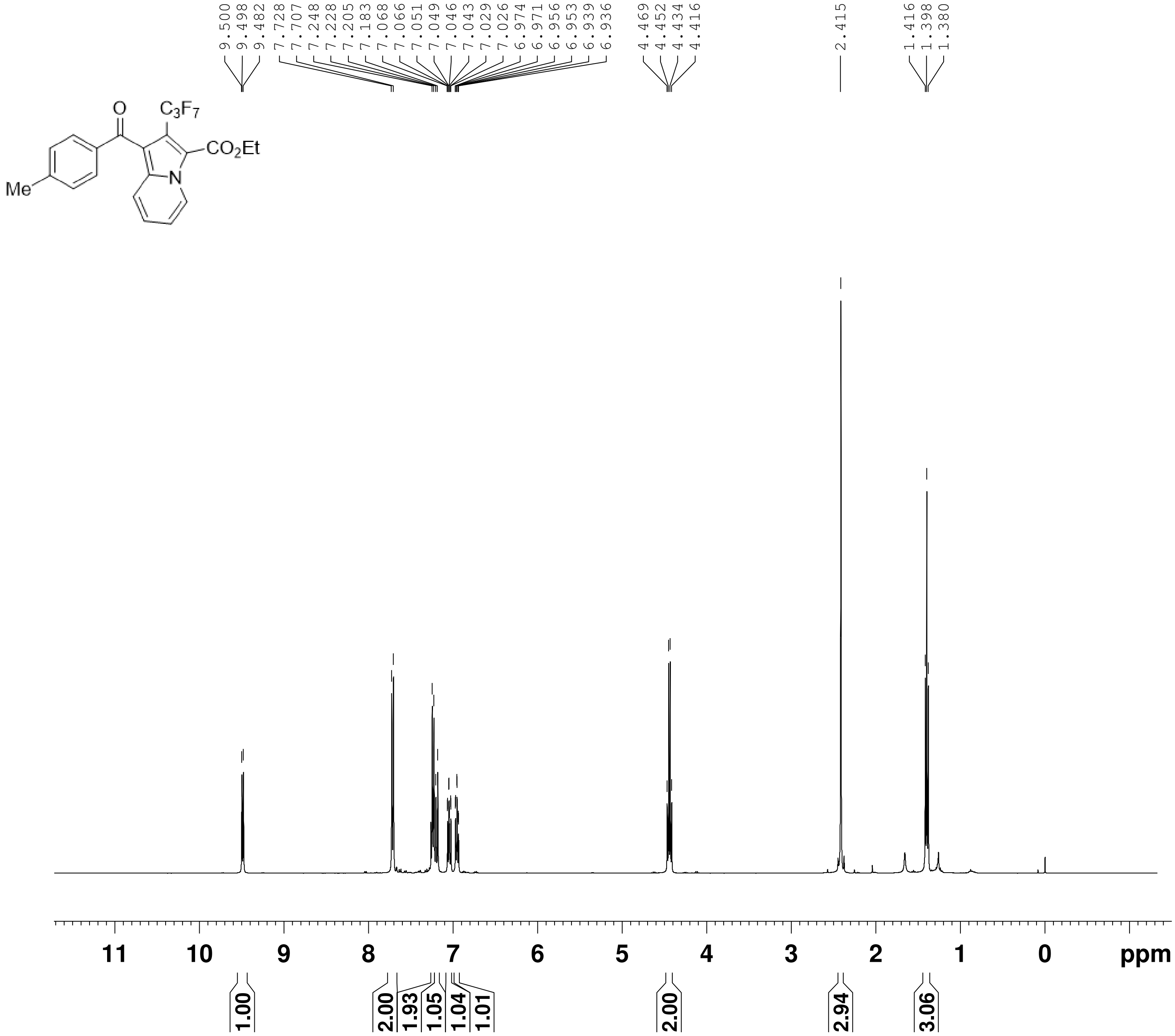

NAME	LV-MM-92-1
EXPNO	123
PROCNO	1
Date_	20240812
Time	11.53 h
INSTRUM	Avance
PROBHD	Z163739_0744 (
PULPROG	zg30
TD	65536
SOLVENT	CDCl3
NS	8
DS	0
SWH	6250.000 Hz
FIDRES	0.190735 Hz
AQ	5.2429299 sec
RG	57
DW	80.000 usec
DE	8.64 usec
TE	298.0 K
D1	1.00000000 sec
TD0	1
SFO1	400.1326008 MHz
NUC1	1H
P0	2.67 usec
P1	8.00 usec
SI	65536
SF	400.1300087 MHz
WDW	EM
SSB	0
LB	0.30 Hz
GB	0
PC	1.00

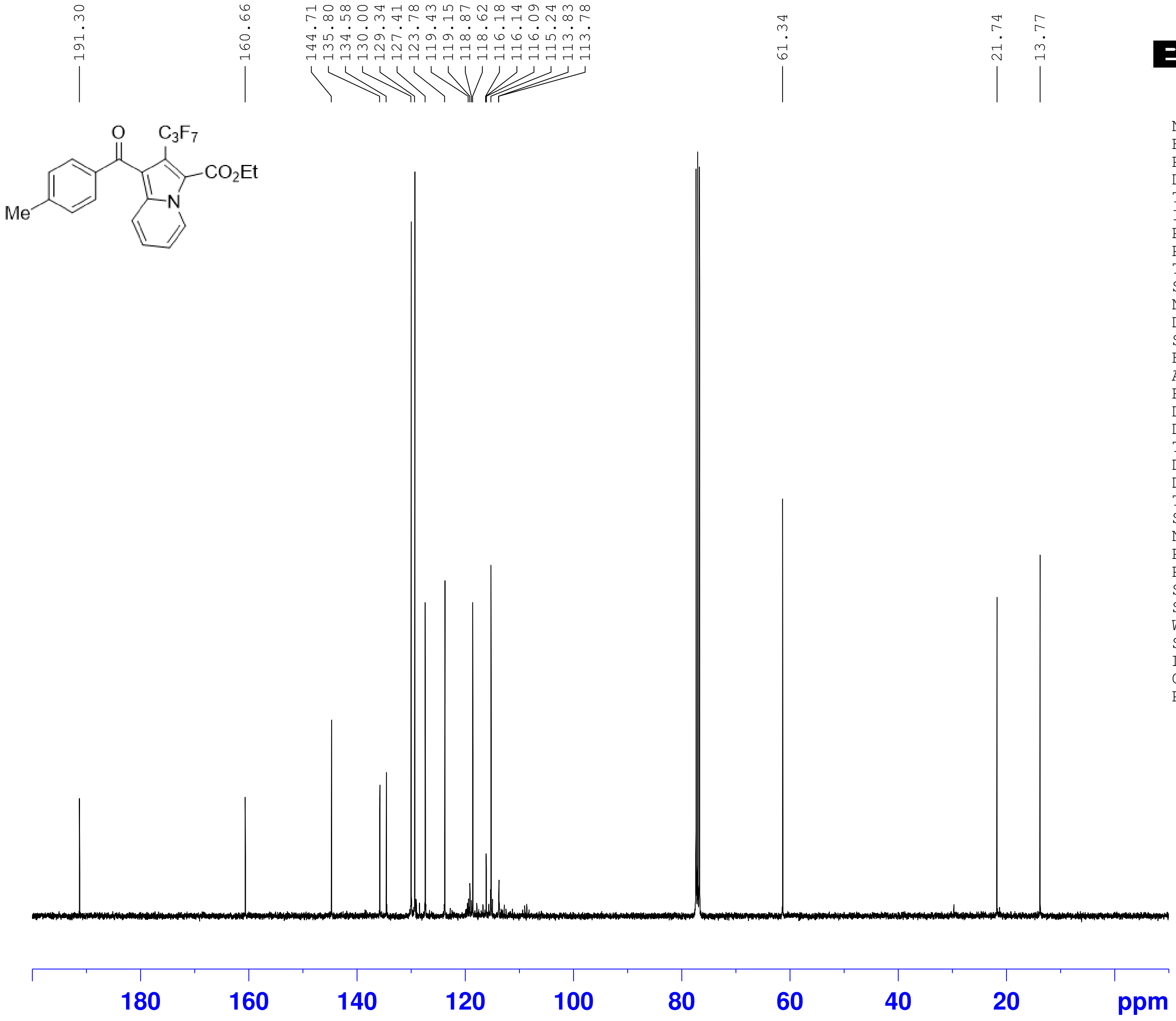

BRUKER

NAME	LV-MM-92-1
EXPNO	124
PROCNO	1
Date_	20240812
Time	12.54 h
INSTRUM	Avance
PROBHD	Z163739_0744 (
PULPROG	zgpg30
TD	65536
SOLVENT	CDCl3
NS	1024
DS	4
SWH	23809.523 Hz
FIDRES	0.726609 Hz
AQ	1.3763061 se
RG	101
DW	21.000 us
DE	6.50 us
TE	298.0 K
D1	2.00000000 se
D11	0.03000000 se
TD0	1
SFO1	100.6228298 MH
NUC1	13C
P0	2.67 us
P1	8.00 us
SI	32768
SF	100.6127685 MH
WDW	EM
SSB	0
LB	1.00 Hz
GB	0
PC	1.40

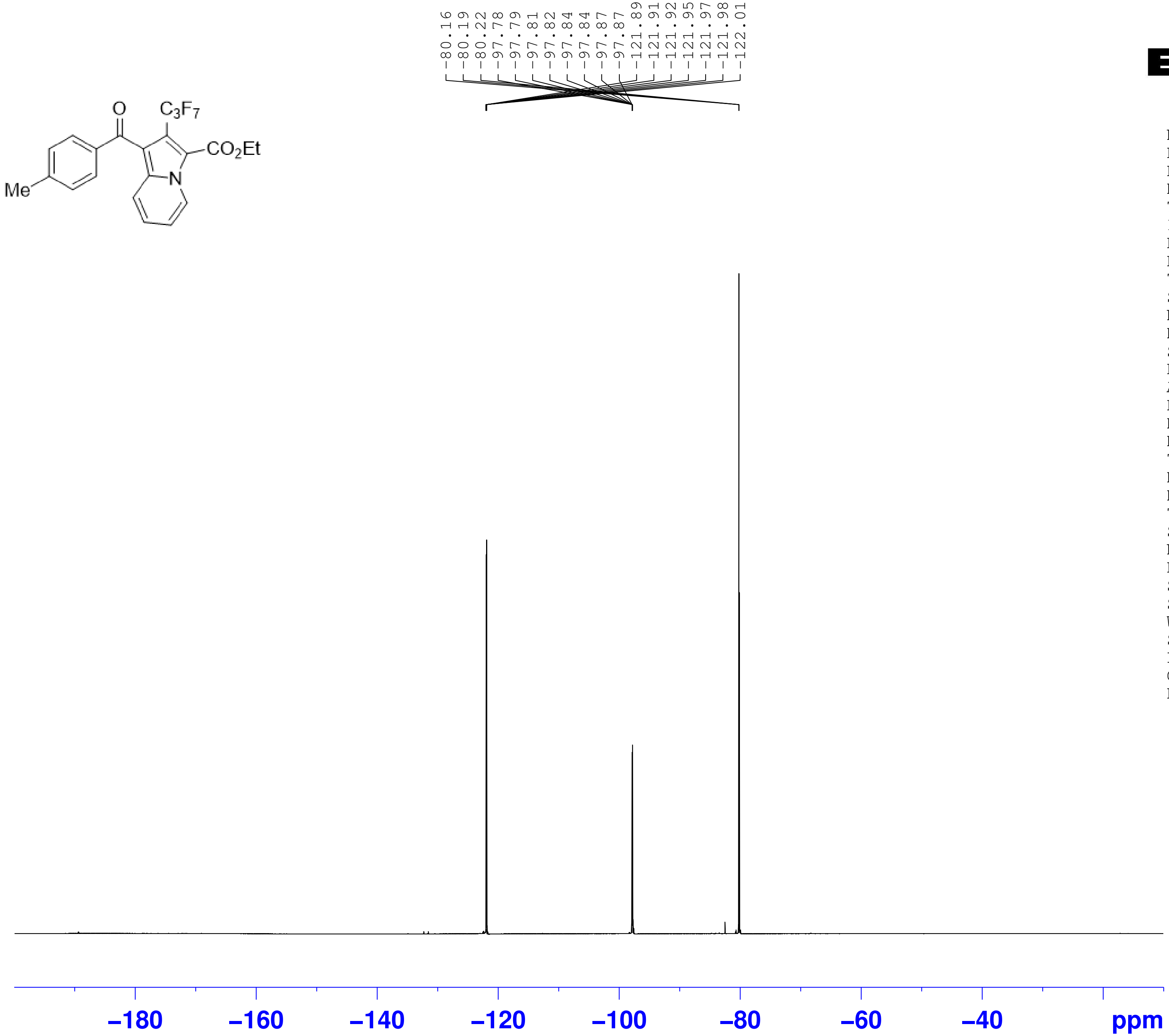

NAME	LV-MM-92-1
EXPNO	125
PROCNO	1
Date_	20240812
Time	12.56 h
INSTRUM	Avance
PROBHD	Z163739_0744 (
PULPROG	zgig
TD	131072
SOLVENT	CDCl3
NS	16
DS	4
SWH	90909.094 Hz
FIDRES	1.387163 Hz
AQ	0.7209460 se
RG	101
DW	5.500 us
DE	6.50 us
TE	298.1 K
D1	1.00000000 se
D11	0.03000000 se
TD0	1
SFO1	376.4607164 MH
NUC1	19F
P1	12.00 us
SI	65536
SF	376.4983662 MH
WDW	EM
SSB	0
LB	0.30 Hz
GB	0
PC	1.00

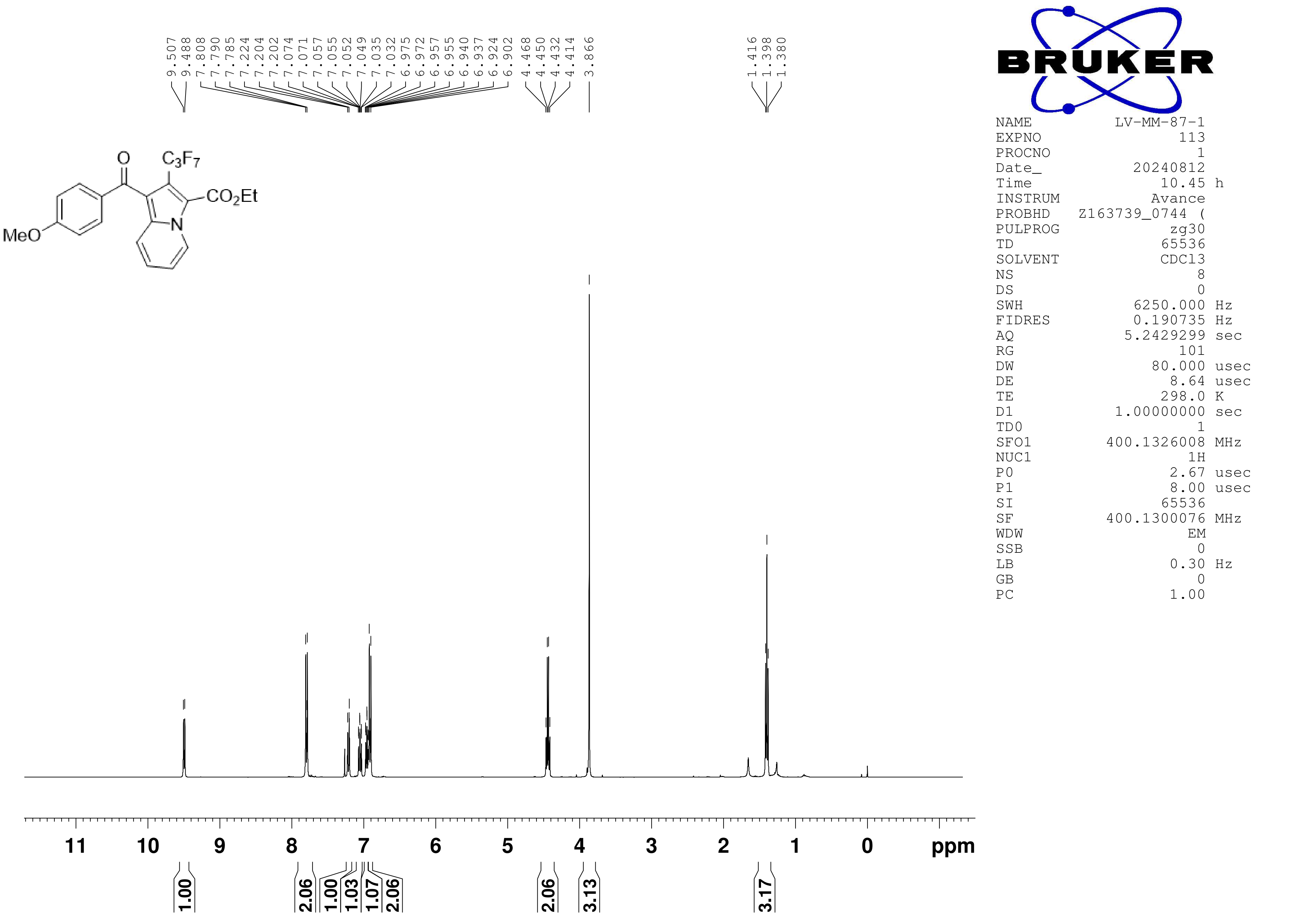
9.507
9.488
7.808
7.790
7.785
7.224
7.204
7.202
7.074
7.071
7.057
7.055
7.052
7.049
7.035
7.032
6.975
6.972
6.957
6.955
6.940
6.937
6.924
6.902
4.468
4.450
4.432
4.414
3.866
1.416
1.398
1.380
MeO
O
C_3F_7
CO_2Et
N
BRUKER
NAME LV-MM-87-1
EXPNO 113
PROCNO 1
Date_ 20240812
Time 10.45 h
INSTRUM Avance
PROBHD Z163739_0744 (
PULPROG zg30
TD 65536
SOLVENT CDCl3
NS 8
DS 0
SWH 6250.000 Hz
FIDRES 0.190735 Hz
AQ 5.2429299 sec
RG 101
DW 80.000 usec
DE 8.64 usec
TE 298.0 K
D1 1.00000000 sec
TD0 1
SFO1 400.1326008 MHz
NUC1 1H
P0 2.67 usec
P1 8.00 usec
SI 65536
SF 400.1300076 MHz
WDW EM
SSB 0
LB 0.30 Hz
GB 0
PC 1.00
11 10 9 8 7 6 5 4 3 2 1 0 ppm
1.00
2.06
1.00
1.03
1.07
2.06
2.06
3.13
3.17

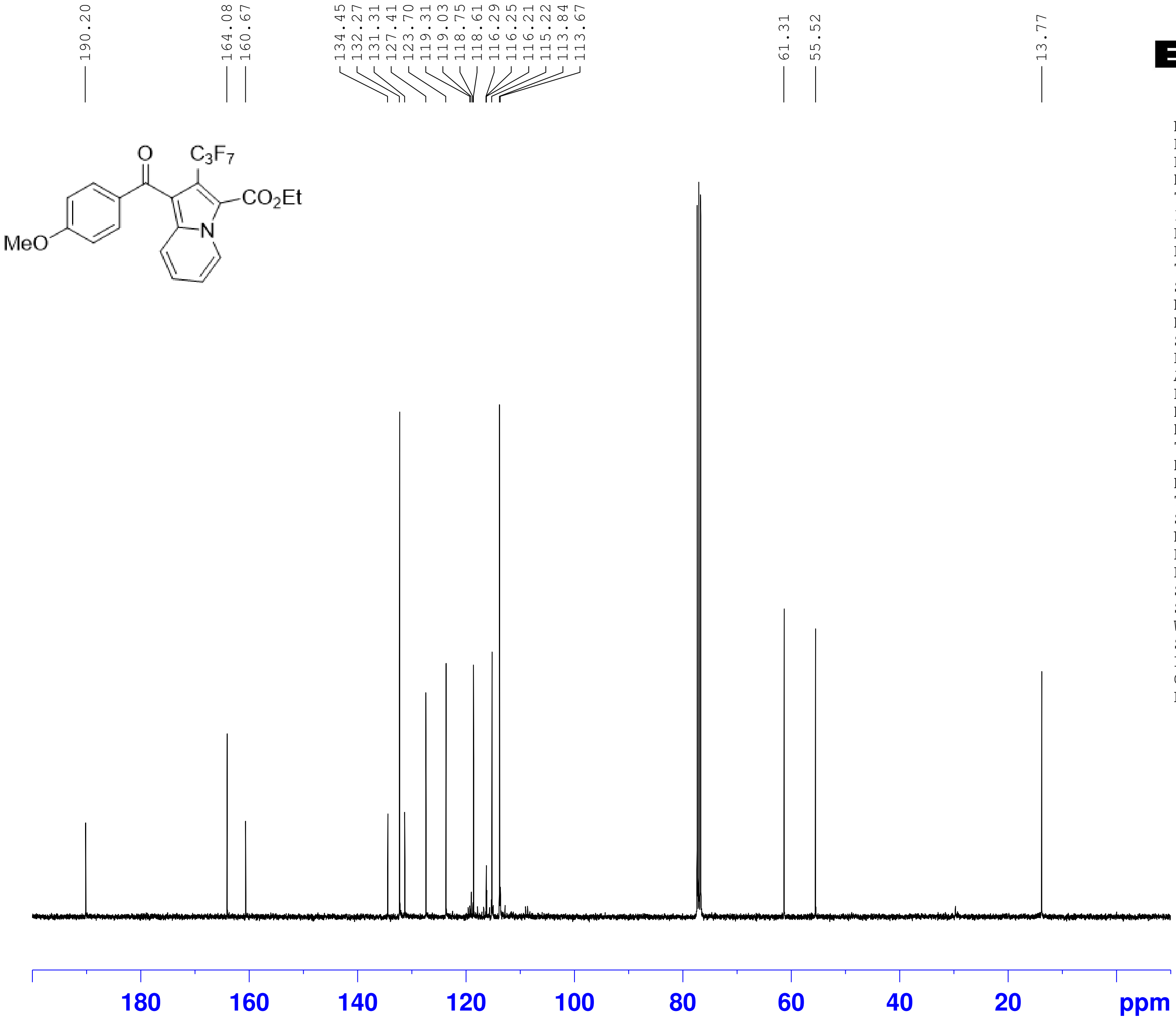

BRUKER

NAME	LV-MM-87-1
EXPNO	114
PROCNO	1
Date_	20240812
Time	11.46 h
INSTRUM	Avance
PROBHD	Z163739_0744 (
PULPROG	zgpg30
TD	65536
SOLVENT	CDCl3
NS	1024
DS	4
SWH	23809.523 Hz
FIDRES	0.726609 Hz
AQ	1.3763061 se
RG	101
DW	21.000 us
DE	6.50 us
TE	298.0 K
D1	2.00000000 se
D11	0.03000000 se
TD0	1
SFO1	100.6228298 MH
NUC1	13C
P0	2.67 us
P1	8.00 us
SI	32768
SF	100.6127685 MH
WDW	EM
SSB	0
LB	1.00 Hz
GB	0
PC	1.40

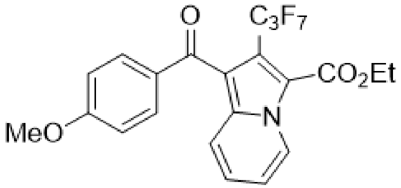

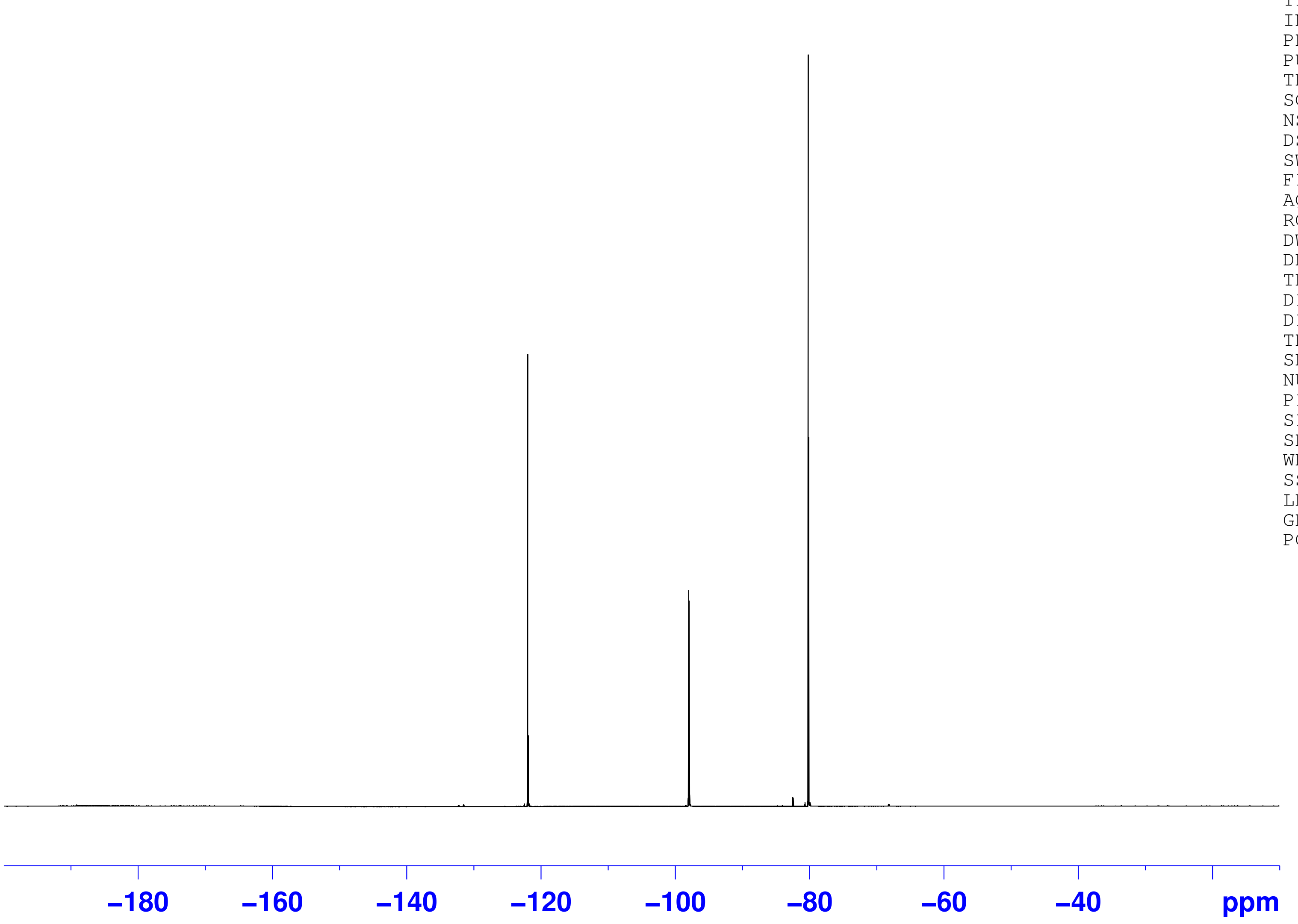

NAME	LV-MM-87-1	
EXPNO	115	
PROCNO	1	
Date_	20240812	
Time	11.48	h
INSTRUM	Avance	
PROBHD	Z163739_0744 (	
PULPROG	zgig	
TD	131072	
SOLVENT	CDCl3	
NS	16	
DS	4	
SWH	90909.094	Hz
FIDRES	1.387163	Hz
AQ	0.7209460	se
RG	101	
DW	5.500	us
DE	6.50	us
TE	298.1	K
D1	1.00000000	se
D11	0.03000000	se
TD0	1	
SFO1	376.4607164	MH
NUC1	19F	
P1	12.00	us
SI	65536	
SF	376.4983662	MH
WDW	EM	
SSB	0	
LB	0.30	Hz
GB	0	
PC	1.00	

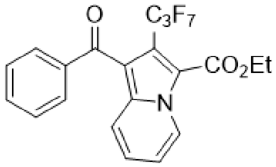

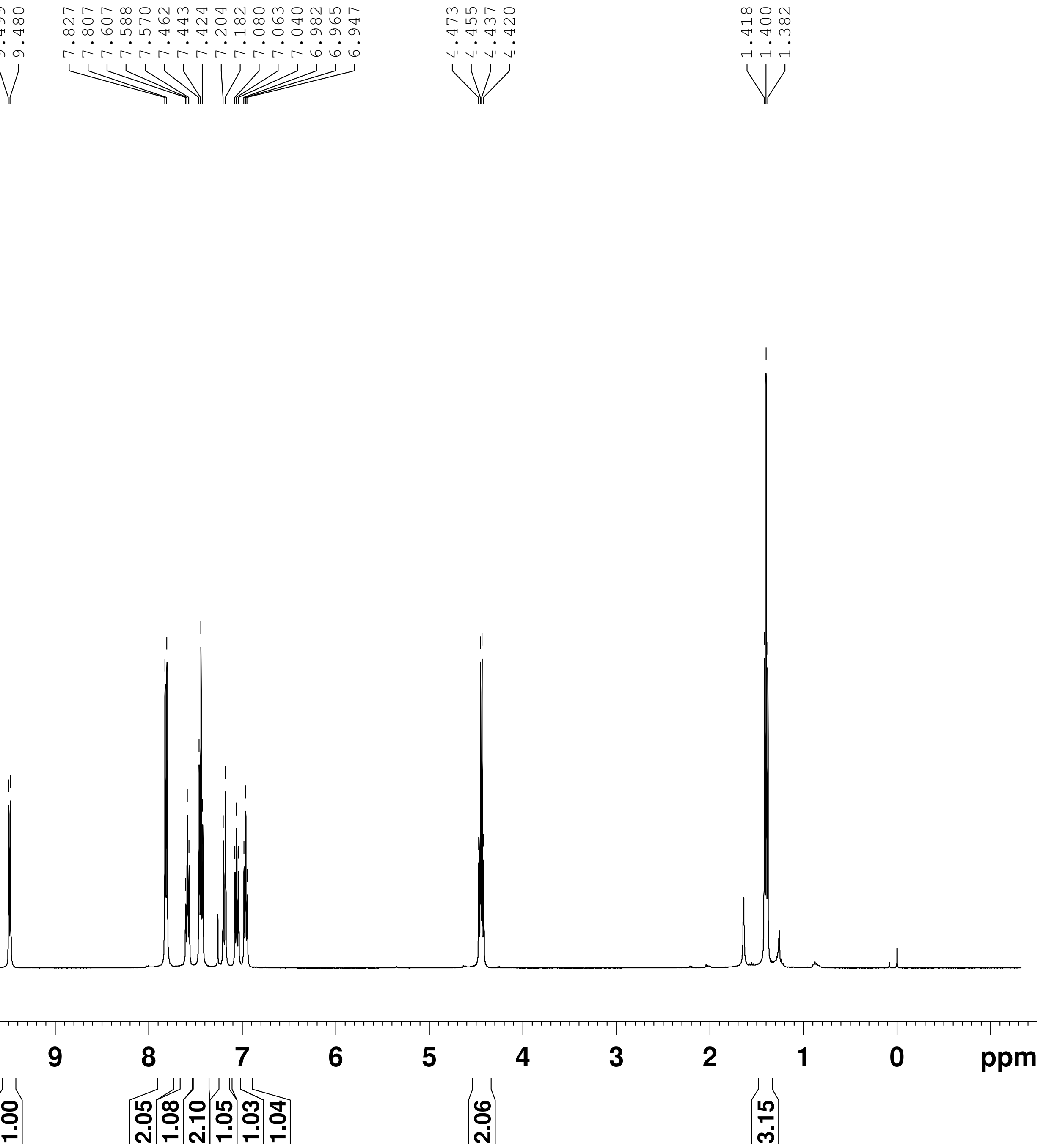

NAME	LV-MM-86
EXPNO	103
PROCNO	1
Date_	20240812
Time	9.38 h
INSTRUM	Avance
PROBHD	Z163739_0744 (
PULPROG	zg30
TD	65536
SOLVENT	CDCl3
NS	8
DS	0
SWH	6250.000 Hz
FIDRES	0.190735 Hz
AQ	5.2429299 sec
RG	90.5
DW	80.000 usec
DE	8.64 usec
TE	298.0 K
D1	1.00000000 sec
TD0	1
SFO1	400.1326008 MHz
NUC1	1H
P0	2.67 usec
P1	8.00 usec
SI	65536
SF	400.1300089 MHz
WDW	EM
SSB	0
LB	0.30 Hz
GB	0
PC	1.00

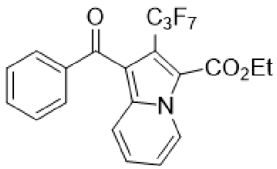

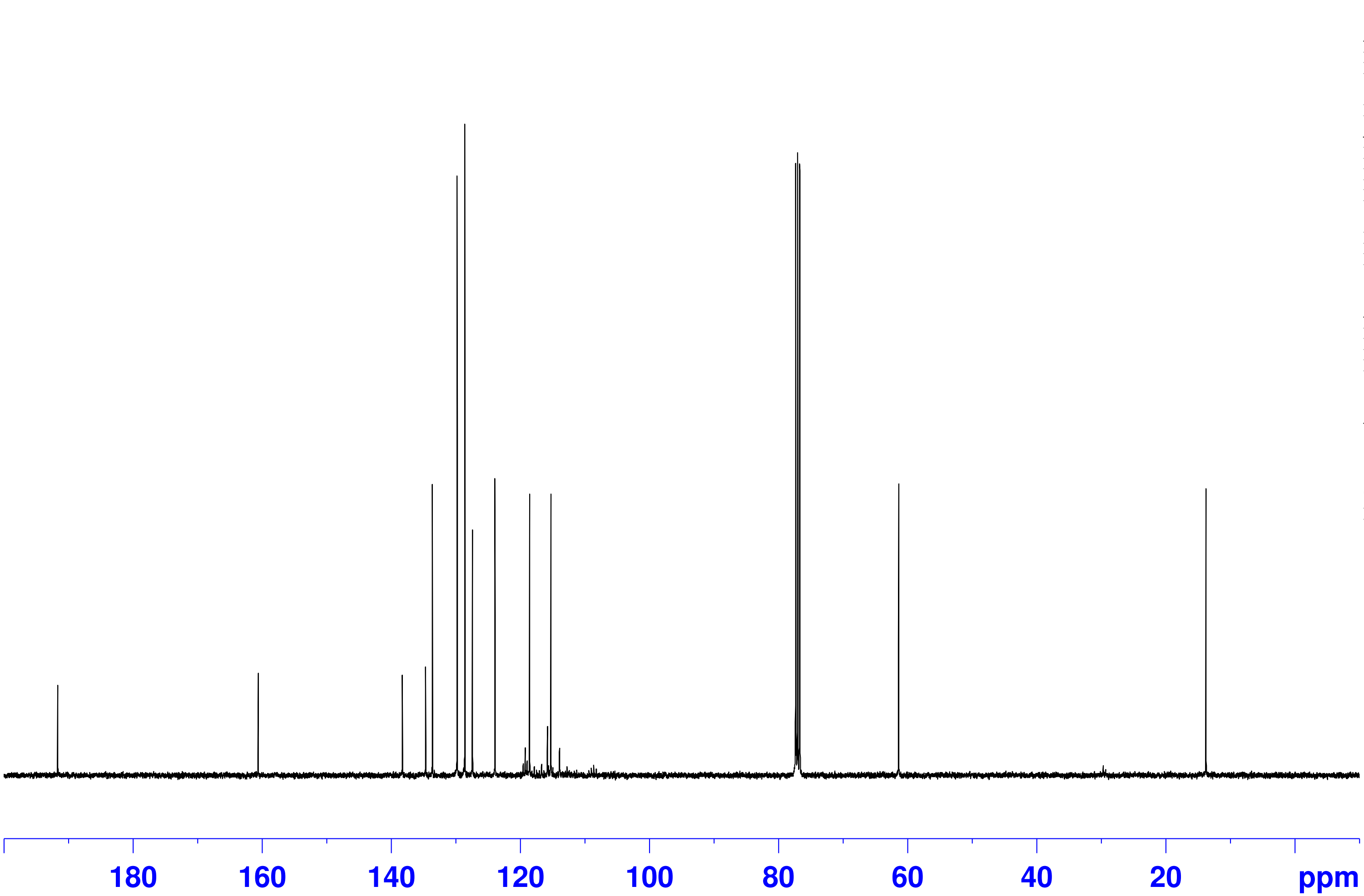

NAME	LV-MM-86
EXPNO	104
PROCNO	1
Date_	20240812
Time	10.38 h
INSTRUM	Avance
PROBHD	Z163739_0744 (
PULPROG	zgpg30
TD	65536
SOLVENT	CDCl3
NS	1024
DS	4
SWH	23809.523 Hz
FIDRES	0.726609 Hz
AQ	1.3763061 se
RG	101
DW	21.000 us
DE	6.50 us
TE	298.0 K
D1	2.00000000 se
D11	0.03000000 se
TD0	1
SFO1	100.6228298 MH
NUC1	13C
P0	2.67 us
P1	8.00 us
SI	32768
SF	100.6127685 MH
WDW	EM
SSB	0
LB	1.00 Hz
GB	0
PC	1.40

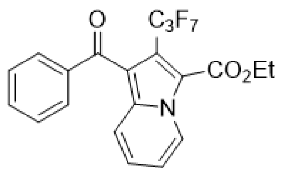

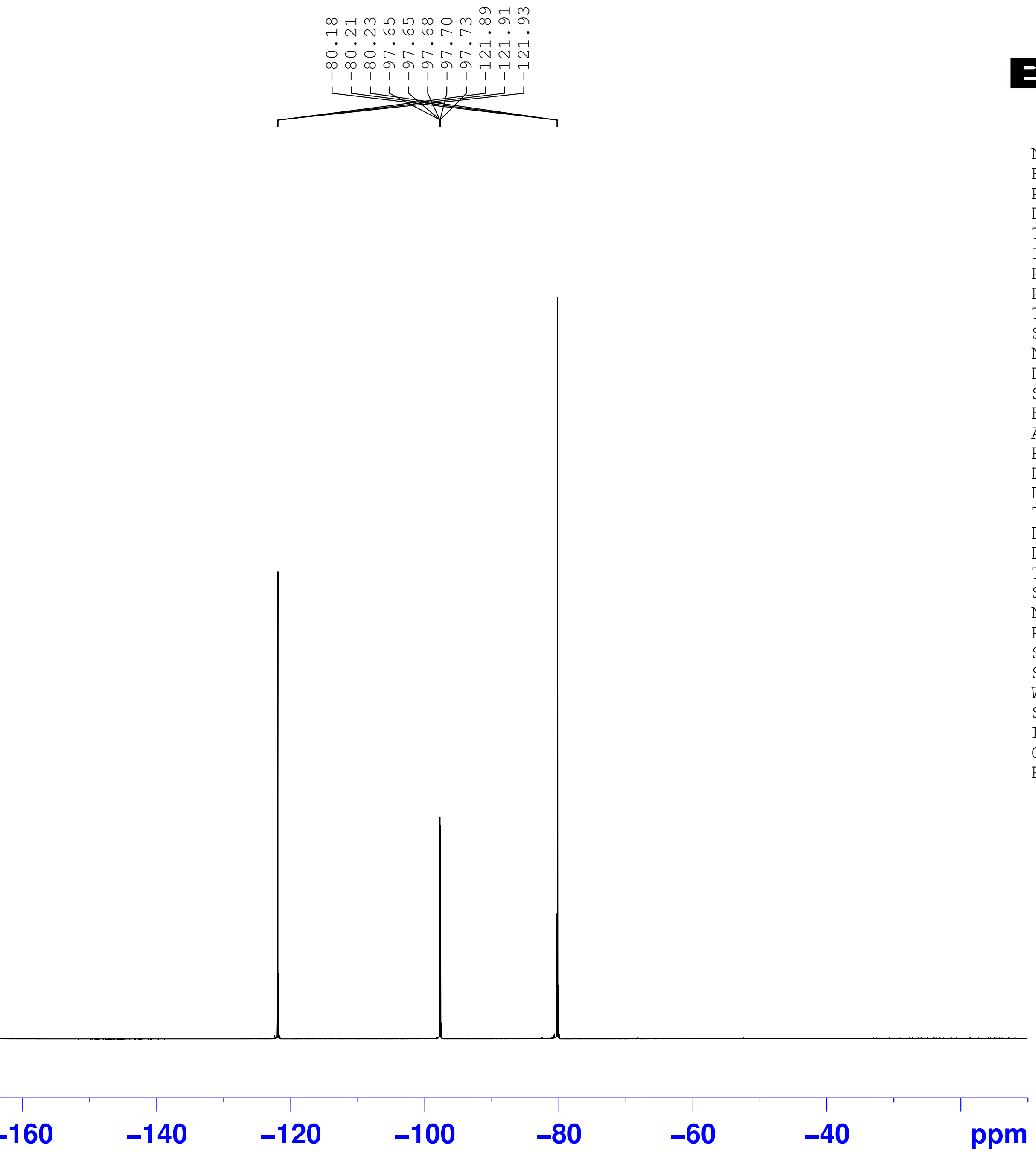

NAME	LV-MM-86
EXPNO	105
PROCNO	1
Date_	20240812
Time	10.41 h
INSTRUM	Avance
PROBHD	Z163739_0744 (
PULPROG	zgig
TD	131072
SOLVENT	CDCl3
NS	16
DS	4
SWH	90909.094 Hz
FIDRES	1.387163 Hz
AQ	0.7209460 se
RG	101
DW	5.500 us
DE	6.50 us
TE	298.1 K
D1	1.00000000 se
D11	0.03000000 se
TD0	1
SFO1	376.4607164 MH
NUC1	19F
P1	12.00 us
SI	65536
SF	376.4983662 MH
WDW	EM
SSB	0
LB	0.30 Hz
GB	0
PC	1.00

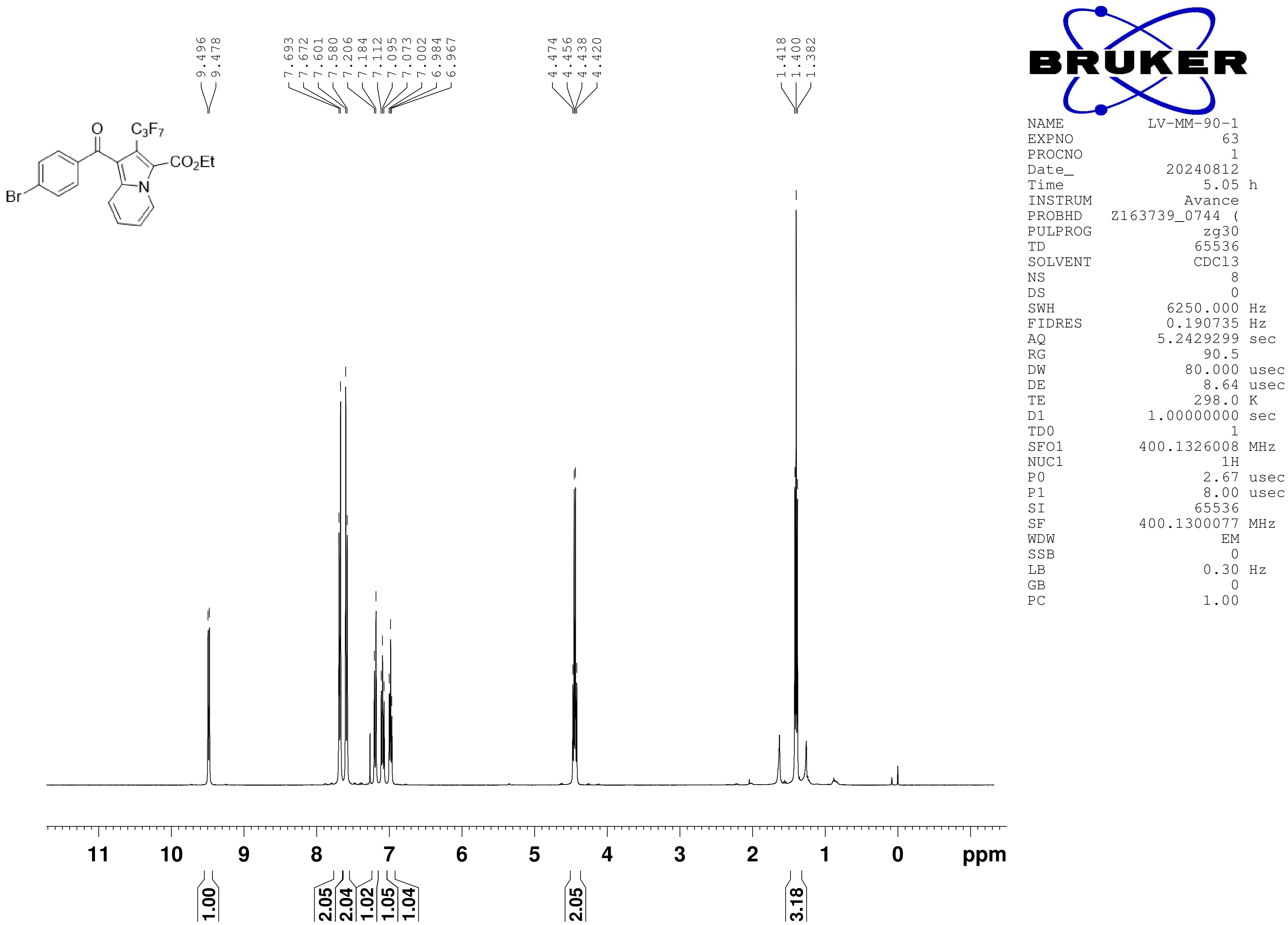

O
C3F7
CO2Et
Br
N
9.496
9.478
7.693
7.672
7.601
7.580
7.206
7.184
7.112
7.095
7.073
7.002
6.984
6.967
4.474
4.456
4.438
4.420
1.418
1.400
1.382
BRUKER
NAME LV-MM-90-1
EXPNO 63
PROCNO 1
Date_ 20240812
Time 5.05 h
INSTRUM Avance
PROBHD Z163739_0744 (
PULPROG zg30
TD 65536
SOLVENT CDCl3
NS 8
DS 0
SWH 6250.000 Hz
FIDRES 0.190735 Hz
AQ 5.2429299 sec
RG 90.5
DW 80.000 usec
DE 8.64 usec
TE 298.0 K
D1 1.00000000 sec
TD0 1
SFO1 400.1326008 MHz
NUC1 1H
P0 2.67 usec
P1 8.00 usec
SI 65536
SF 400.1300077 MHz
WDW EM
SSB 0
LB 0.30 Hz
GB 0
PC 1.00
11 10 9 8 7 6 5 4 3 2 1 0 ppm
1.00
2.05
2.04
1.02
1.05
1.04
2.05
3.18

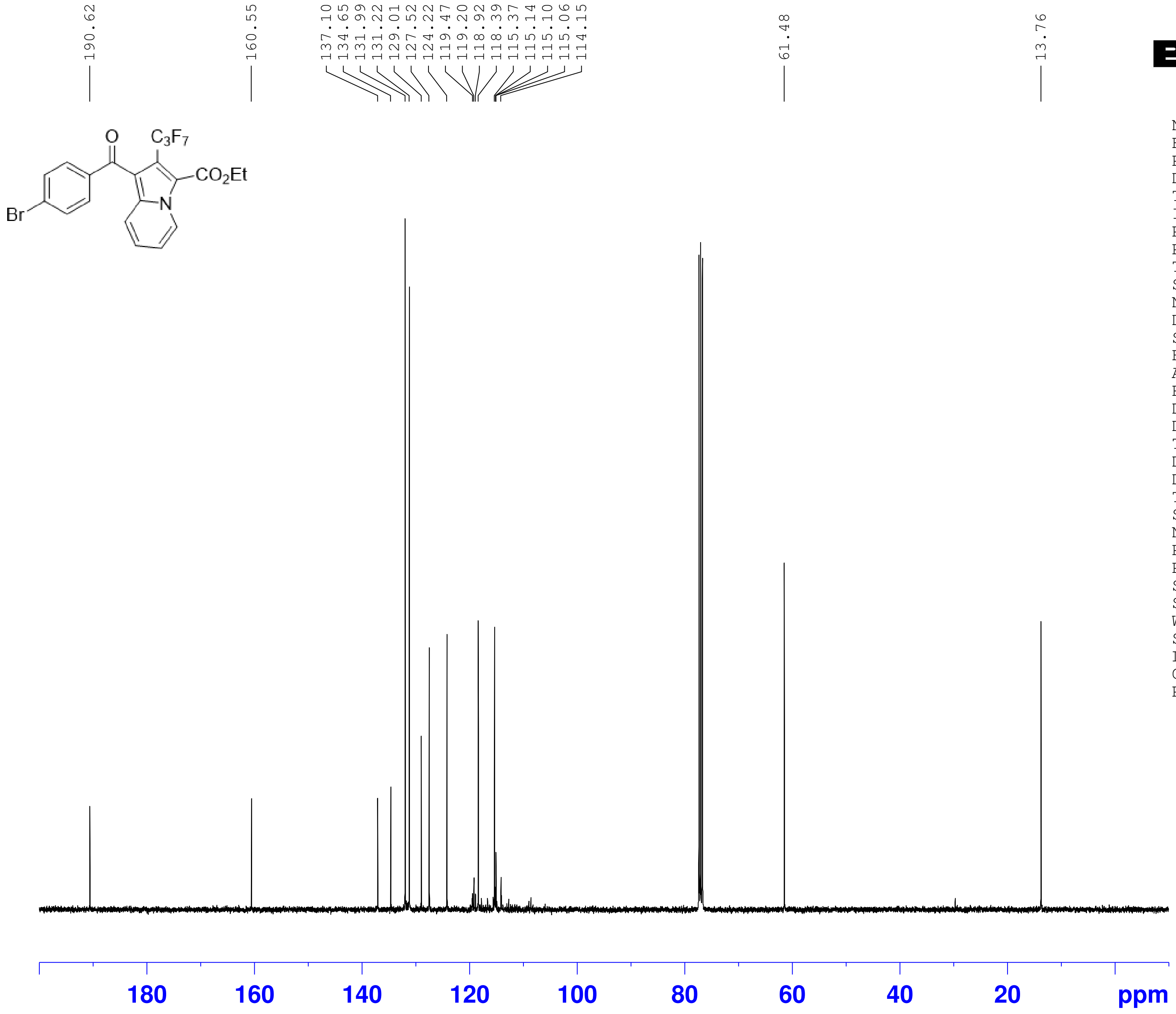

NAME	LV-MM-90-1
EXPNO	64
PROCNO	1
Date_	20240812
Time	6.05 h
INSTRUM	Avance
PROBHD	Z163739_0744 (
PULPROG	zgpg30
TD	65536
SOLVENT	CDCl3
NS	1024
DS	4
SWH	23809.523 Hz
FIDRES	0.726609 Hz
AQ	1.3763061 se
RG	101
DW	21.000 us
DE	6.50 us
TE	298.0 K
D1	2.00000000 se
D11	0.03000000 se
TD0	1
SFO1	100.6228298 MH
NUC1	13C
P0	2.67 us
P1	8.00 us
SI	32768
SF	100.6127685 MH
WDW	EM
SSB	0
LB	1.00 Hz
GB	0
PC	1.40

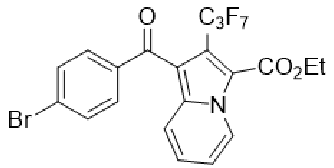

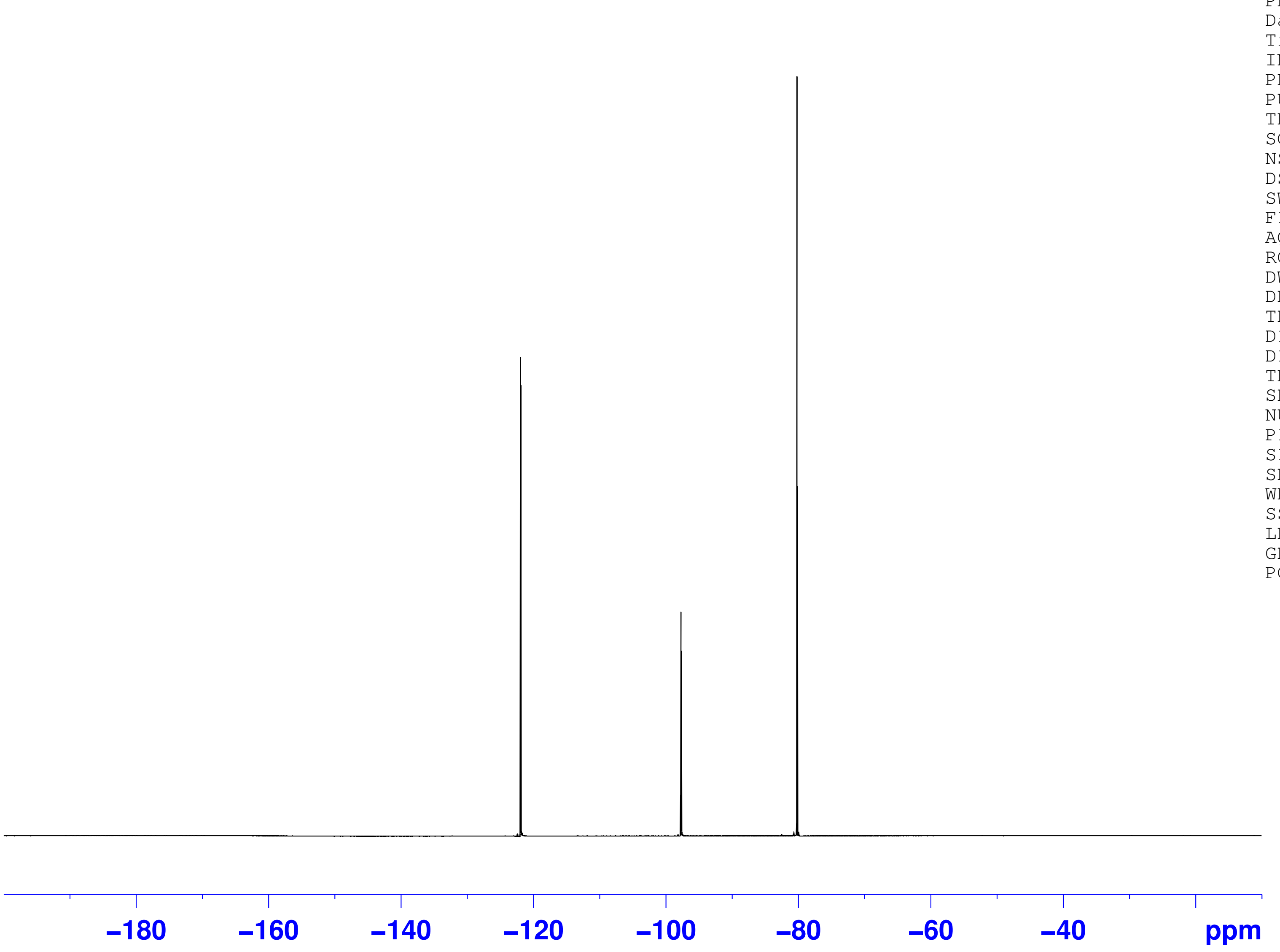

NAME	LV-MM-90-1
EXPNO	65
PROCNO	1
Date_	20240812
Time	6.07 h
INSTRUM	Avance
PROBHD	Z163739_0744 (
PULPROG	zgig
TD	131072
SOLVENT	CDCl3
NS	16
DS	4
SWH	90909.094 Hz
FIDRES	1.387163 Hz
AQ	0.7209460 se
RG	101
DW	5.500 us
DE	6.50 us
TE	298.1 K
D1	1.00000000 se
D11	0.03000000 se
TD0	1
SFO1	376.4607164 MH
NUC1	19F
P1	12.00 us
SI	65536
SF	376.4983662 MH
WDW	EM
SSB	0
LB	0.30 Hz
GB	0
PC	1.00

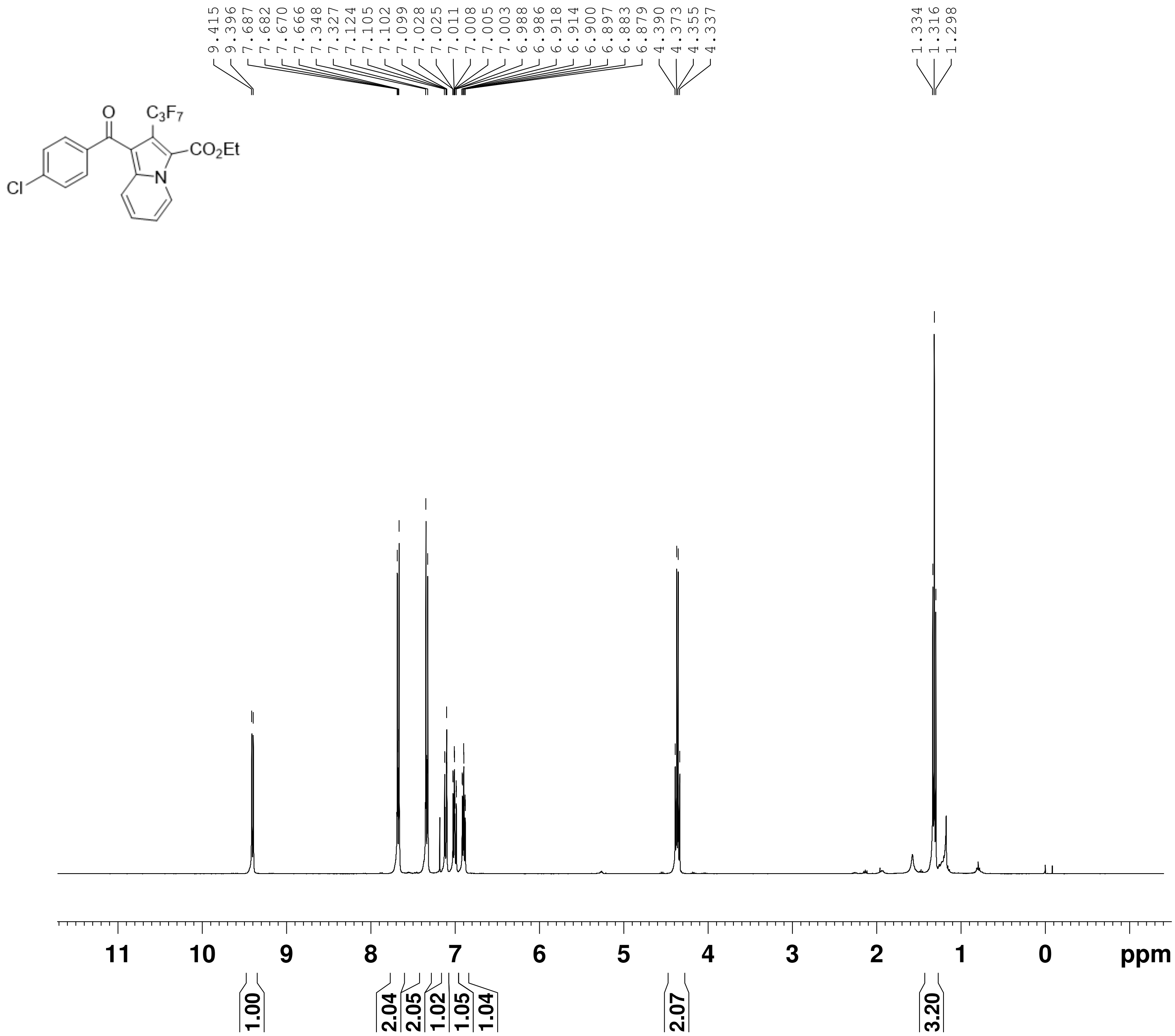

BRUKER

NAME	LV-MM-94-20240812
EXPNO	163
PROCNO	1
Date_	20240812
Time	14.52 h
INSTRUM	Avance
PROBHD	Z163739_0744 (
PULPROG	zg30
TD	65536
SOLVENT	CDCl3
NS	8
DS	0
SWH	6250.000 Hz
FIDRES	0.190735 Hz
AQ	5.2429299 sec
RG	90.5
DW	80.000 usec
DE	8.64 usec
TE	298.0 K
D1	1.00000000 sec
TD0	1
SFO1	400.1326008 MHz
NUC1	1H
P0	2.67 usec
P1	8.00 usec
SI	65536
SF	400.1300405 MHz
WDW	EM
SSB	0
LB	0.30 Hz
GB	0
PC	1.00

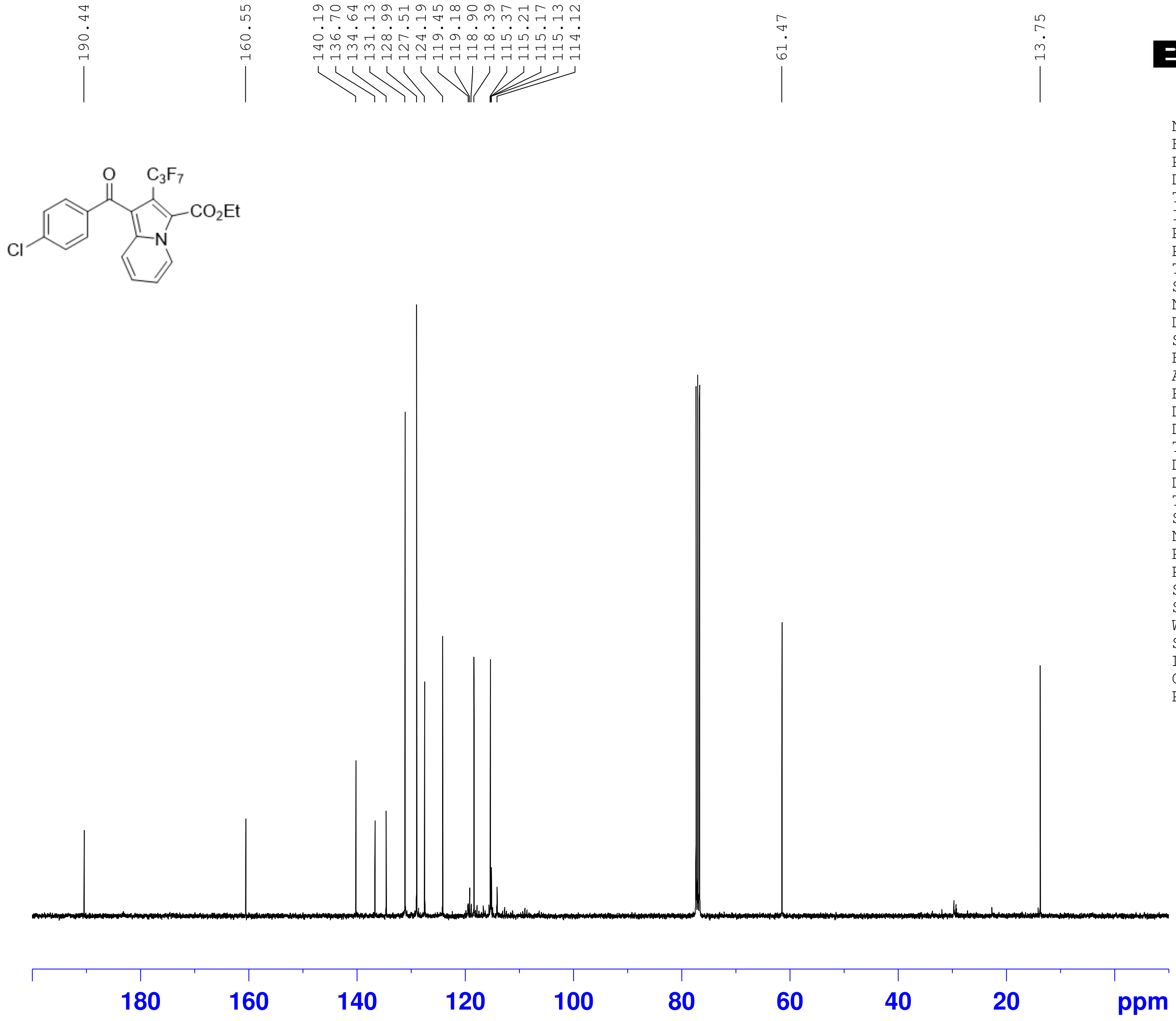

NAME	LV-MM-94-20240812
EXPNO	164
PROCNO	1
Date_	20240812
Time	15.40 h
INSTRUM	Avance
PROBHD	Z163739_0744 (
PULPROG	zgpg30
TD	65536
SOLVENT	CDCl3
NS	800
DS	4
SWH	23809.523 Hz
FIDRES	0.726609 Hz
AQ	1.3763061 se
RG	101
DW	21.000 us
DE	6.50 us
TE	298.0 K
D1	2.00000000 se
D11	0.03000000 se
TD0	1
SFO1	100.6228298 MH
NUC1	13C
P0	2.67 us
P1	8.00 us
SI	32768
SF	100.6127685 MH
WDW	EM
SSB	0
LB	1.00 Hz
GB	0
PC	1.40

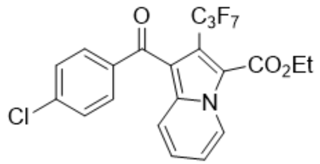

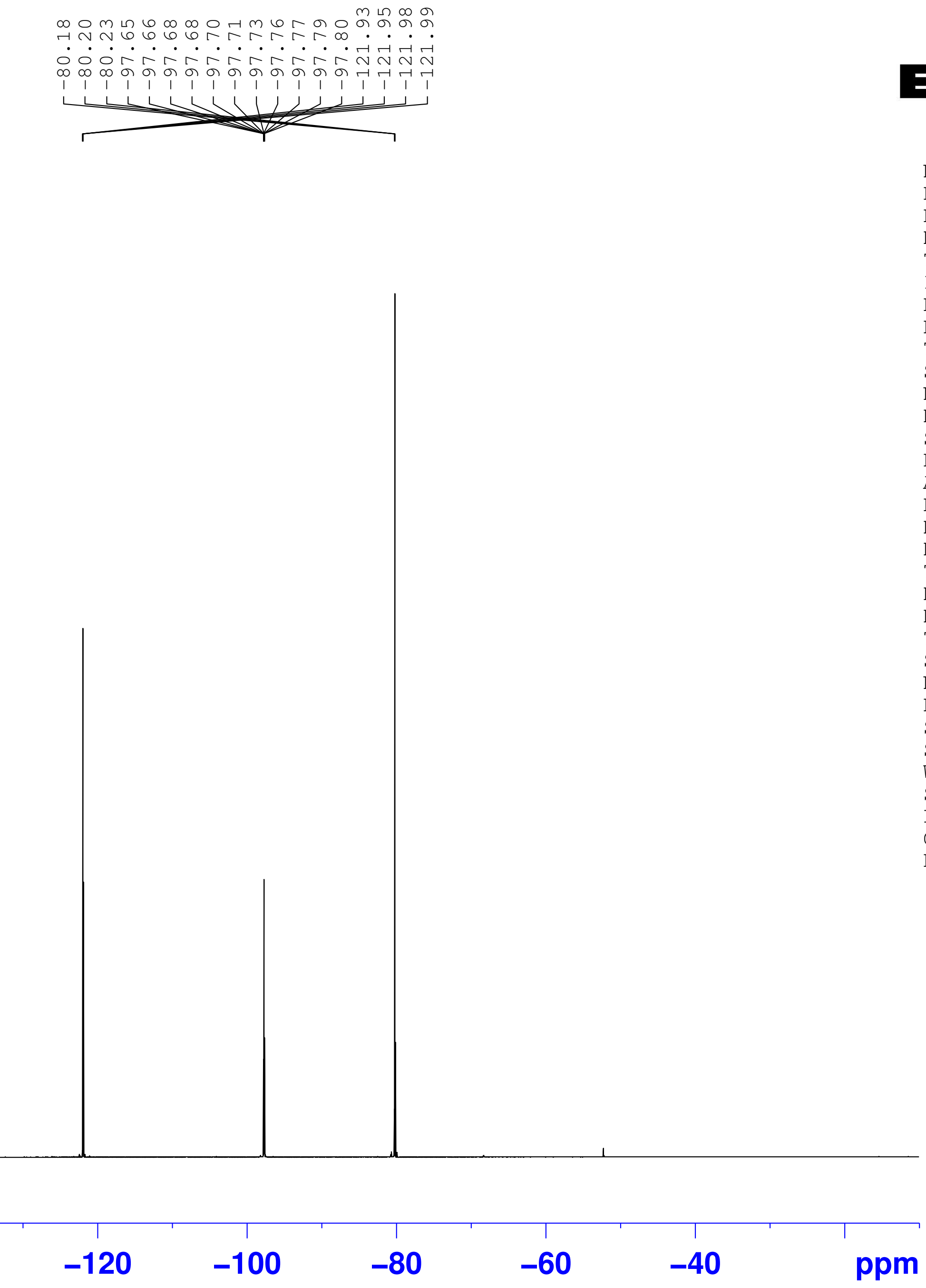

NAME	LV-MM-94-20240812
EXPNO	155
PROCNO	1
Date_	20240812
Time	15.42 h
INSTRUM	Avance
PROBHD	Z163739_0744 (
PULPROG	zgig
TD	131072
SOLVENT	CDCl3
NS	16
DS	4
SWH	90909.094 Hz
FIDRES	1.387163 Hz
AQ	0.7209460 se
RG	101
DW	5.500 us
DE	6.50 us
TE	298.1 K
D1	1.00000000 se
D11	0.03000000 se
TD0	1
SFO1	376.4607164 MH
NUC1	19F
P1	12.00 us
SI	65536
SF	376.4983662 MH
WDW	EM
SSB	0
LB	0.30 Hz
GB	0
PC	1.00

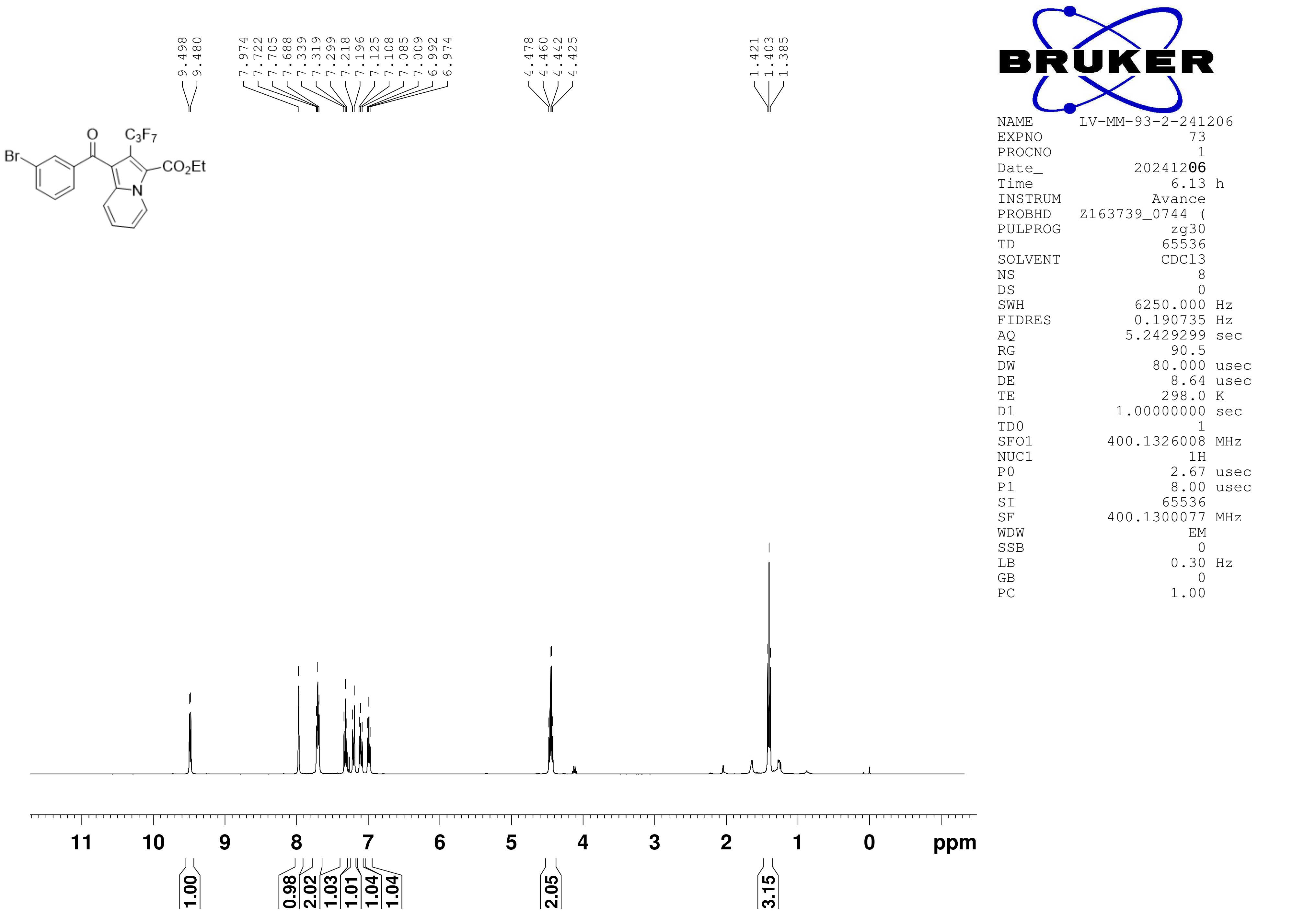
9.498
9.480
7.974
7.722
7.705
7.688
7.339
7.319
7.299
7.218
7.196
7.125
7.108
7.085
7.009
6.992
6.974
4.478
4.460
4.442
4.425
1.421
1.403
1.385
Br
O
C_3F_7
CO_2Et
N
BRUKER
NAME LV-MM-93-2-241206
EXPNO 73
PROCNO 1
Date_ 20241206
Time 6.13 h
INSTRUM Avance
PROBHD Z163739_0744 (
PULPROG zg30
TD 65536
SOLVENT CDCl3
NS 8
DS 0
SWH 6250.000 Hz
FIDRES 0.190735 Hz
AQ 5.2429299 sec
RG 90.5
DW 80.000 usec
DE 8.64 usec
TE 298.0 K
D1 1.00000000 sec
TD0 1
SFO1 400.1326008 MHz
NUC1 1H
P0 2.67 usec
P1 8.00 usec
SI 65536
SF 400.1300077 MHz
WDW EM
SSB 0
LB 0.30 Hz
GB 0
PC 1.00
11
10
9
8
7
6
5
4
3
2
1
0
ppm
1.00
0.98
2.02
1.03
1.01
1.04
1.04
2.05
3.15

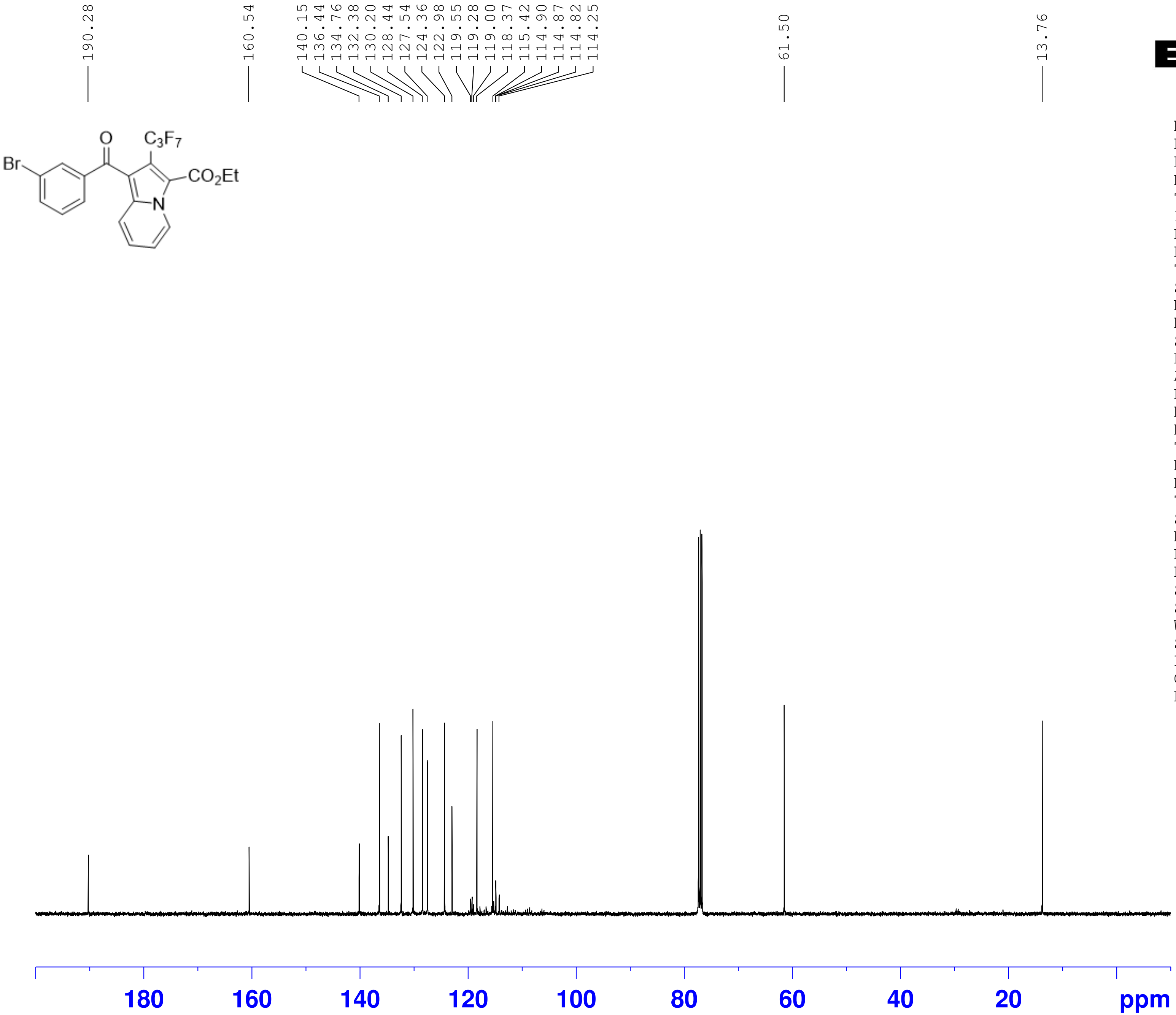

NAME	LV-MM-93-2-241206
EXPNO	74
PROCNO	1
Date_	20241206
Time	7.13 h
INSTRUM	Avance
PROBHD	Z163739_0744 (
PULPROG	zgpg30
TD	65536
SOLVENT	CDCl3
NS	1024
DS	4
SWH	23809.523 Hz
FIDRES	0.726609 Hz
AQ	1.3763061 sec
RG	101
DW	21.000 usec
DE	6.50 usec
TE	298.0 K
D1	2.00000000 sec
D11	0.03000000 sec
TD0	1
SFO1	100.6228298 MHz
NUC1	13C
P0	2.67 usec
P1	8.00 usec
SI	32768
SF	100.6127685 MHz
WDW	EM
SSB	0
LB	1.00 Hz
GB	0
PC	1.40

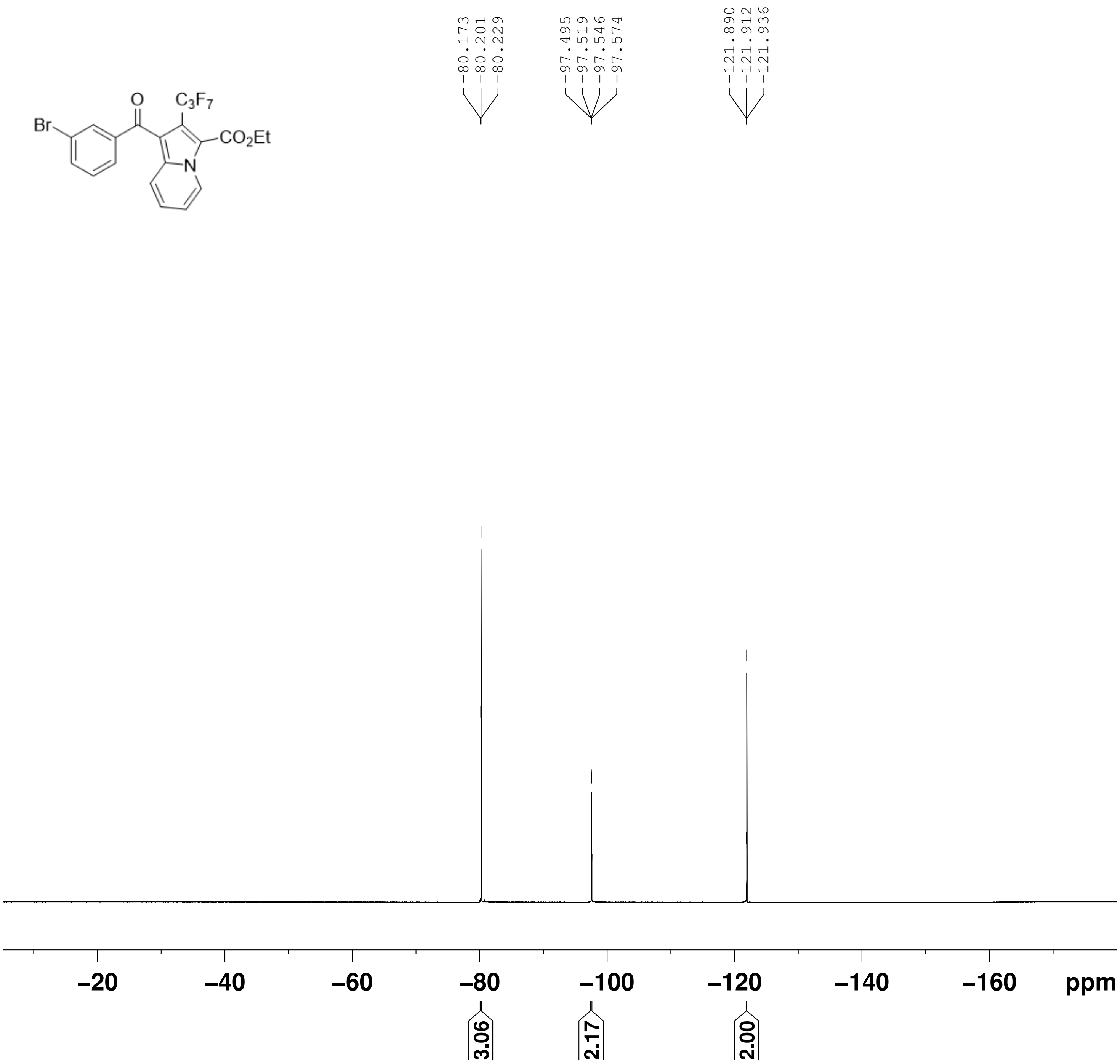

NAME	LV-MM-93-2-241206
EXPNO	75
PROCNO	1
Date_	20241206
Time	7.16 h
INSTRUM	Avance
PROBHD	Z163739_0744 (
PULPROG	zgig
TD	131072
SOLVENT	CDCl3
NS	16
DS	4
SWH	90909.094 Hz
FIDRES	1.387163 Hz
AQ	0.7209460 sec
RG	101
DW	5.500 usec
DE	6.50 usec
TE	298.1 K
D1	1.00000000 sec
D11	0.03000000 sec
TD0	1
SFO1	376.4607164 MHz
NUC1	19F
P1	12.00 usec
SI	65536
SF	376.4983662 MHz
WDW	EM
SSB	0
LB	0.30 Hz
GB	0
PC	1.00

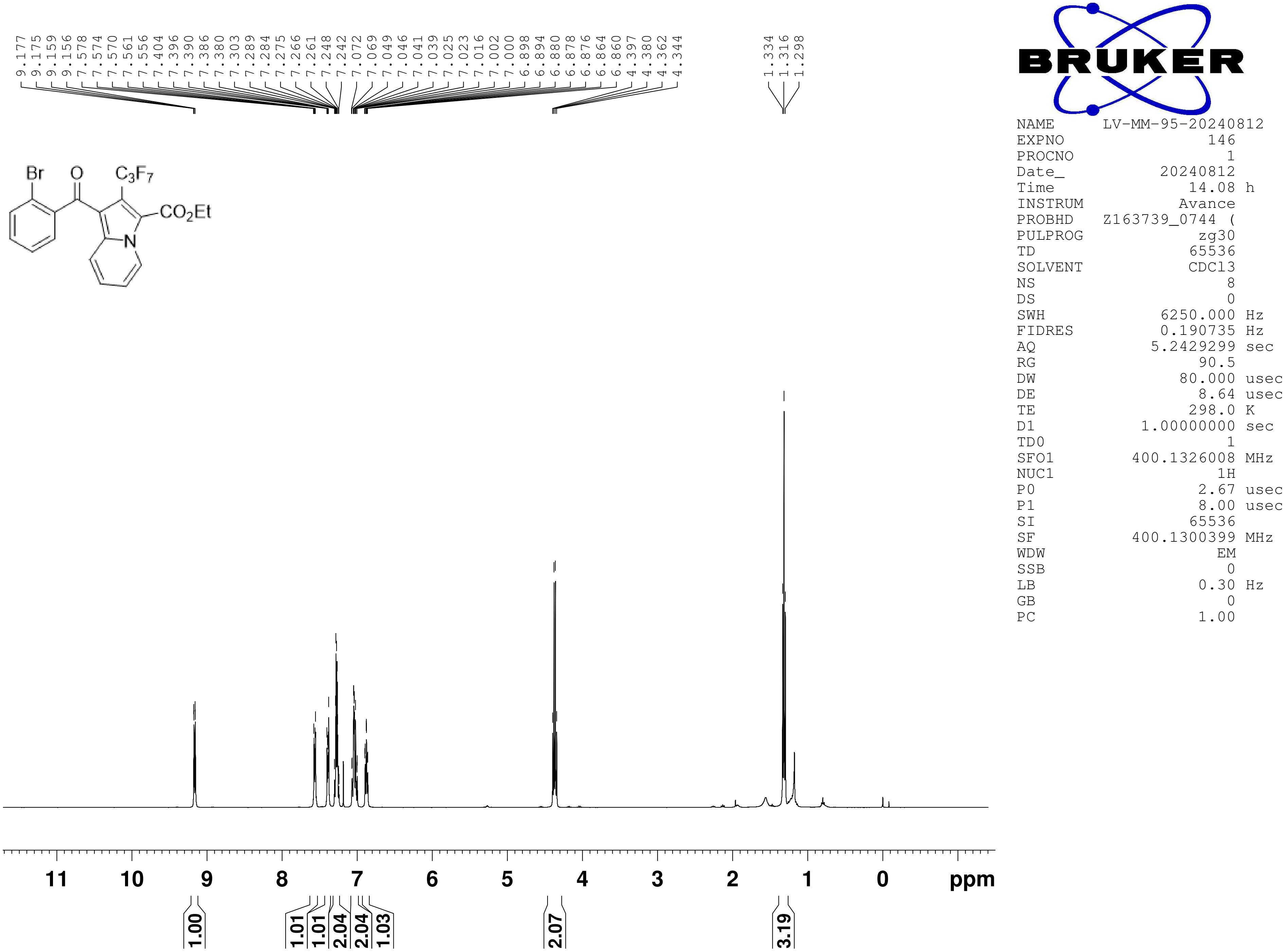
9.177
9.175
9.159
9.156
7.578
7.574
7.570
7.561
7.556
7.404
7.396
7.390
7.386
7.380
7.303
7.289
7.284
7.275
7.266
7.261
7.248
7.242
7.072
7.069
7.049
7.046
7.041
7.039
7.025
7.023
7.016
7.002
7.000
6.898
6.894
6.880
6.878
6.876
6.864
6.860
4.397
4.380
4.362
4.344
1.334
1.316
1.298
Br
O
C_3F_7
CO_2Et
N
BRUKER
NAME LV-MM-95-20240812
EXPNO 146
PROCNO 1
Date_ 20240812
Time 14.08 h
INSTRUM Avance
PROBHD Z163739_0744 (
PULPROG zg30
TD 65536
SOLVENT CDCl3
NS 8
DS 0
SWH 6250.000 Hz
FIDRES 0.190735 Hz
AQ 5.2429299 sec
RG 90.5
DW 80.000 usec
DE 8.64 usec
TE 298.0 K
D1 1.00000000 sec
TD0 1
SFO1 400.1326008 MHz
NUC1 1H
P0 2.67 usec
P1 8.00 usec
SI 65536
SF 400.1300399 MHz
WDW EM
SSB 0
LB 0.30 Hz
GB 0
PC 1.00
11
10
9
8
7
6
5
4
3
2
1
0
ppm
1.00
1.01
1.01
2.04
2.04
1.03
2.07
3.19

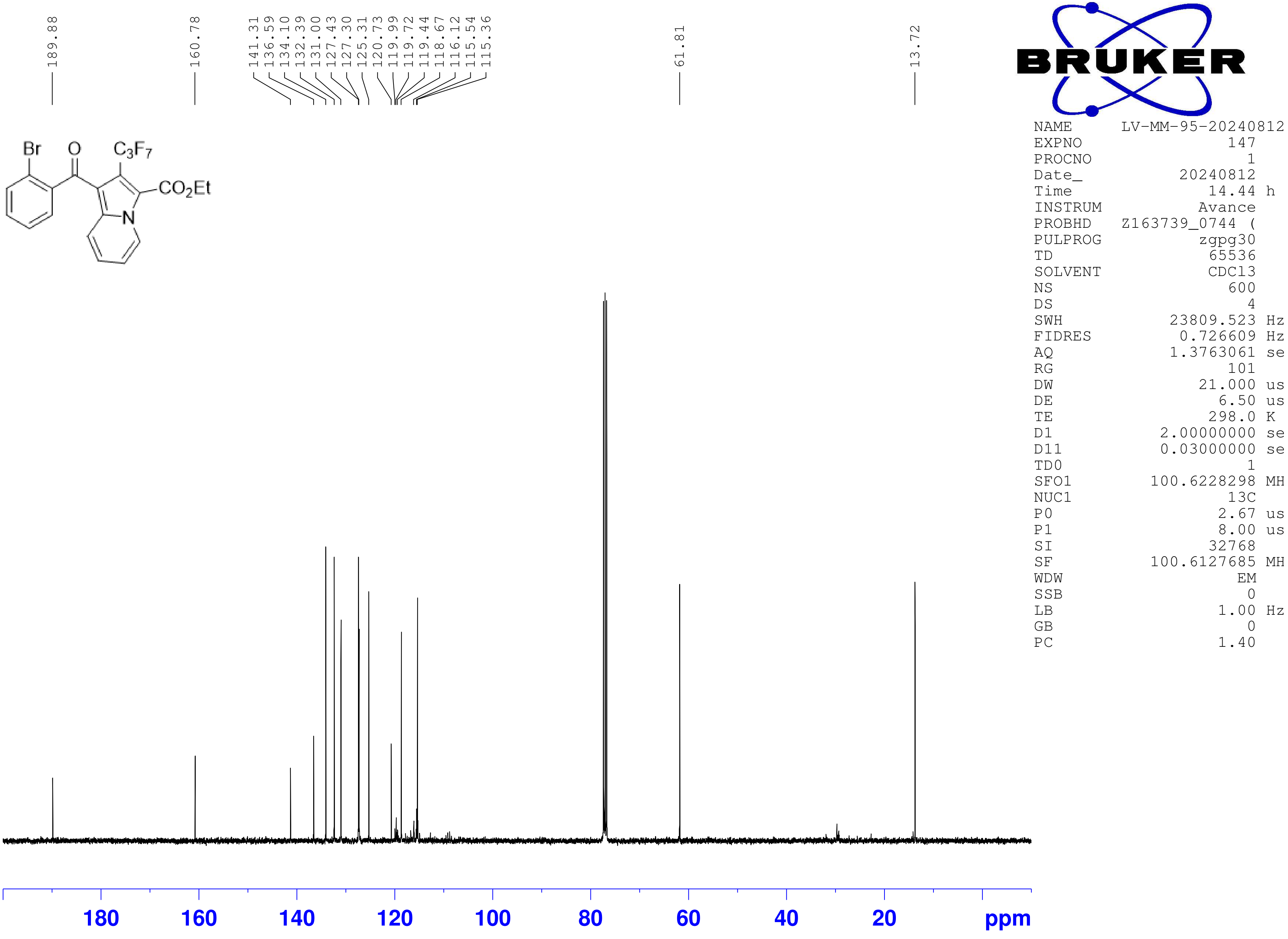

189.88
160.78
141.31
136.59
134.10
132.39
131.00
127.43
127.30
125.31
120.73
119.99
119.72
119.44
118.67
116.12
115.54
115.36
61.81
13.72
Br
O
C3F7
CO2Et
N
NAME LV-MM-95-20240812
EXPNO 147
PROCNO 1
Date_ 20240812
Time 14.44 h
INSTRUM Avance
PROBHD Z163739_0744 (
PULPROG zgpg30
TD 65536
SOLVENT CDCl3
NS 600
DS 4
SWH 23809.523 Hz
FIDRES 0.726609 Hz
AQ 1.3763061 se
RG 101
DW 21.000 us
DE 6.50 us
TE 298.0 K
D1 2.00000000 se
D11 0.03000000 se
TD0 1
SFO1 100.6228298 MH
NUC1 13C
P0 2.67 us
P1 8.00 us
SI 32768
SF 100.6127685 MH
WDW EM
SSB 0
LB 1.00 Hz
GB 0
PC 1.40
180
160
140
120
100
80
60
40
20
ppm

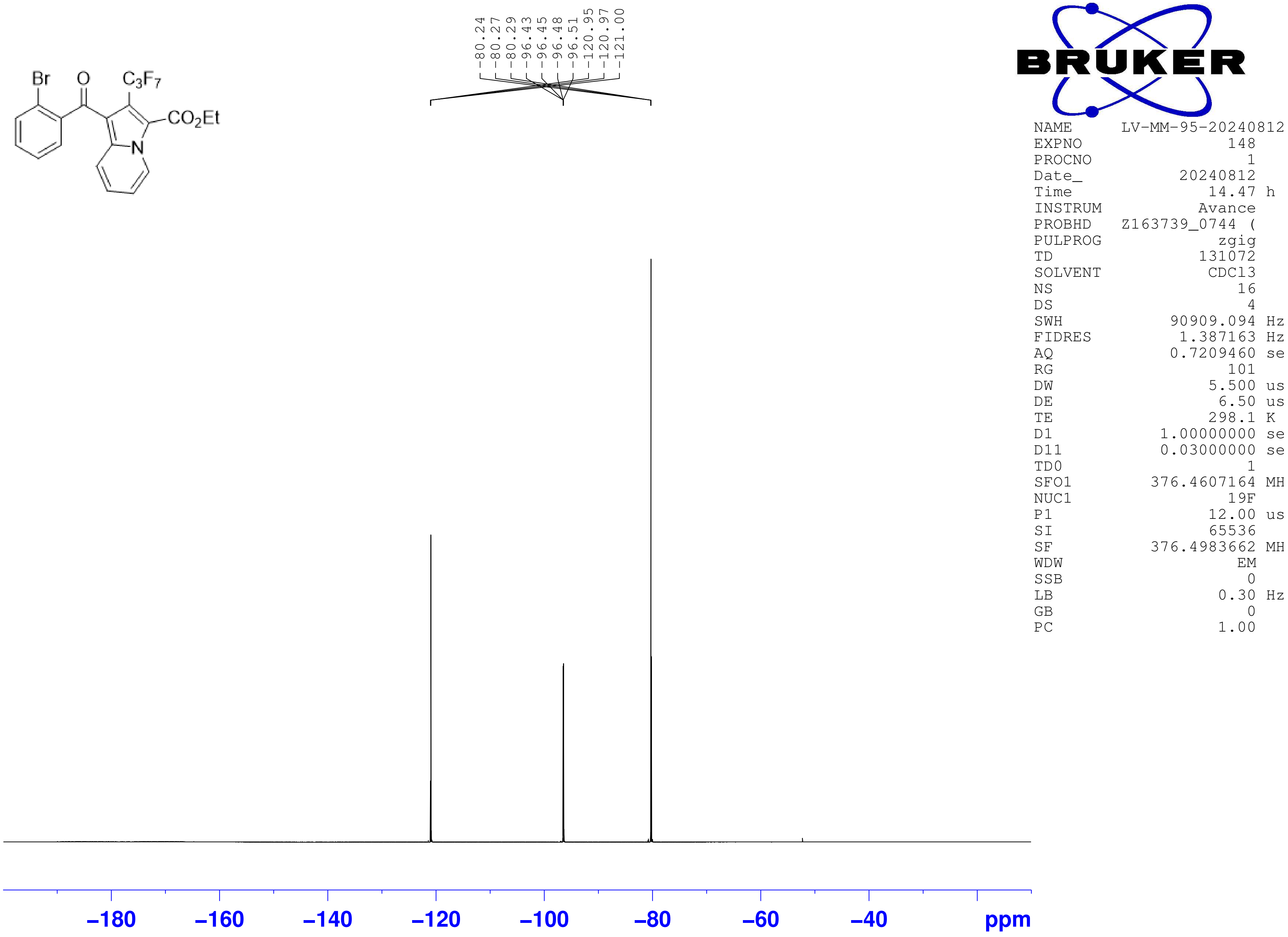
Br O C3F7
CO2Et
N
-80.24
-80.27
-80.29
-96.43
-96.45
-96.48
-96.51
-120.95
-120.97
-121.00
BRUKER
NAME LV-MM-95-20240812
EXPNO 148
PROCNO 1
Date_ 20240812
Time 14.47 h
INSTRUM Avance
PROBHD Z163739_0744 (
PULPROG zgig
TD 131072
SOLVENT CDCl3
NS 16
DS 4
SWH 90909.094 Hz
FIDRES 1.387163 Hz
AQ 0.7209460 se
RG 101
DW 5.500 us
DE 6.50 us
TE 298.1 K
D1 1.00000000 se
D11 0.03000000 se
TD0 1
SFO1 376.4607164 MH
NUC1 19F
P1 12.00 us
SI 65536
SF 376.4983662 MH
WDW EM
SSB 0
LB 0.30 Hz
GB 0
PC 1.00
-180
-160
-140
-120
-100
-80
-60
-40
ppm

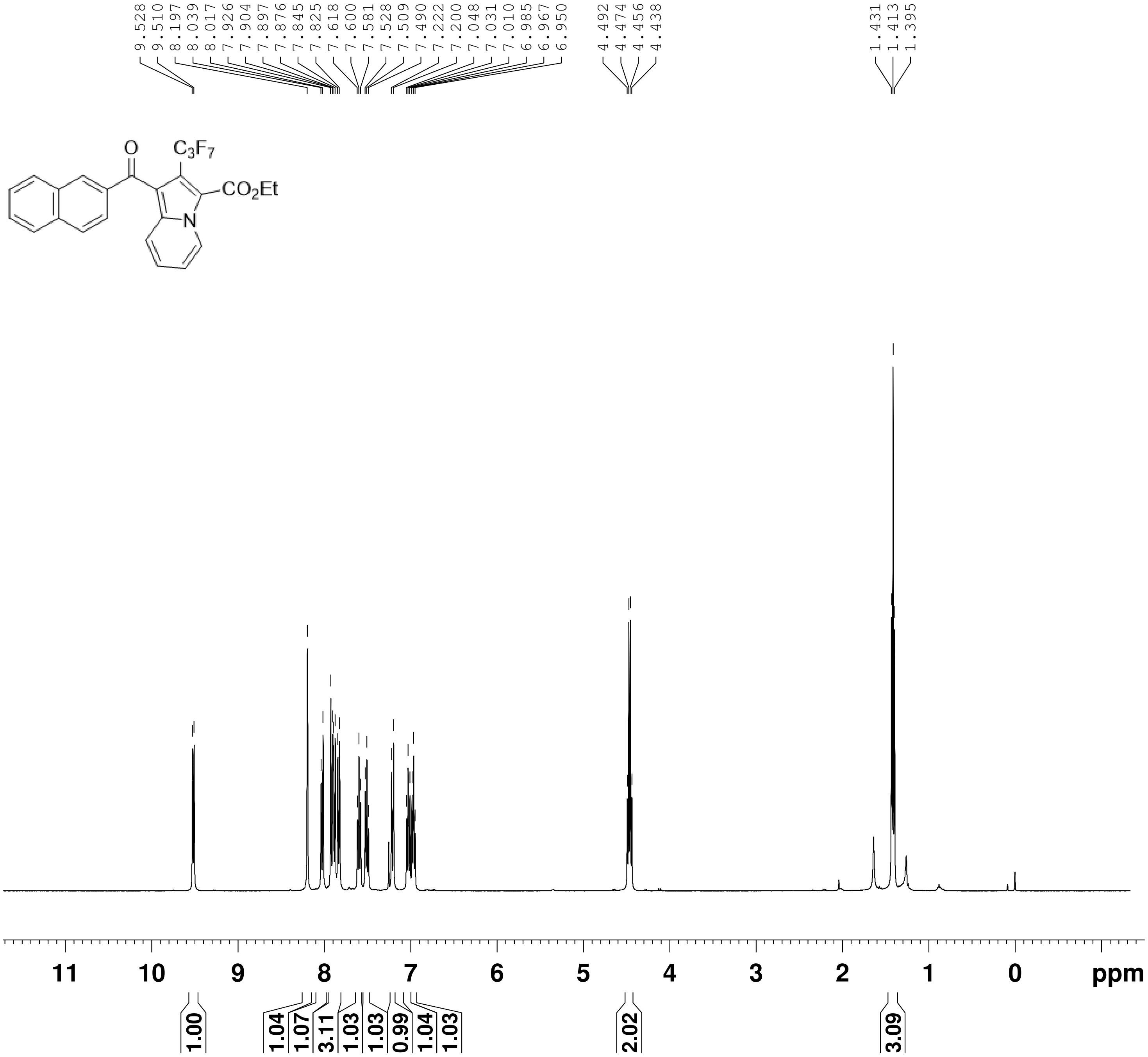

BRUKER

NAME	LV-MM-91-1
EXPNO	53
PROCNO	1
Date_	20240812
Time	3.57 h
INSTRUM	Avance
PROBHD	Z163739_0744 (
PULPROG	zg30
TD	65536
SOLVENT	CDCl3
NS	8
DS	0
SWH	6250.000 Hz
FIDRES	0.190735 Hz
AQ	5.2429299 sec
RG	101
DW	80.000 usec
DE	8.64 usec
TE	298.0 K
D1	1.00000000 sec
TD0	1
SFO1	400.1326008 MHz
NUC1	1H
P0	2.67 usec
P1	8.00 usec
SI	65536
SF	400.1300121 MHz
WDW	EM
SSB	0
LB	0.30 Hz
GB	0
PC	1.00

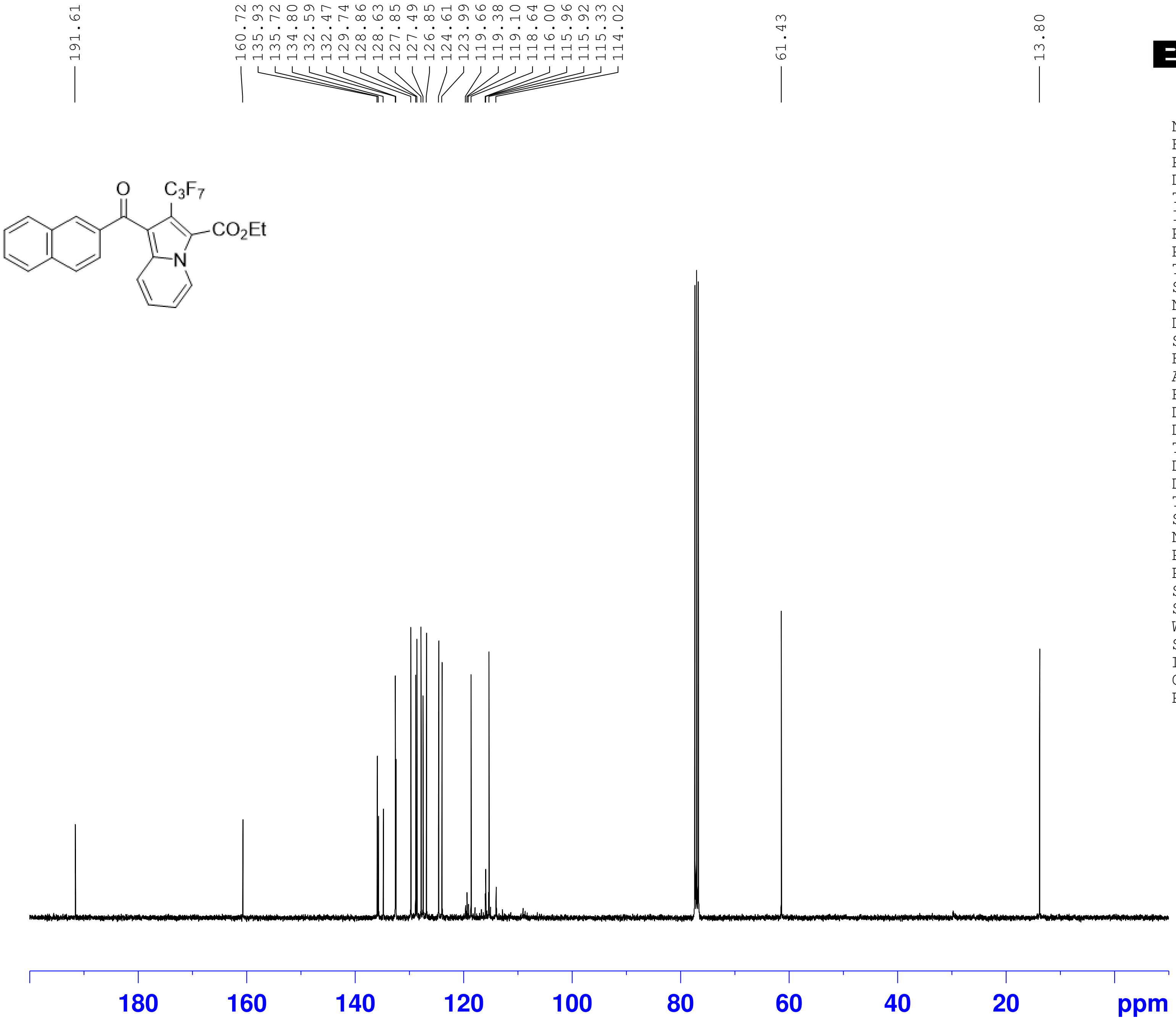

NAME	LV-MM-91-1
EXPNO	54
PROCNO	1
Date_	20240812
Time	4.58 h
INSTRUM	Avance
PROBHD	Z163739_0744 (
PULPROG	zgpg30
TD	65536
SOLVENT	CDCl3
NS	1024
DS	4
SWH	23809.523 Hz
FIDRES	0.726609 Hz
AQ	1.3763061 se
RG	101
DW	21.000 us
DE	6.50 us
TE	298.0 K
D1	2.00000000 se
D11	0.03000000 se
TD0	1
SFO1	100.6228298 MH
NUC1	13C
P0	2.67 us
P1	8.00 us
SI	32768
SF	100.6127685 MH
WDW	EM
SSB	0
LB	1.00 Hz
GB	0
PC	1.40

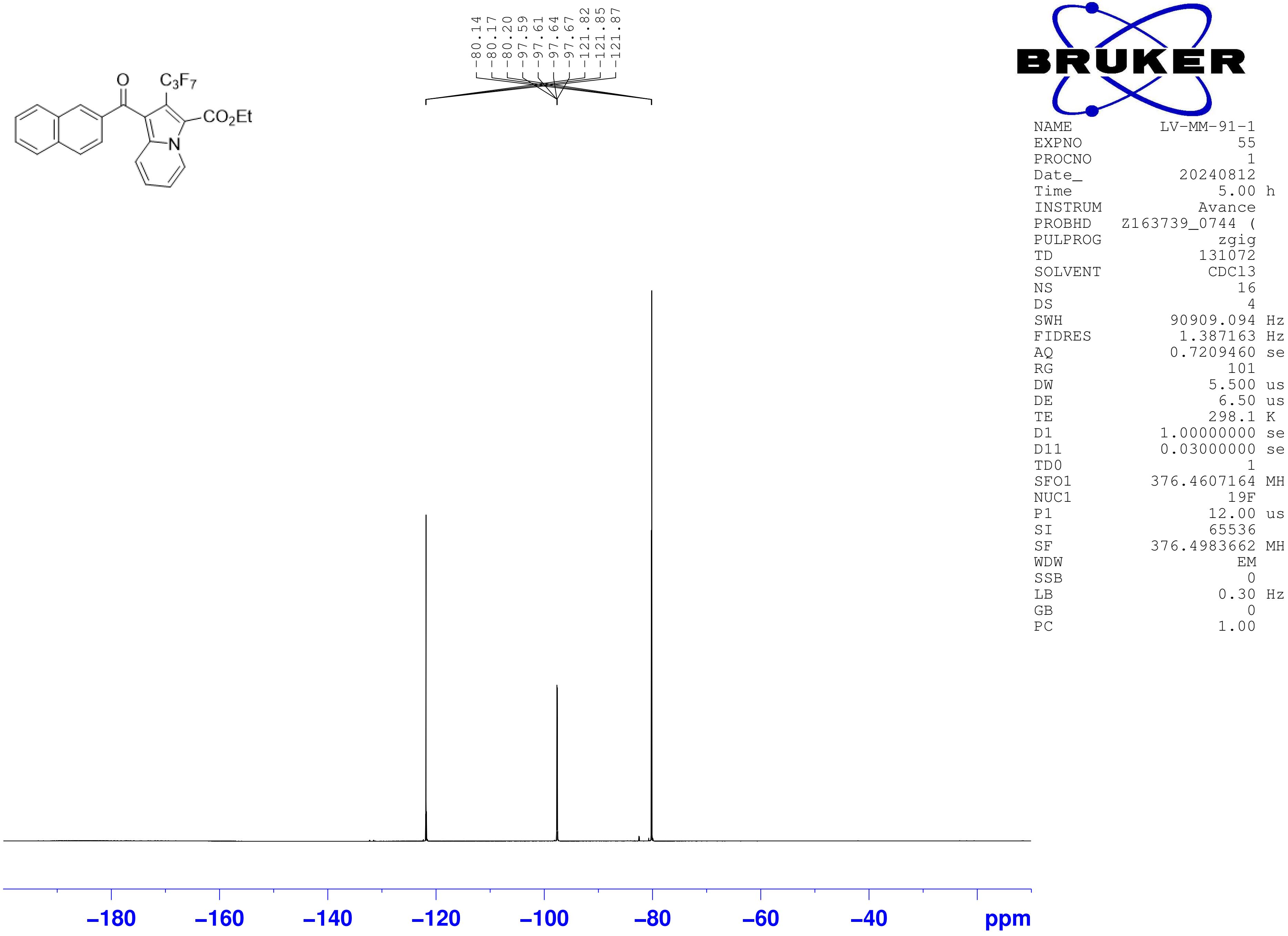
O
C3F7
CO2Et
N
-80.14
-80.17
-80.20
-97.59
-97.61
-97.64
-97.67
-121.82
-121.85
-121.87
BRUKER
NAME LV-MM-91-1
EXPNO 55
PROCNO 1
Date_ 20240812
Time 5.00 h
INSTRUM Avance
PROBHD Z163739_0744 (
PULPROG zgig
TD 131072
SOLVENT CDCl3
NS 16
DS 4
SWH 90909.094 Hz
FIDRES 1.387163 Hz
AQ 0.7209460 se
RG 101
DW 5.500 us
DE 6.50 us
TE 298.1 K
D1 1.00000000 se
D11 0.03000000 se
TD0 1
SFO1 376.4607164 MH
NUC1 19F
P1 12.00 us
SI 65536
SF 376.4983662 MH
WDW EM
SSB 0
LB 0.30 Hz
GB 0
PC 1.00
-180
-160
-140
-120
-100
-80
-60
-40
ppm

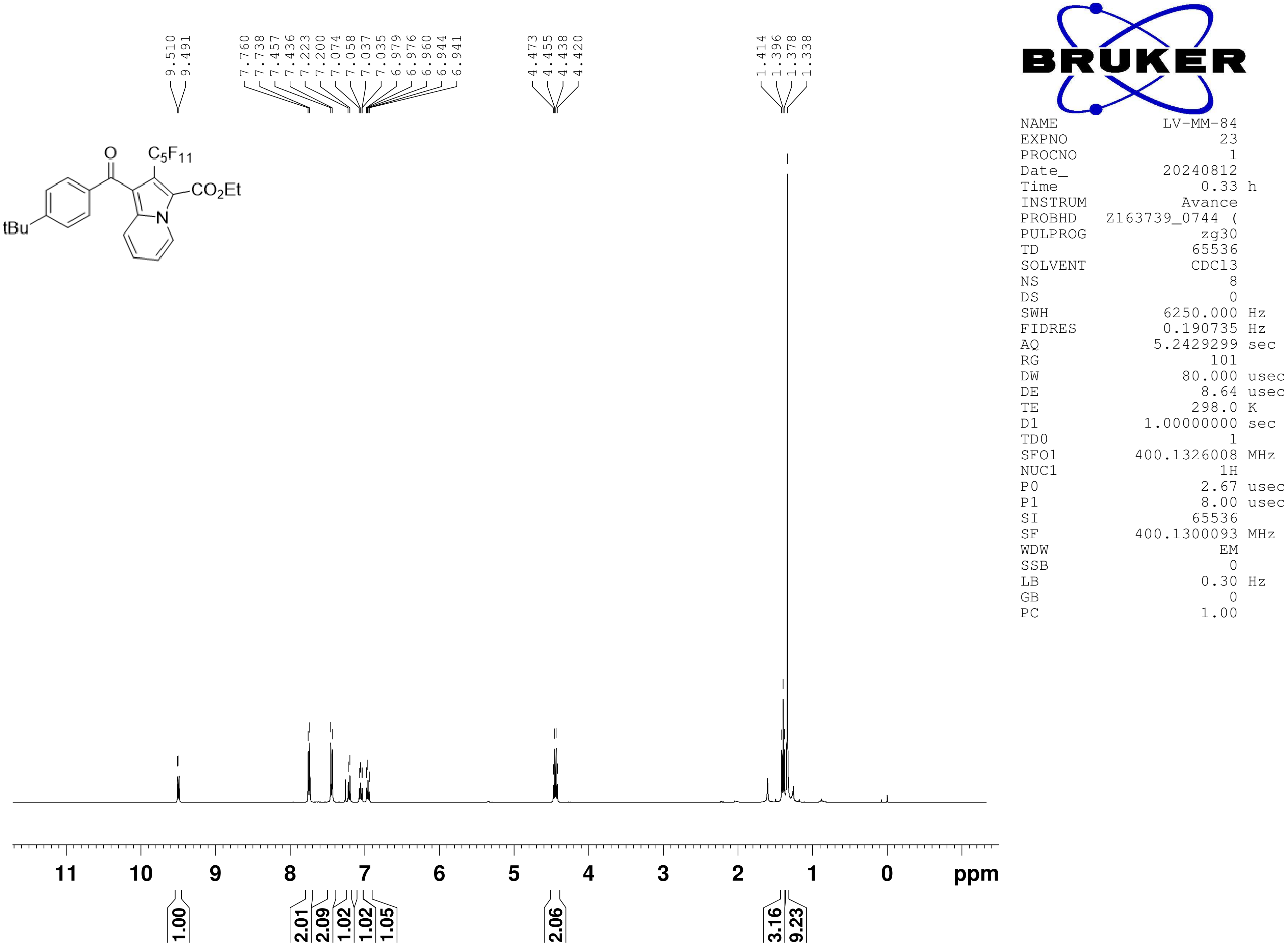
9.510
9.491
7.760
7.738
7.457
7.436
7.223
7.200
7.074
7.058
7.037
7.035
6.979
6.976
6.960
6.944
6.941
4.473
4.455
4.438
4.420
1.414
1.396
1.378
1.338
O
C5F11
CO2Et
tBu
N
BRUKER
NAME LV-MM-84
EXPNO 23
PROCNO 1
Date_ 20240812
Time 0.33 h
INSTRUM Avance
PROBHD Z163739_0744 (
PULPROG zg30
TD 65536
SOLVENT CDCl3
NS 8
DS 0
SWH 6250.000 Hz
FIDRES 0.190735 Hz
AQ 5.2429299 sec
RG 101
DW 80.000 usec
DE 8.64 usec
TE 298.0 K
D1 1.00000000 sec
TD0 1
SFO1 400.1326008 MHz
NUC1 1H
P0 2.67 usec
P1 8.00 usec
SI 65536
SF 400.1300093 MHz
WDW EM
SSB 0
LB 0.30 Hz
GB 0
PC 1.00
11
10
9
8
7
6
5
4
3
2
1
0
ppm
1.00
2.01
2.09
1.02
1.02
1.05
2.06
3.16
9.23

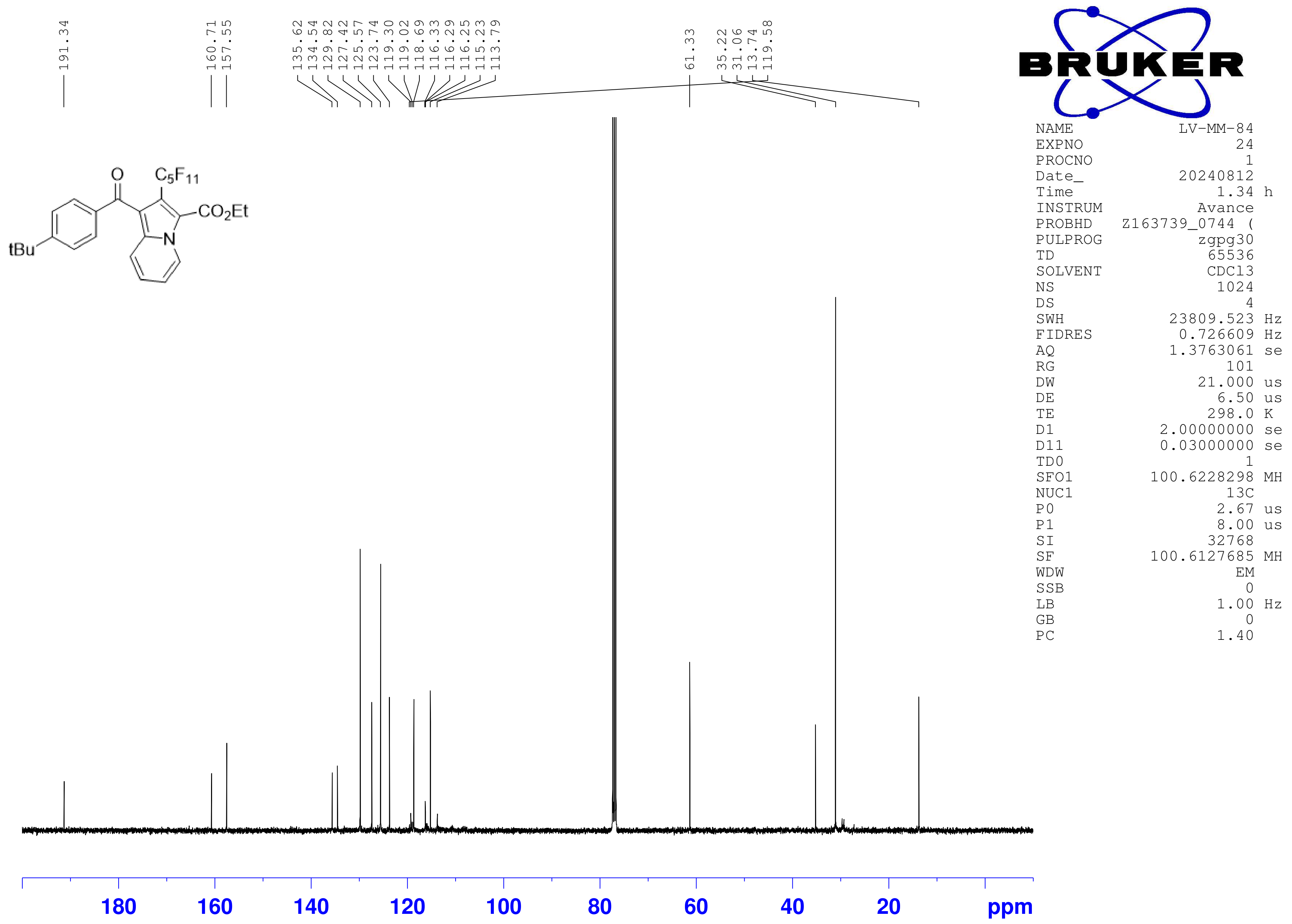

191.34
160.71
157.55
135.62
134.54
129.82
127.42
125.57
123.74
119.30
119.02
118.69
116.33
116.29
116.25
115.23
113.79
61.33
35.22
31.06
13.74
119.58
O
C_5F_{11}
CO_2Et
tBu
N
BRUKER
NAME LV-MM-84
EXPNO 24
PROCNO 1
Date_ 20240812
Time 1.34 h
INSTRUM Avance
PROBHD Z163739_0744 (
PULPROG zgpg30
TD 65536
SOLVENT CDCl3
NS 1024
DS 4
SWH 23809.523 Hz
FIDRES 0.726609 Hz
AQ 1.3763061 se
RG 101
DW 21.000 us
DE 6.50 us
TE 298.0 K
D1 2.00000000 se
D11 0.03000000 se
TD0 1
SFO1 100.6228298 MH
NUC1 13C
P0 2.67 us
P1 8.00 us
SI 32768
SF 100.6127685 MH
WDW EM
SSB 0
LB 1.00 Hz
GB 0
PC 1.40
180
160
140
120
100
80
60
40
20
ppm

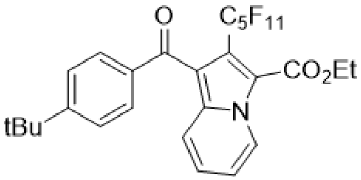

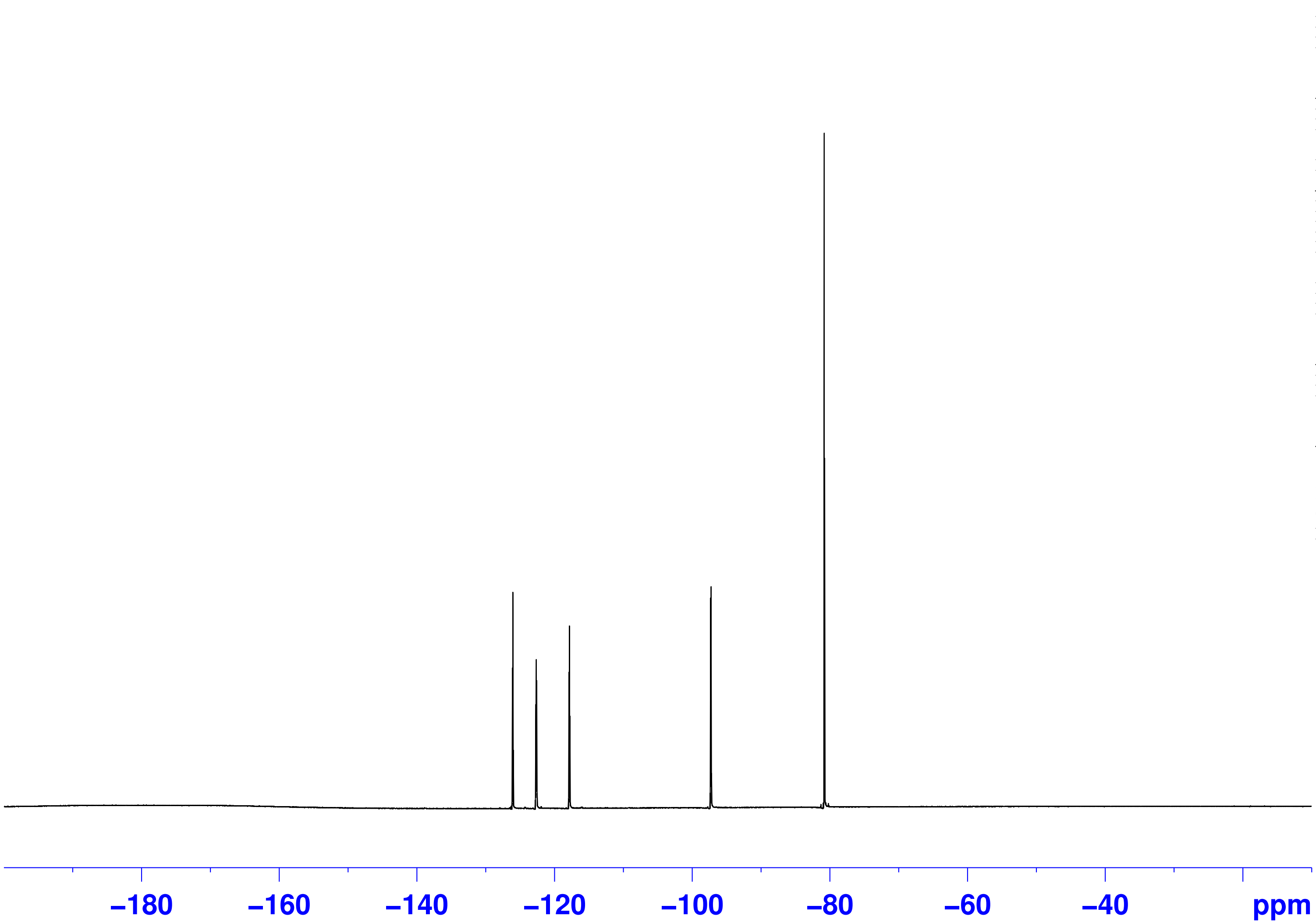

NAME	LV-MM-84	
EXPNO	25	
PROCNO	1	
Date_	20240812	
Time	1.36	h
INSTRUM	Avance	
PROBHD	Z163739_0744 (	
PULPROG	zgig	
TD	131072	
SOLVENT	CDCl3	
NS	16	
DS	4	
SWH	90909.094	Hz
FIDRES	1.387163	Hz
AQ	0.7209460	se
RG	101	
DW	5.500	us
DE	6.50	us
TE	298.1	K
D1	1.00000000	se
D11	0.03000000	se
TD0	1	
SFO1	376.4607164	MH
NUC1	19F	
P1	12.00	us
SI	65536	
SF	376.4983662	MH
WDW	EM	
SSB	0	
LB	0.30	Hz
GB	0	
PC	1.00	

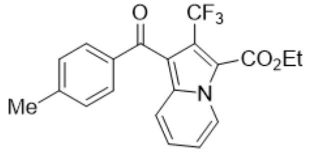

Me
O
CF_3
CO_2Et
N

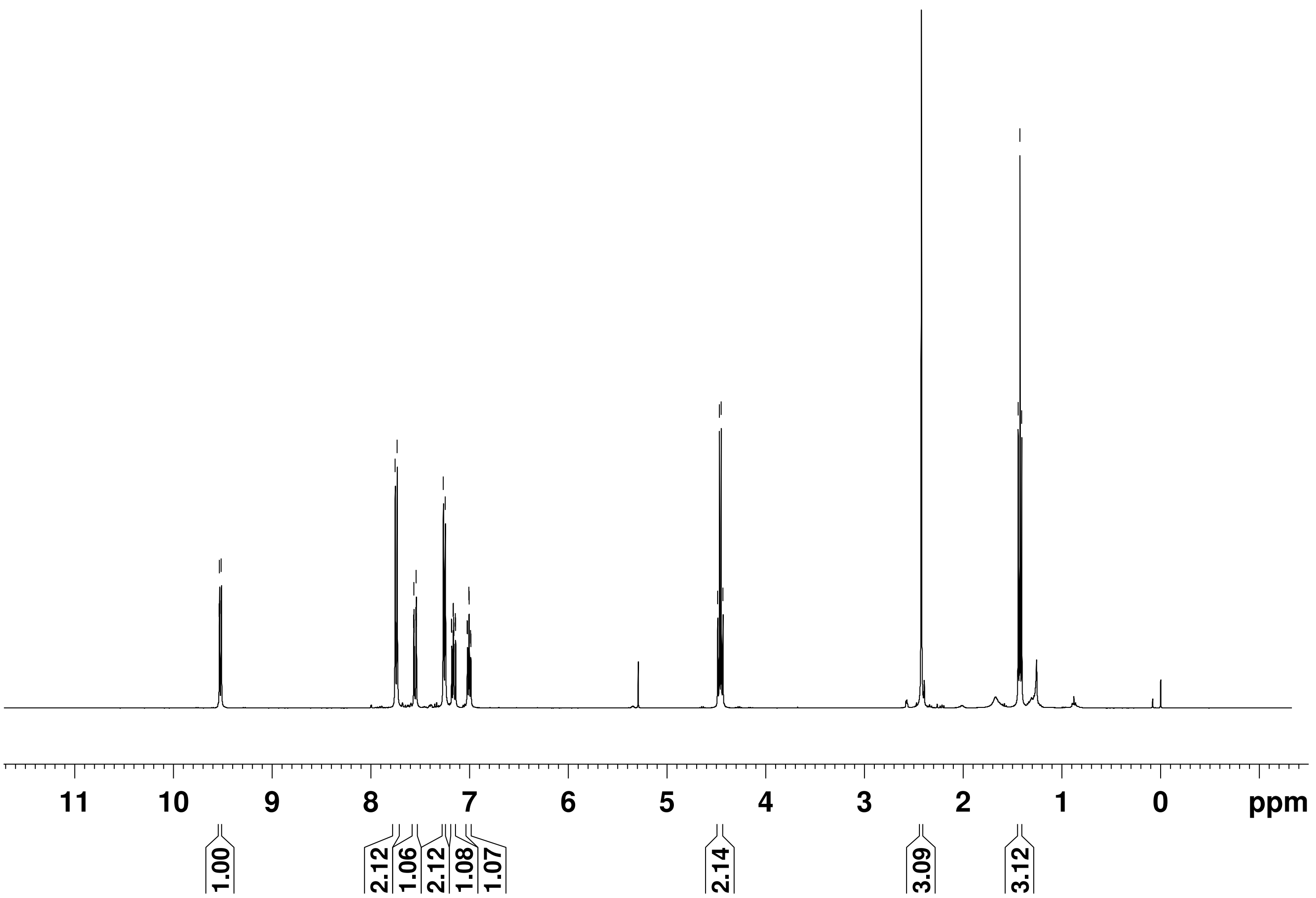

9.537
9.535
9.533
9.519
9.517
9.515
7.755
7.734
7.567
7.565
7.562
7.545
7.542
7.539
7.267
7.264
7.247
7.184
7.181
7.167
7.165
7.162
7.159
7.145
7.142
7.025
7.022
7.007
7.005
6.990
6.987
4.488
4.470
4.452
4.434
2.425
1.443
1.426
1.408
11 10 9 8 7 6 5 4 3 2 1 0 ppm
1.00
2.12
1.06
2.12
1.08
1.07
2.14
3.09
3.12
BRUKER
NAME LV-MM-96-2-20240812
EXPNO 173
PROCNO 1
Date_ 20240812
Time 17.01 h
INSTRUM Avance
PROBHD Z163739_0744 (
PULPROG zg30
TD 65536
SOLVENT CDCl3
NS 8
DS 0
SWH 6250.000 Hz
FIDRES 0.190735 Hz
AQ 5.2429299 sec
RG 90.5
DW 80.000 usec
DE 8.64 usec
TE 298.0 K
D1 1.00000000 sec
TD0 1
SFO1 400.1326008 MHz
NUC1 1H
P0 2.67 usec
P1 8.00 usec
SI 65536
SF 400.1300086 MHz
WDW EM
SSB 0
LB 0.30 Hz
GB 0
PC 1.00

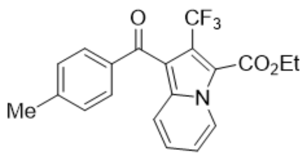

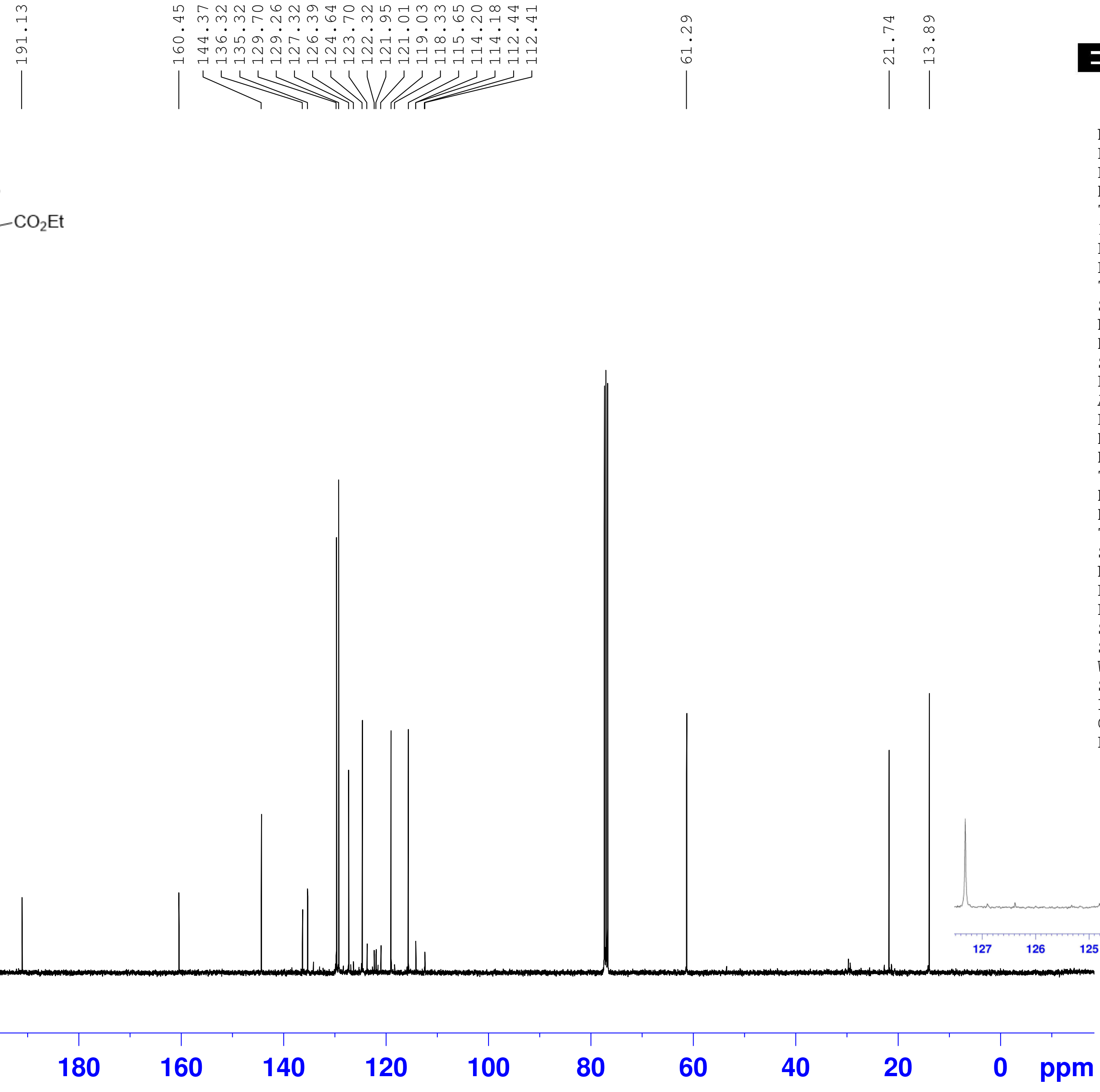

BRUKER

NAME	LV-MM-96-2-202408
EXPNO	174
PROCNO	1
Date_	20240812
Time	17.48 h
INSTRUM	Avance
PROBHD	Z163739_0744 (
PULPROG	zgpg30
TD	65536
SOLVENT	CDCl3
NS	800
DS	4
SWH	23809.523 Hz
FIDRES	0.726609 Hz
AQ	1.3763061 se
RG	101
DW	21.000 us
DE	6.50 us
TE	298.0 K
D1	2.00000000 se
D11	0.03000000 se
TD0	1
SFO1	100.6228298 MH
NUC1	13C
P0	2.67 us
P1	8.00 us
SI	32768
SF	100.6127685 MH
WDW	EM
SSB	0
LB	1.00 Hz
GB	0
PC	1.40

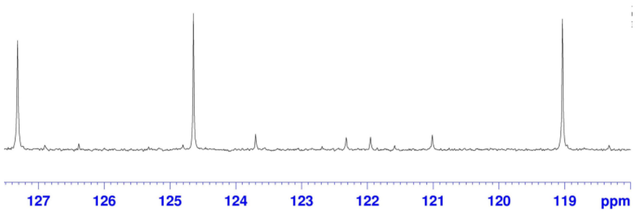

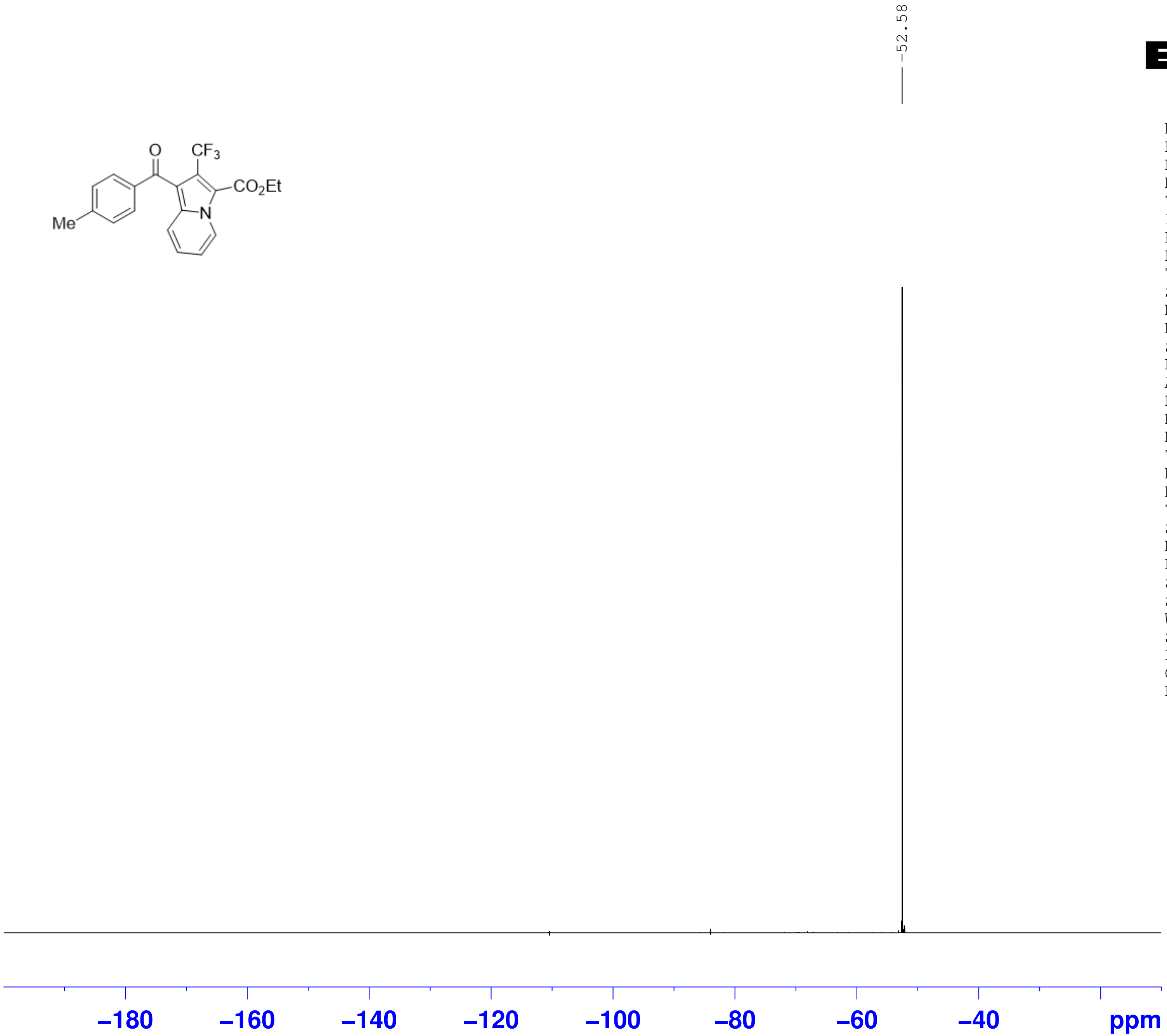

NAME	LV-MM-96-2-202408
EXPNO	175
PROCNO	1
Date_	20240812
Time	17.50 h
INSTRUM	Avance
PROBHD	Z163739_0744 (
PULPROG	zgig
TD	131072
SOLVENT	CDCl3
NS	16
DS	4
SWH	90909.094 Hz
FIDRES	1.387163 Hz
AQ	0.7209460 se
RG	101
DW	5.500 us
DE	6.50 us
TE	298.1 K
D1	1.00000000 se
D11	0.03000000 se
TD0	1
SFO1	376.4607164 MH
NUC1	19F
P1	12.00 us
SI	65536
SF	376.4983662 MH
WDW	EM
SSB	0
LB	0.30 Hz
GB	0
PC	1.00

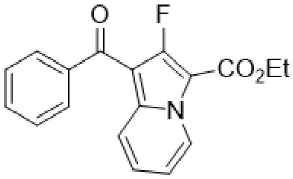

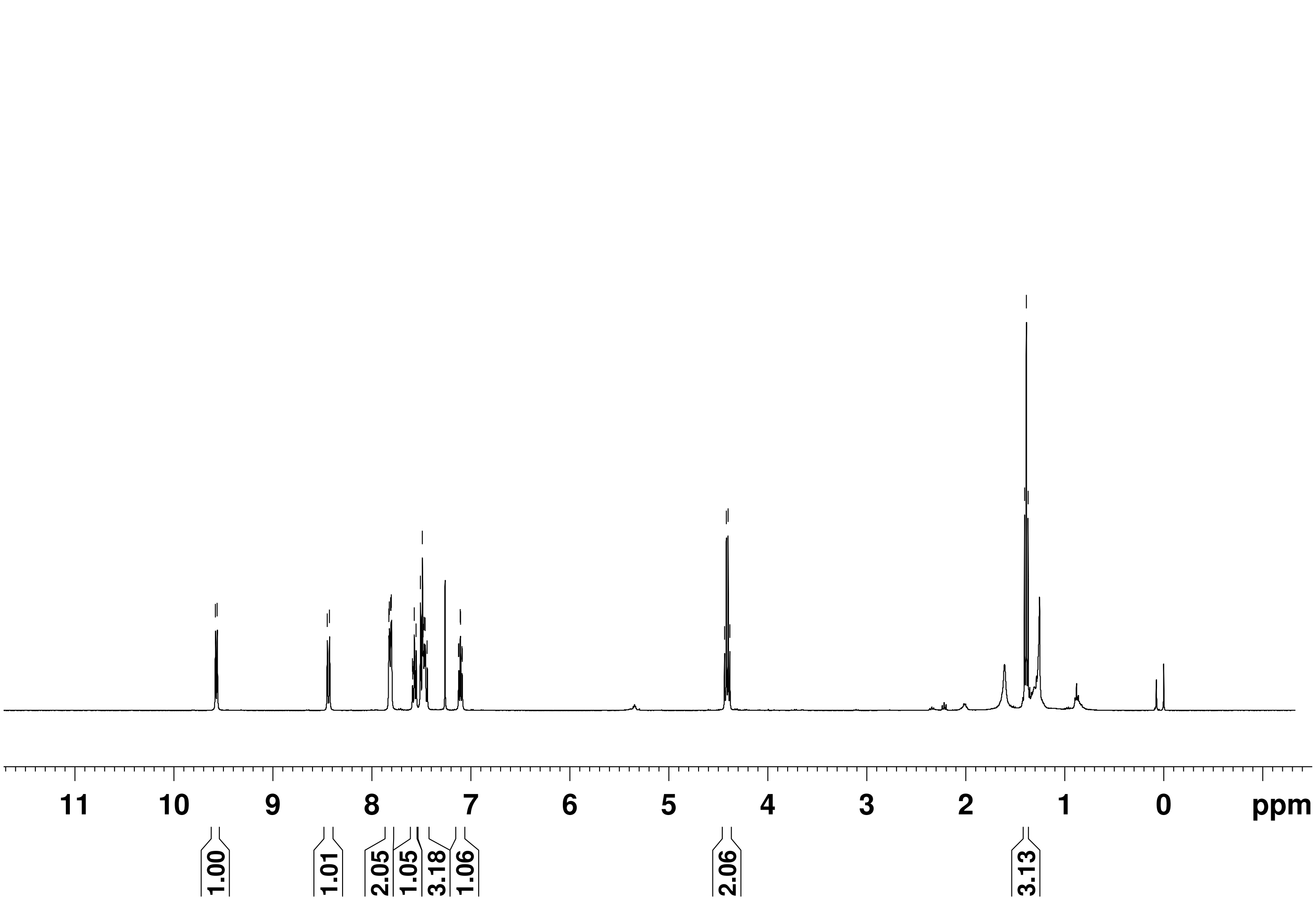

NAME	LV-MM-107-20240813
EXPNO	10
PROCNO	1
Date_	20240813
Time	23.16 h
INSTRUM	Avance
PROBHD	Z163739_0744 (
PULPROG	zg30
TD	65536
SOLVENT	CDCl3
NS	8
DS	0
SWH	6250.000 Hz
FIDRES	0.190735 Hz
AQ	5.2429299 sec
RG	101
DW	80.000 usec
DE	8.64 usec
TE	298.0 K
D1	1.00000000 sec
TD0	1
SFO1	400.1326008 MHz
NUC1	1H
P0	2.67 usec
P1	8.00 usec
SI	65536
SF	400.1300094 MHz
WDW	EM
SSB	0
LB	0.30 Hz
GB	0
PC	1.00

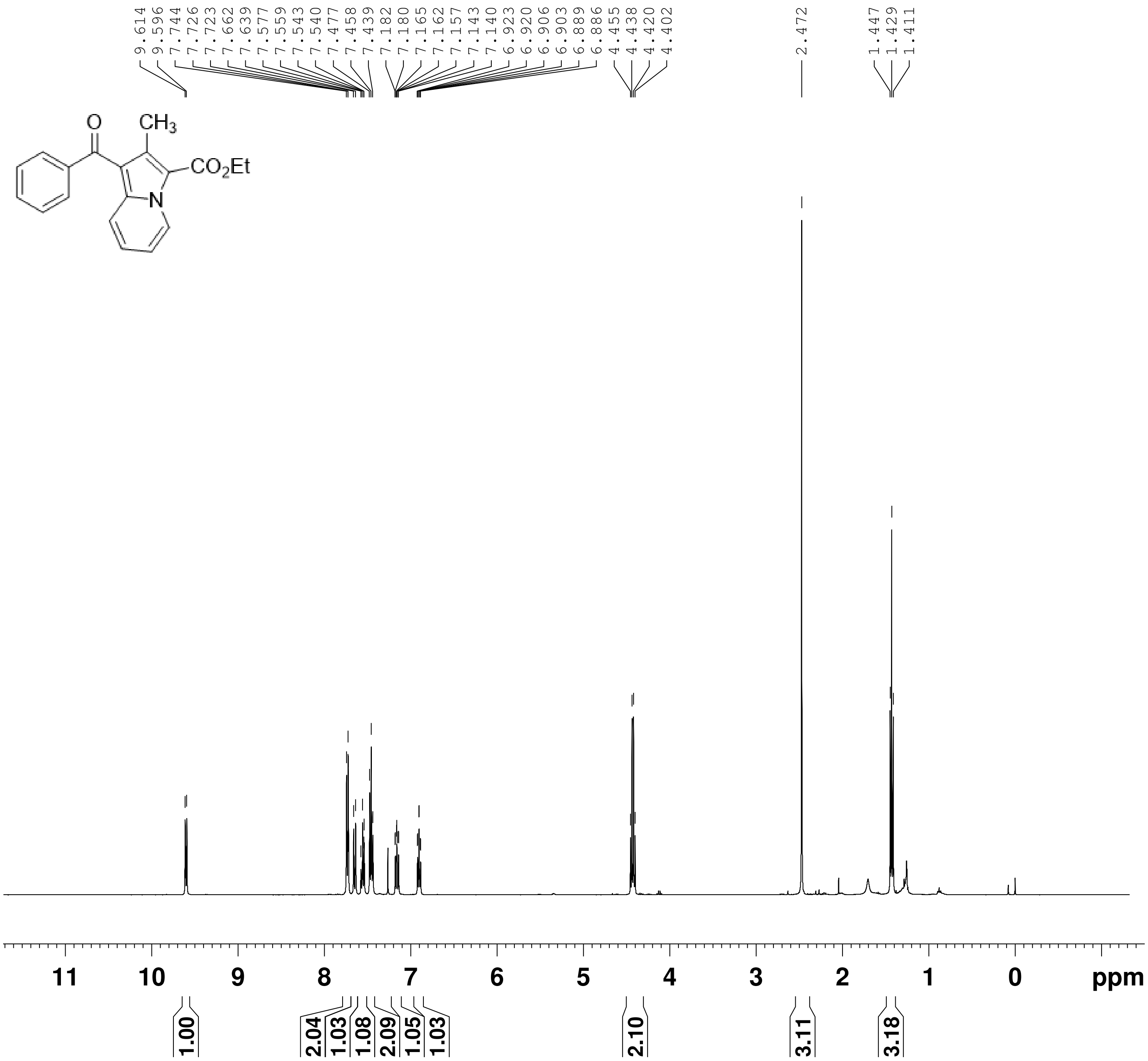

BRUKER

```
NAME       LV-MM-99-20240812
EXPNO                    183
PROCNO                     1
Date_               20240812
Time                 17.55 h
INSTRUM               Avance
PROBHD      Z163739_0744 (
PULPROG                 zg30
TD                     65536
SOLVENT                CDCl3
NS                         8
DS                         0
SWH             6250.000 Hz
FIDRES          0.190735 Hz
AQ             5.2429299 sec
RG                       101
DW               80.000 usec
DE                 8.64 usec
TE                   298.0 K
D1            1.00000000 sec
TD0                        1
SFO1        400.1326008 MHz
NUC1                      1H
P0                 2.67 usec
P1                 8.00 usec
SI                     65536
SF          400.1300078 MHz
WDW                       EM
SSB                        0
LB                   0.30 Hz
GB                         0
PC                      1.00
```

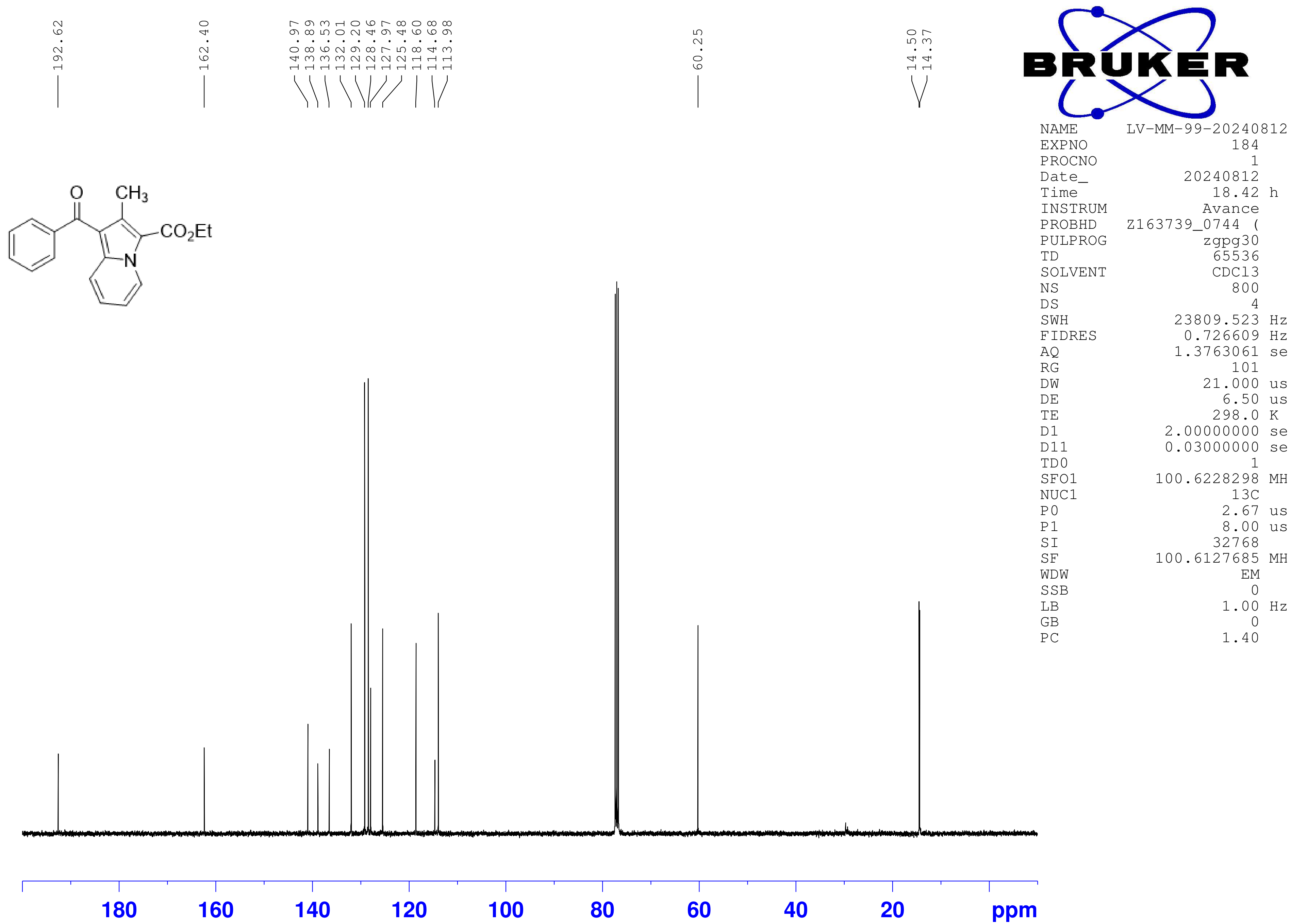
192.62
162.40
140.97
138.89
136.53
132.01
129.20
128.46
127.97
125.48
118.60
114.68
113.98
60.25
14.50
14.37
O
CH3
CO2Et
N
NAME LV-MM-99-20240812
EXPNO 184
PROCNO 1
Date_ 20240812
Time 18.42 h
INSTRUM Avance
PROBHD Z163739_0744 (
PULPROG zgpg30
TD 65536
SOLVENT CDCl3
NS 800
DS 4
SWH 23809.523 Hz
FIDRES 0.726609 Hz
AQ 1.3763061 se
RG 101
DW 21.000 us
DE 6.50 us
TE 298.0 K
D1 2.00000000 se
D11 0.03000000 se
TD0 1
SFO1 100.6228298 MH
NUC1 13C
P0 2.67 us
P1 8.00 us
SI 32768
SF 100.6127685 MH
WDW EM
SSB 0
LB 1.00 Hz
GB 0
PC 1.40
180
160
140
120
100
80
60
40
20
ppm

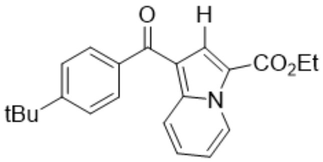

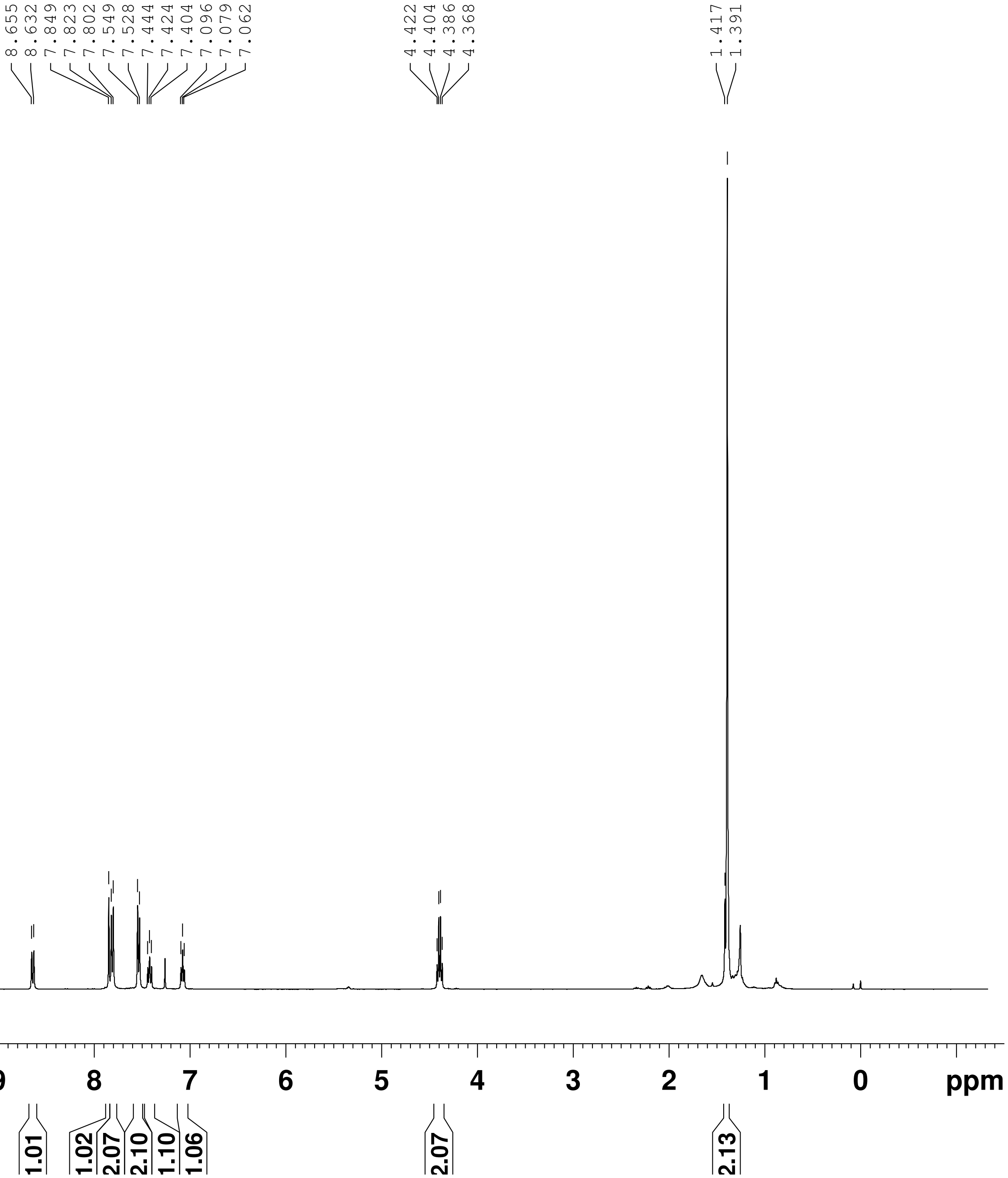

NAME	LV-MM-101-20240812
EXPNO	10
PROCNO	1
Date_	20240812
Time	19.16 h
INSTRUM	Avance
PROBHD	Z163739_0744 (
PULPROG	zg30
TD	65536
SOLVENT	CDCl3
NS	8
DS	0
SWH	6250.000 Hz
FIDRES	0.190735 Hz
AQ	5.2429299 sec
RG	90.5
DW	80.000 usec
DE	8.64 usec
TE	298.0 K
D1	1.00000000 sec
TD0	1
SFO1	400.1326008 MHz
NUC1	1H
P0	2.67 usec
P1	8.00 usec
SI	65536
SF	400.1300094 MHz
WDW	EM
SSB	0
LB	0.30 Hz
GB	0
PC	1.00

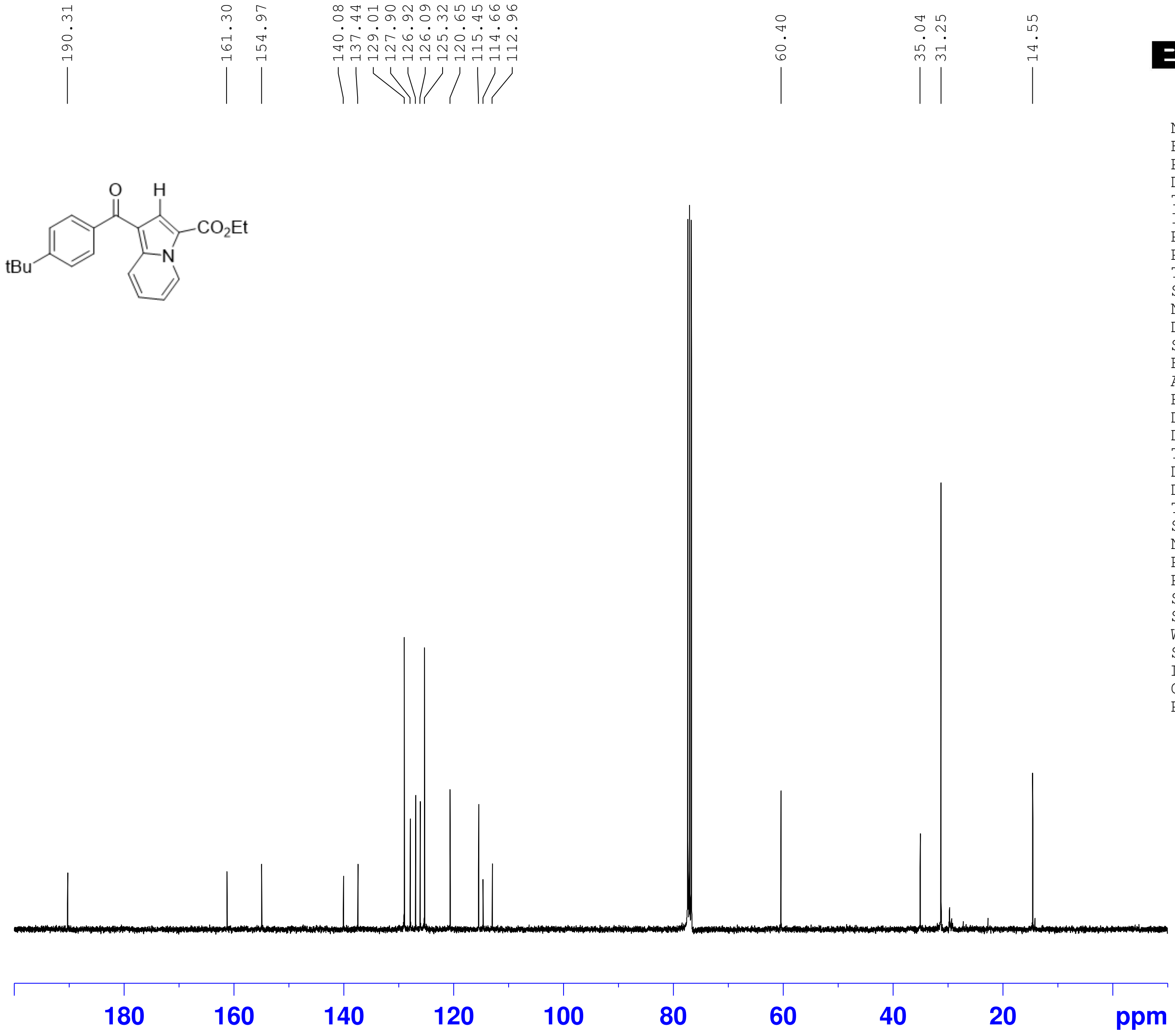

NAME	LV-MM-101-2024081
EXPNO	11
PROCNO	1
Date_	20240812
Time	20.16 h
INSTRUM	Avance
PROBHD	Z163739_0744 (
PULPROG	zgpg30
TD	65536
SOLVENT	CDCl3
NS	1024
DS	4
SWH	23809.523 Hz
FIDRES	0.726609 Hz
AQ	1.3763061 se
RG	101
DW	21.000 us
DE	6.50 us
TE	298.0 K
D1	2.00000000 se
D11	0.03000000 se
TD0	1
SFO1	100.6228298 MH
NUC1	13C
P0	2.67 us
P1	8.00 us
SI	32768
SF	100.6127685 MH
WDW	EM
SSB	0
LB	1.00 Hz
GB	0
PC	1.40

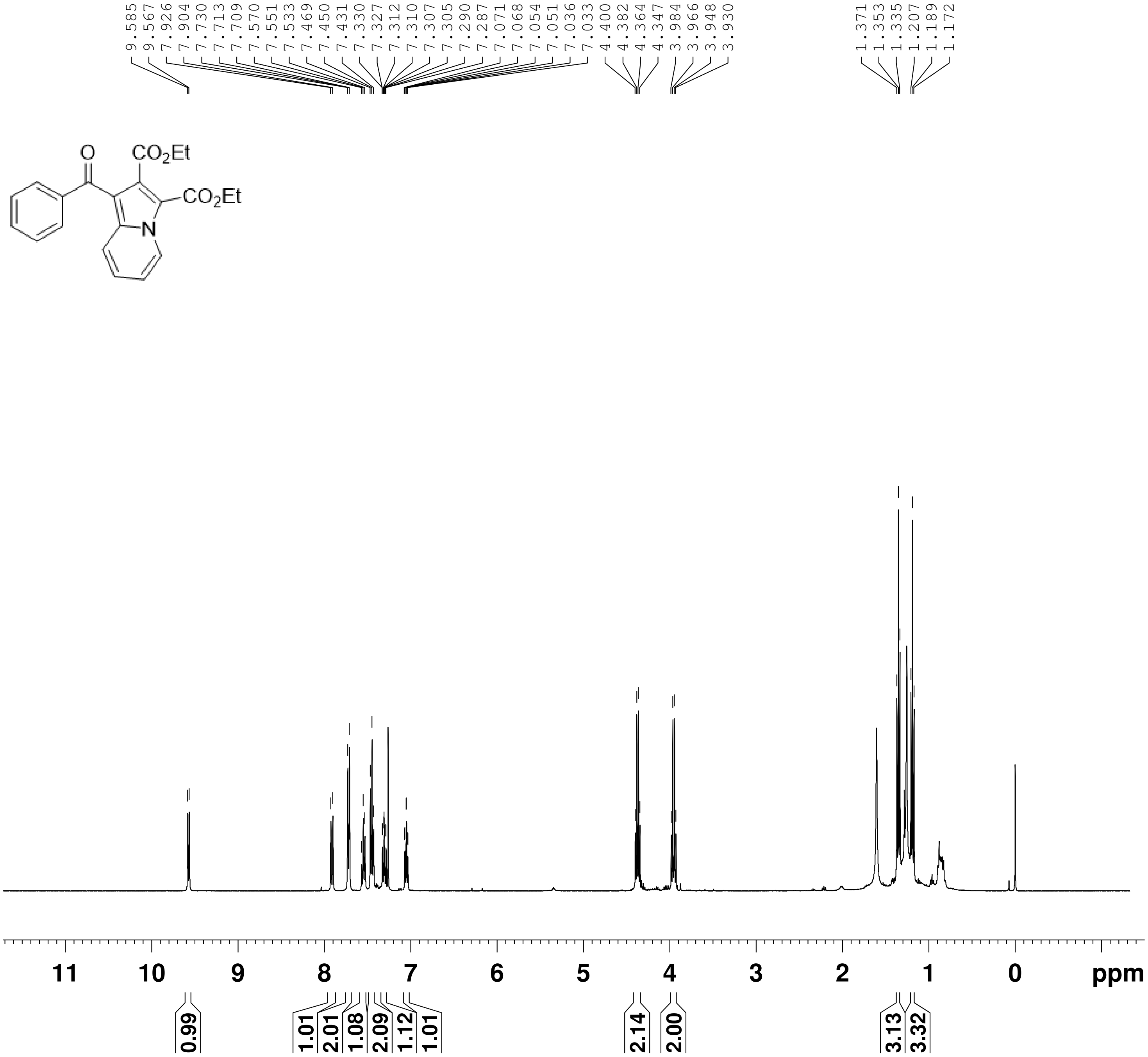

NAME	LV-MM-29-20240730
EXPNO	10
PROCNO	1
Date_	20240730
Time	23.37 h
INSTRUM	Avance
PROBHD	Z163739_0744 (
PULPROG	zg30
TD	65536
SOLVENT	CDCl3
NS	8
DS	0
SWH	6250.000 Hz
FIDRES	0.190735 Hz
AQ	5.2429299 sec
RG	101
DW	80.000 usec
DE	8.64 usec
TE	298.4 K
D1	1.00000000 sec
TD0	1
SFO1	400.1326008 MHz
NUC1	1H
P0	2.67 usec
P1	8.00 usec
SI	65536
SF	400.1300090 MHz
WDW	EM
SSB	0
LB	0.30 Hz
GB	0
PC	1.00

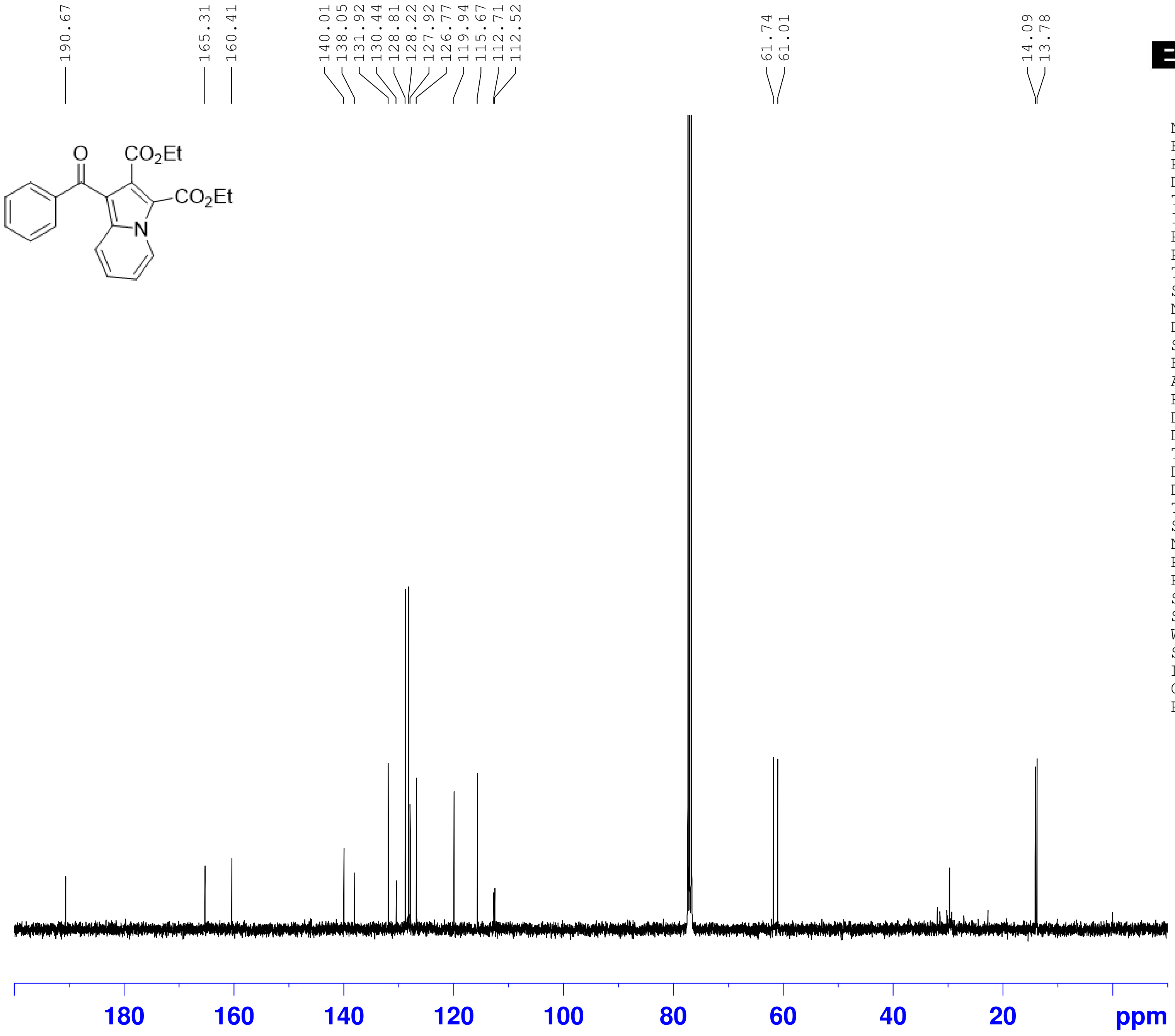

NAME	LV-MM-29-20240804
EXPNO	10
PROCNO	1
Date_	20240804
Time	20.06 h
INSTRUM	Avance
PROBHD	Z163739_0744 (
PULPROG	zgpg30
TD	65536
SOLVENT	CDCl3
NS	800
DS	4
SWH	23809.523 Hz
FIDRES	0.726609 Hz
AQ	1.3763061 se
RG	101
DW	21.000 us
DE	6.50 us
TE	298.0 K
D1	2.00000000 se
D11	0.03000000 se
TD0	1
SFO1	100.6228298 MH
NUC1	13C
P0	2.67 us
P1	8.00 us
SI	32768
SF	100.6127685 MH
WDW	EM
SSB	0
LB	1.00 Hz
GB	0
PC	1.40

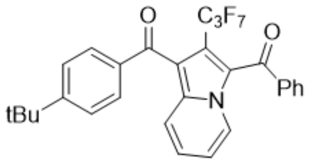

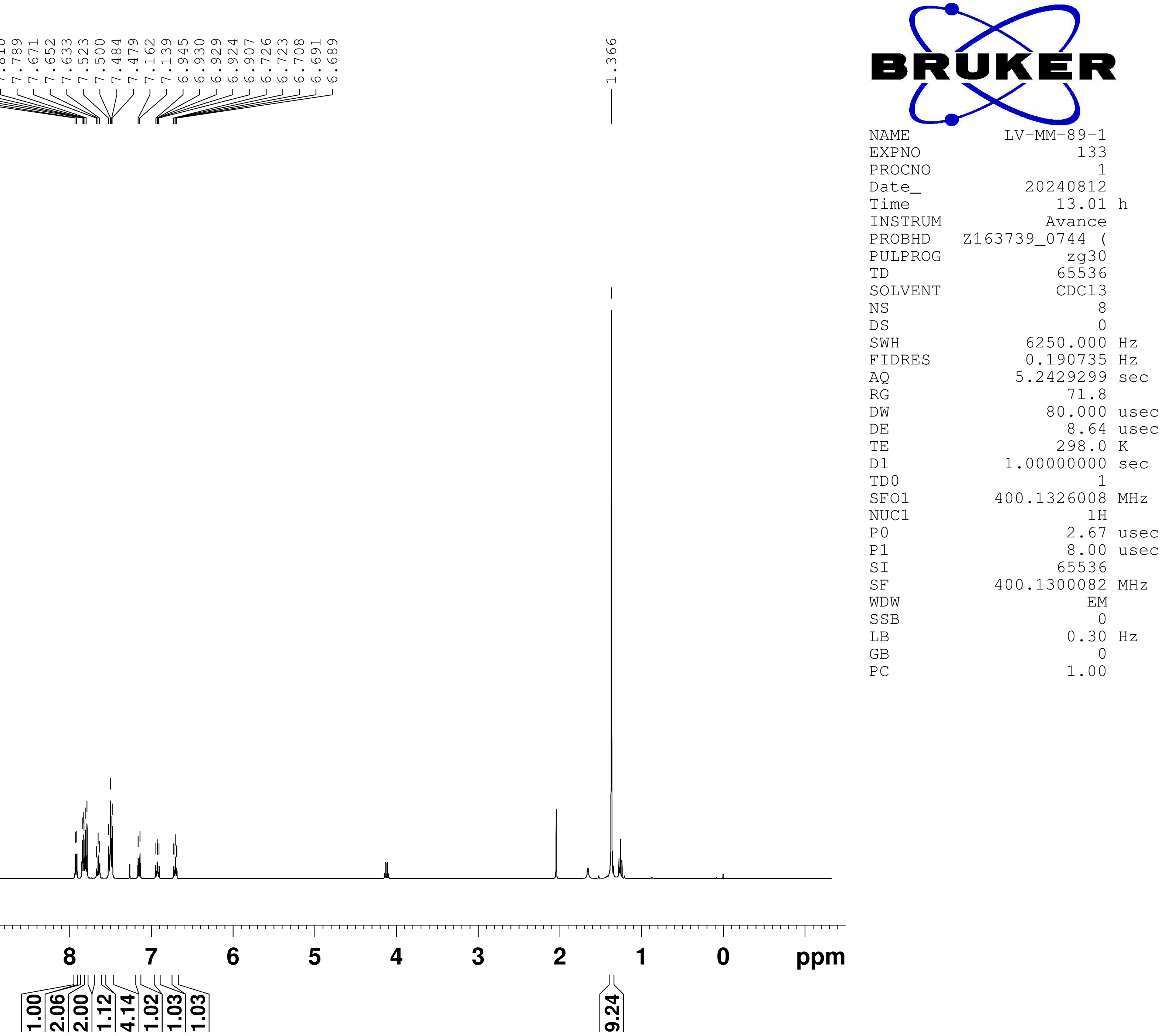
7.931
7.914
7.846
7.828
7.825
7.810
7.789
7.671
7.652
7.633
7.523
7.500
7.484
7.479
7.162
7.139
6.945
6.930
6.929
6.924
6.907
6.726
6.723
6.708
6.691
6.689
1.366
11
10
9
8
7
6
5
4
3
2
1
0
ppm
1.00
2.06
2.00
1.12
4.14
1.02
1.02
1.03
1.03
9.24
NAME LV-MM-89-1
EXPNO 133
PROCNO 1
Date_ 20240812
Time 13.01 h
INSTRUM Avance
PROBHD Z163739_0744 (
PULPROG zg30
TD 65536
SOLVENT CDCl3
NS 8
DS 0
SWH 6250.000 Hz
FIDRES 0.190735 Hz
AQ 5.2429299 sec
RG 71.8
DW 80.000 usec
DE 8.64 usec
TE 298.0 K
D1 1.00000000 sec
TD0 1
SFO1 400.1326008 MHz
NUC1 1H
P0 2.67 usec
P1 8.00 usec
SI 65536
SF 400.1300082 MHz
WDW EM
SSB 0
LB 0.30 Hz
GB 0
PC 1.00

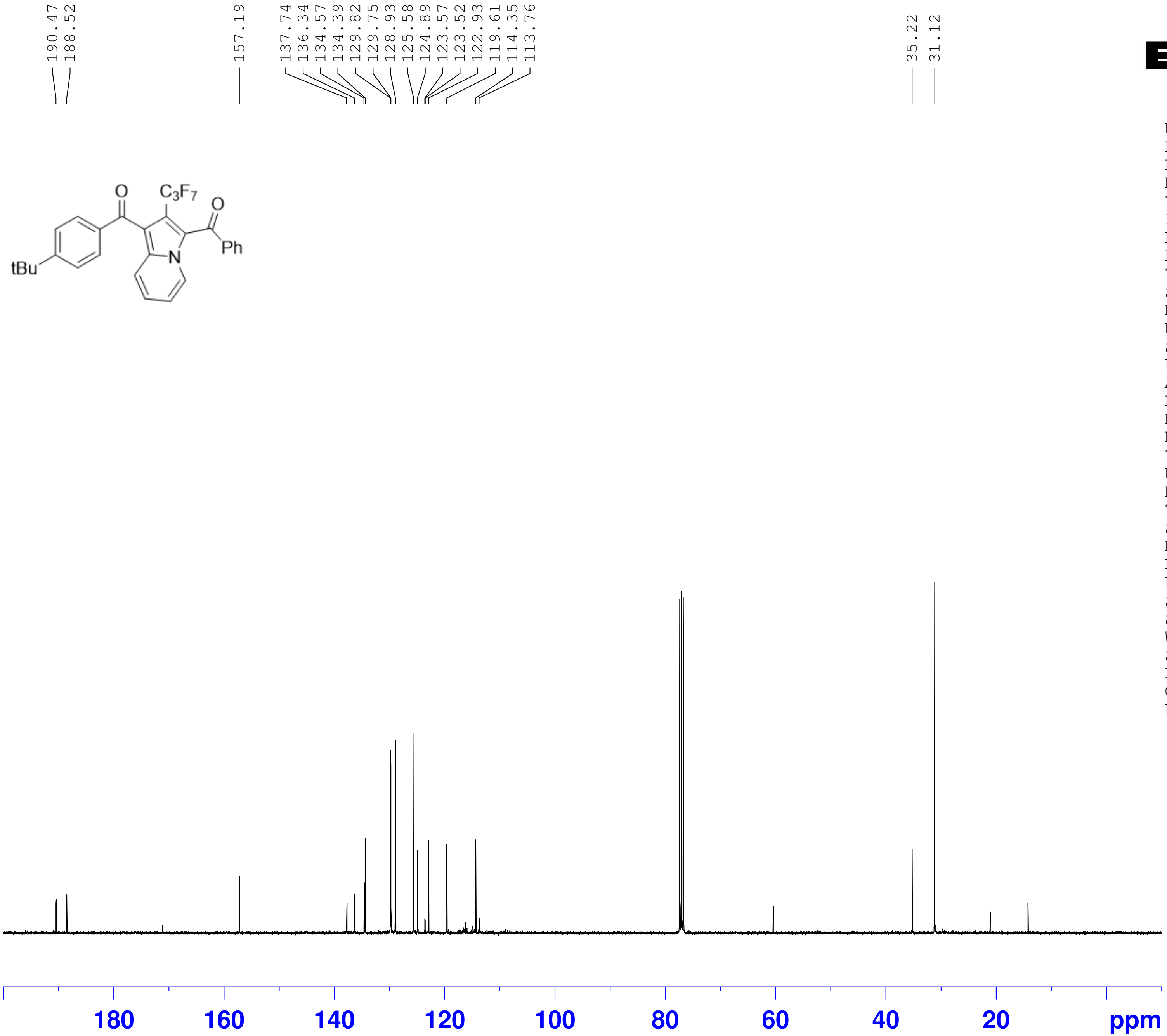

NAME	LV-MM-89-1
EXPNO	134
PROCNO	1
Date_	20240812
Time	14.01 h
INSTRUM	Avance
PROBHD	Z163739_0744 (
PULPROG	zgpg30
TD	65536
SOLVENT	CDCl3
NS	1024
DS	4
SWH	23809.523 Hz
FIDRES	0.726609 Hz
AQ	1.3763061 se
RG	101
DW	21.000 us
DE	6.50 us
TE	298.0 K
D1	2.00000000 se
D11	0.03000000 se
TD0	1
SFO1	100.6228298 MH
NUC1	13C
P0	2.67 us
P1	8.00 us
SI	32768
SF	100.6127685 MH
WDW	EM
SSB	0
LB	1.00 Hz
GB	0
PC	1.40

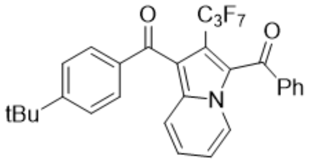

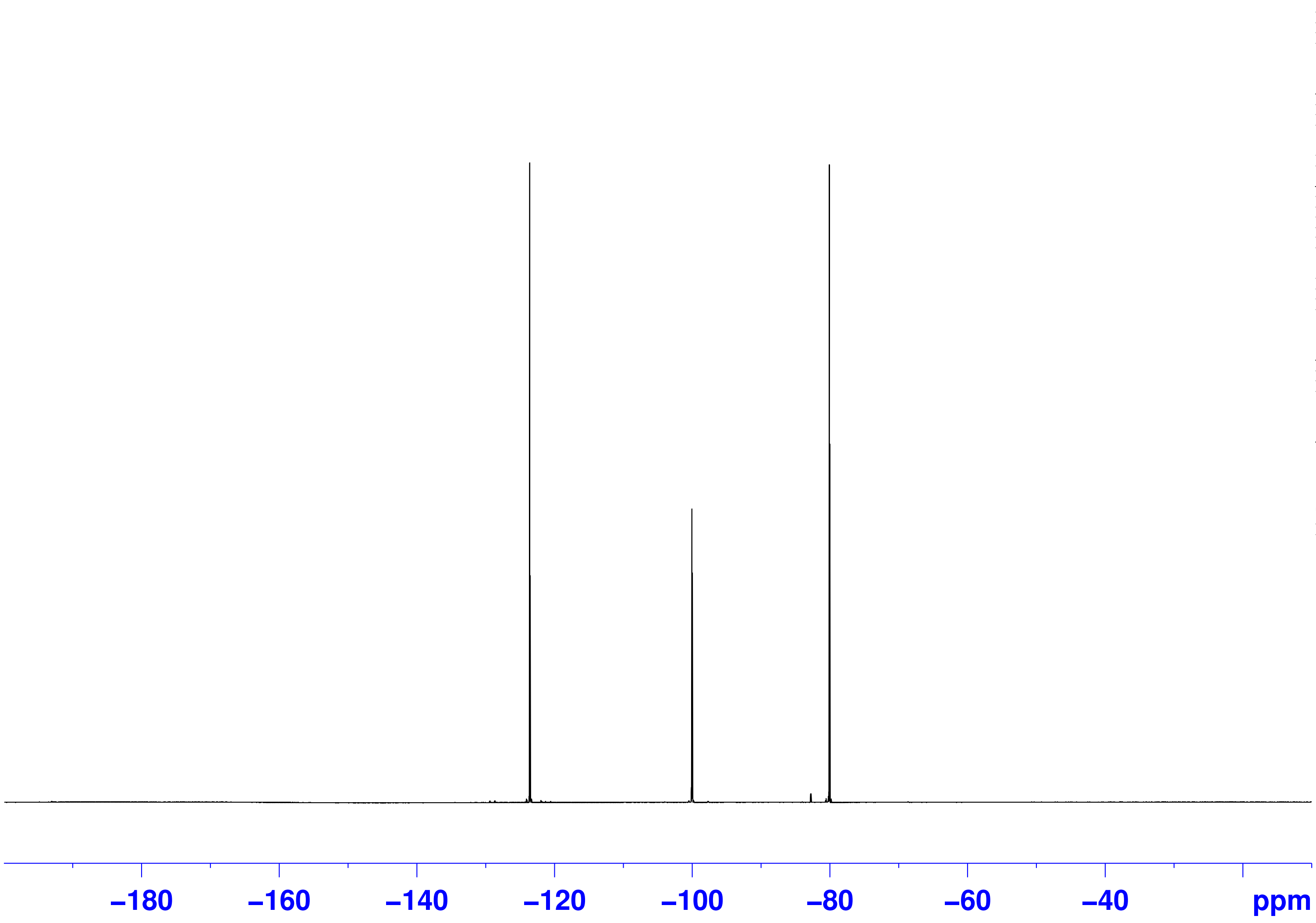

NAME	LV-MM-89-1	
EXPNO	135	
PROCNO	1	
Date_	20240812	
Time	14.03	h
INSTRUM	Avance	
PROBHD	Z163739_0744 (	
PULPROG	zgig	
TD	131072	
SOLVENT	CDCl3	
NS	16	
DS	4	
SWH	90909.094	Hz
FIDRES	1.387163	Hz
AQ	0.7209460	se
RG	101	
DW	5.500	us
DE	6.50	us
TE	298.1	K
D1	1.00000000	se
D11	0.03000000	se
TD0	1	
SFO1	376.4607164	MH
NUC1	19F	
P1	12.00	us
SI	65536	
SF	376.4983662	MH
WDW	EM	
SSB	0	
LB	0.30	Hz
GB	0	
PC	1.00	

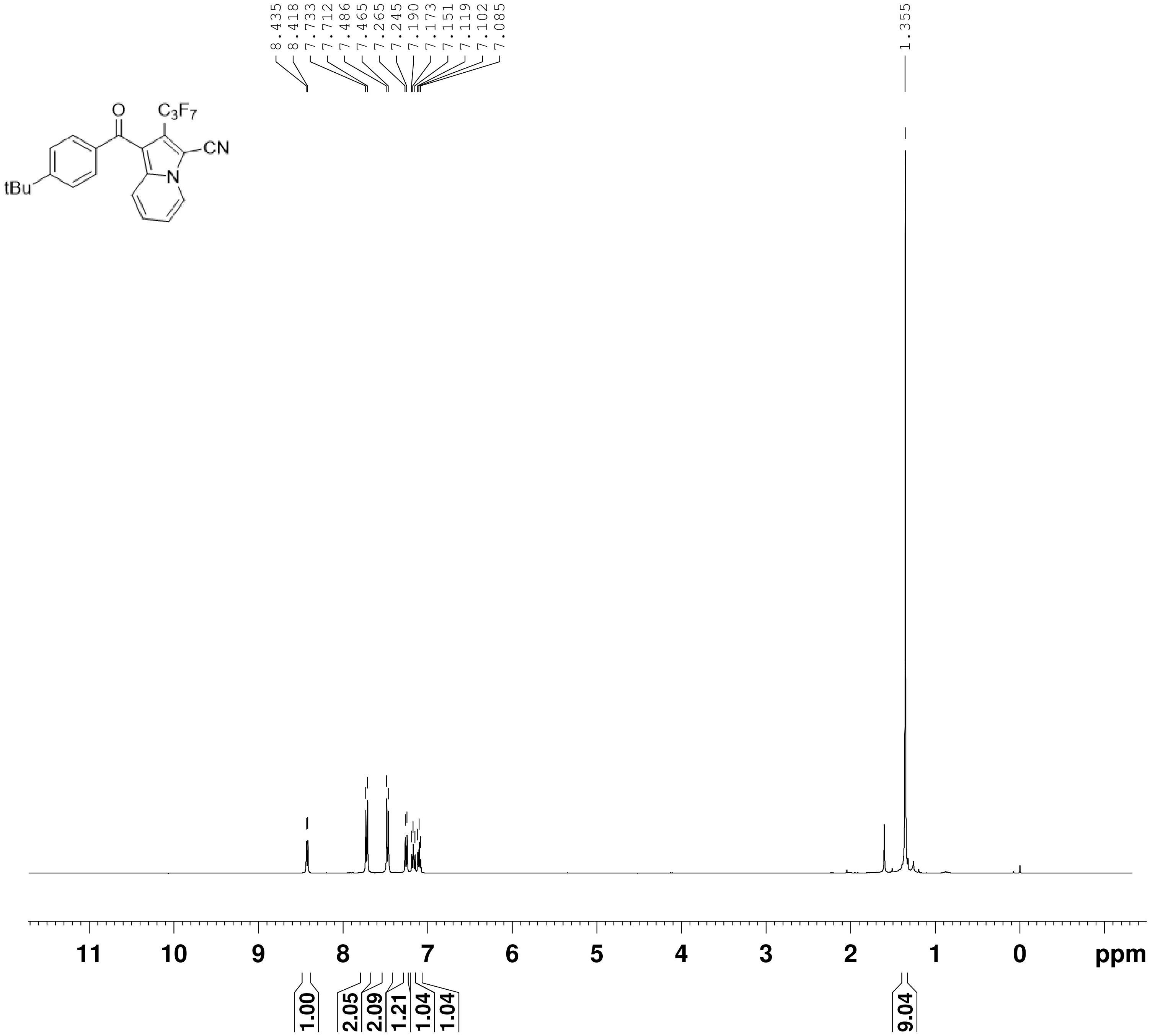

NAME	LV-MM-88-1
EXPNO	83
PROCNO	1
Date_	20240812
Time	7.21 h
INSTRUM	Avance
PROBHD	Z163739_0744 (
PULPROG	zg30
TD	65536
SOLVENT	CDCl3
NS	8
DS	0
SWH	6250.000 Hz
FIDRES	0.190735 Hz
AQ	5.2429299 sec
RG	101
DW	80.000 usec
DE	8.64 usec
TE	298.0 K
D1	1.00000000 sec
TD0	1
SFO1	400.1326008 MHz
NUC1	1H
P0	2.67 usec
P1	8.00 usec
SI	65536
SF	400.1300087 MHz
WDW	EM
SSB	0
LB	0.30 Hz
GB	0
PC	1.00

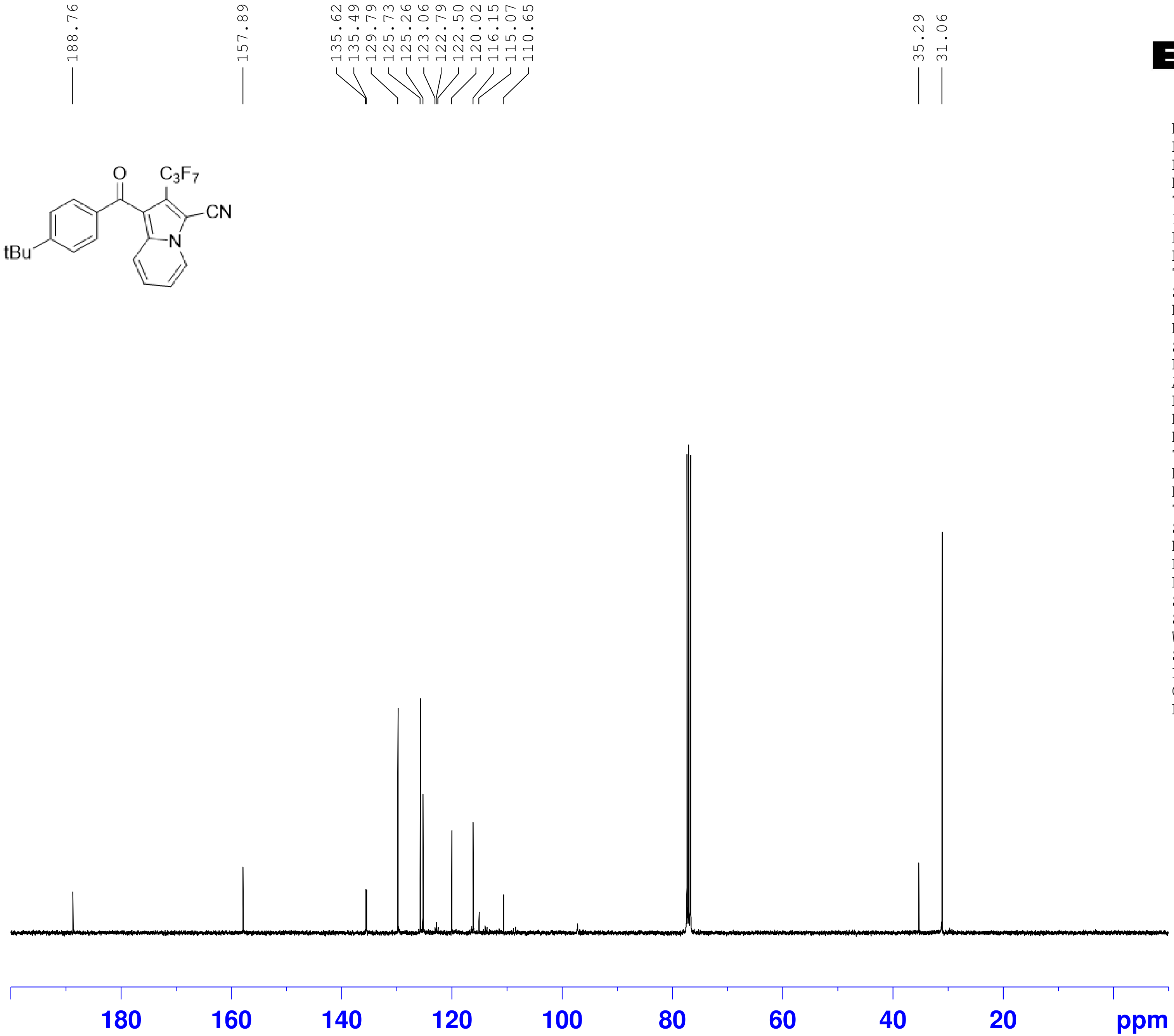

NAME	LV-MM-88-1
EXPNO	84
PROCNO	1
Date_	20240812
Time	8.22 h
INSTRUM	Avance
PROBHD	Z163739_0744 (
PULPROG	zgpg30
TD	65536
SOLVENT	CDCl3
NS	1024
DS	4
SWH	23809.523 Hz
FIDRES	0.726609 Hz
AQ	1.3763061 se
RG	101
DW	21.000 us
DE	6.50 us
TE	298.0 K
D1	2.00000000 se
D11	0.03000000 se
TD0	1
SFO1	100.6228298 MH
NUC1	13C
P0	2.67 us
P1	8.00 us
SI	32768
SF	100.6127685 MH
WDW	EM
SSB	0
LB	1.00 Hz
GB	0
PC	1.40

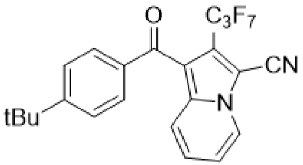

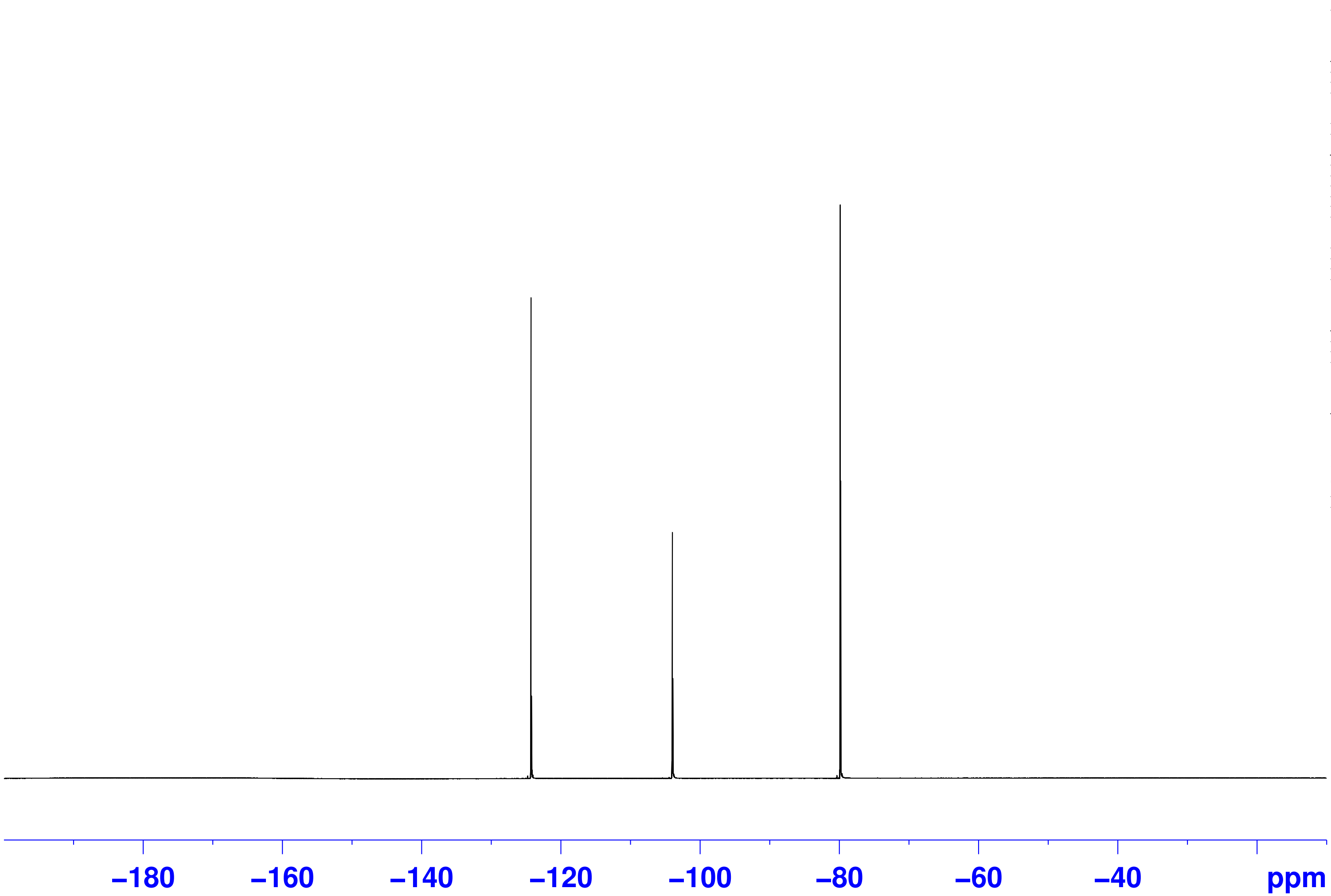

NAME	LV-MM-88-1
EXPNO	85
PROCNO	1
Date_	20240812
Time	8.24 h
INSTRUM	Avance
PROBHD	Z163739_0744 (
PULPROG	zgig
TD	131072
SOLVENT	CDCl3
NS	16
DS	4
SWH	90909.094 Hz
FIDRES	1.387163 Hz
AQ	0.7209460 se
RG	101
DW	5.500 us
DE	6.50 us
TE	298.1 K
D1	1.00000000 se
D11	0.03000000 se
TD0	1
SFO1	376.4607164 MH
NUC1	19F
P1	12.00 us
SI	65536
SF	376.4983662 MH
WDW	EM
SSB	0
LB	0.30 Hz
GB	0
PC	1.00
